# Experience-driven development of decision-related representations in the auditory cortex

**DOI:** 10.1038/s44319-024-00309-0

**Published:** 2024-11-11

**Authors:** Itay Kazanovich, Shir Itzhak, Jennifer Resnik

**Affiliations:** 1 Department of Life Sciences, Ben-Gurion University of the Negev, 84105 Beer Sheva, Israel; 2 Zelman Center for Brain Science Research, Ben-Gurion University of the Negev, 84105 Beer Sheva, Israel

**Keywords:** Auditory Cortex, Choice, Two-photon Calcium Imaging, Neuroscience

## Abstract

Associating sensory stimuli with behavioral significance induces substantial changes in stimulus representations. Recent studies suggest that primary sensory cortices not only adjust representations of task-relevant stimuli, but actively participate in encoding features of the decision-making process. We sought to determine whether this trait is innate in sensory cortices or if choice representation develops with time and experience. To trace choice representation development, we perform chronic two-photon calcium imaging in the primary auditory cortex of head-fixed mice while they gain experience in a tone detection task with a delayed decision window. Our results reveal a progressive increase in choice-dependent activity within a specific subpopulation of neurons, aligning with growing task familiarity and adapting to changing task rules. Furthermore, task experience correlates with heightened synchronized activity in these populations and the ability to differentiate between different types of behavioral decisions. Notably, the activity of this subpopulation accurately decodes the same action at different task phases. Our findings establish a dynamic restructuring of population activity in the auditory cortex to encode features of the decision-making process that develop over time and refines with experience.

## Synopsis

Tracking the activity in the auditory cortex as mice gain experience in a tone detection task with a delayed decision window reveals dynamic restructuring of population activity. This reorganization encodes decision-making features that evolve over time and become more refined with task experience.

- Task experience progressively increases choice-dependent activity, primarily in non-sensory cells of the auditory cortex, reflecting growing task familiarity.
- Motor signals representing behavioral choices in the auditory cortex are gated by their relevance to the auditory task during performance.

## Abstract (web summary)

Tracking the activity in the auditory cortex as mice gain experience in a tone detection task with a delayed decision window reveals dynamic restructuring of population activity. This reorganization encodes decision-making features that evolve over time and become more refined with task experience.

## Graphical abstract

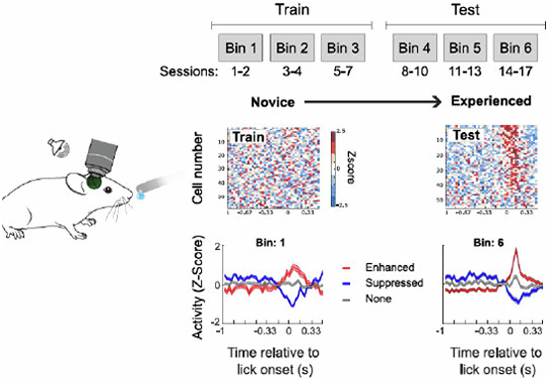

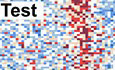

## Introduction

Our understanding of the role played by sensory cortices in decision-making has undergone significant refinement in the last two decades through studies in behaving animals. Departing from the conventional feedforward model, where primary sensory areas were viewed as static entities primarily responsible for extracting and encoding the physical attributes of stimuli before transmitting them to higher cortical areas, our perspective now acknowledges the dynamic nature of information representation in these primary sensory regions. We recognize that the information encoded in primary sensory areas, such as the auditory or visual cortex, is heavily influenced by task demands and expectations, with stimulus representations undergoing substantial changes as animals learn the association between sensory stimuli and their behavioral significance (Recanzone et al, 1993; Bagur et al, 2018; David et al, 2012; Lee and Middlebrooks, 2011; Rodgers and DeWeese, 2014; Bao et al, 2004; Polley et al, 2006; Poort et al, 2015; Francis et al, 2018; Guo et al, 2019; Rutkowski and Weinberger, 2005; Fritz et al, 2003; Li et al, 2004; Jaramillo and Zador, 2011; Carcea et al, 2017; Brosch et al, 2011; Mohn et al, 2021; Froemke et al, 2013; Blake et al, 2002; Otazu et al, 2009). These changes enhance and refine representations for task-relevant stimuli, thereby improving the salience of information transmitted to downstream areas (Gilbert and Sigman, 2007; Zhang and Xu, 2022).

Recent studies also show that primary sensory cortices in expert animals are not merely adjusting representations of task-relevant stimuli but are actively involved in encoding decision-making processes (Rodgers and DeWeese, 2014; Francis et al, 2018; Guo et al, 2019). For instance, reward expectation and choice direction, two main aspects of decision-making, can modulate the primary auditory cortex (ACtx) neural activity in periods when no auditory stimulus is present (Guo et al, 2019; Jaramillo and Zador, 2011; Brosch et al, 2011; Carcea et al, 2017).

The challenge arises when attempting to separate the distinct stages through which sensory information is transformed into a decision, encompassing sensation, decision formation, preparatory motor output, and the eventual behavioral response. In classical learning paradigms, where animals must make decisions based on specific sensory features, such as licking left or right when they detect a specific sound to get a water reward, the activity is typically measured within the stimulus window or immediately after it (Francis et al, 2018; Liu et al, 2021). This makes it challenging to separate the activity evoked by the stimulus, the decision-making process, and the subsequent behavioral output. In freely-moving two-alternative choice tasks, where the animal has to move toward a port to get the reward, there is a separation between stimulus and response, but usually, periods of movement are tested against periods of quiet waiting (Jaramillo and Zador, 2011), making it difficult to separate the motor from the decision and reward expectation process.

To address this challenge, decision tasks incorporating delay periods have been widely used in non-human primates to investigate choice-dependent activity in higher cortical areas. Studies introducing a delay period between the stimulus and the behavioral output that examined the activity in sensory regions, have uncovered enhanced or suppressed neuronal activity in primary sensory areas of expert mice, such as the visual cortex (Goard et al, 2016) and the barrel cortex (Guo et al, 2014). These findings suggest an active involvement of sensory cortices in encoding aspects of decision-making processes, prompting intriguing questions about the development of perceptual decision representations within these regions. Is choice representation in sensory cortices an innate trait, or does it emerge with task experience? Moreover, is it primarily influenced by motor actions such as licking, or by task rules and accumulated experience?

If changes in activity are experience-dependent, one would anticipate alterations in activity, at a single cell and network level, over time, as the mouse becomes experienced with the task and his new surroundings. For example, in a novice mouse with limited comprehension or predictive abilities concerning when a lickspout will dispense water, we would expect some degree of innate lick-evoked or motor-related activity in the sensory cortices (Morandell et al, 2024; Clayton et al, 2021; Vinck et al, 2015). Then, as the mouse gains experience with the task, we would expect to find a progressive alteration in activity surrounding the lick, reflecting the mouse’s growing understanding of task rules and the integration of decision-making processes with the act of licking. Conversely, if choice representation in sensory cortices is primarily driven by motor-related activity (Clayton et al, 2021; Schneider et al, 2014, 2018), cortical modulation would likely be evident from the outset, when the mouse starts licking, showing minimal change even as task rules evolve.

To test whether experience drives the development of behavioral decision representation in sensory cortices, we devised a behavioral task for head-fixed mice incorporating a delay period between stimulus presentation and behavioral choice. Employing calcium imaging, we monitored single-cell activity in the auditory cortex while mice familiarized themselves with the task. Following the activity of the auditory cortex for more than two weeks of training and testing, revealed a progressive increase in activity starting before the lick within a specific sub-population of neurons as mice became experienced with the task. This activity was task-dependent, was modulated by changing task rules, and wasn’t driven by changes in lick dynamics. Furthermore, task experience correlated with heightened synchronized activity in these populations, especially during successful trials, accompanied by a gradual improvement in the ability to differentiate between different behavioral choices. Notably, the activity of this subpopulation accurately decoded whether a mouse licked before or after the sound stimulus, highlighting the development of behavioral choice representation in a primary sensory cortical area through task experience.

## Results

### Mice gain experience in an auditory detection delay task

**Figure 1:**
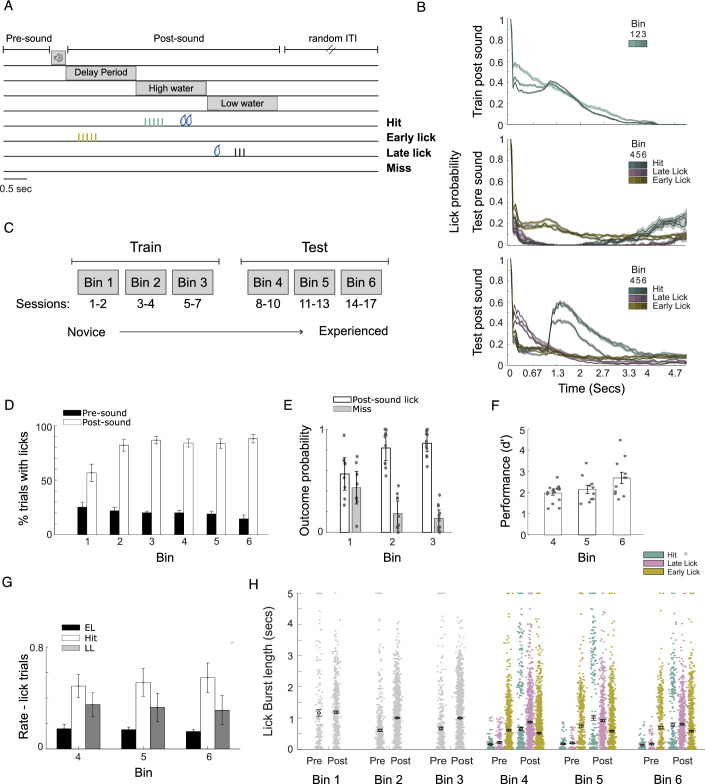
Mice gain experience in an auditory detection delay task. (**A**) Task outline: Six animals were required to detect a tone and wait for 1.5 s before licking. After waiting, they had 1.5 s to lick the lickspout to receive a large dose (12 μL) of sweetened water (Hit). An early lick (EL) during the delay period led to a timeout with no water. Mice could refrain from licking until the end of the high-water period and receive a small water droplet (4 μL of water, late lick period - LL). Failure to lick throughout the trial was counted as a Miss. (**B**) Lick probabilities after the first lick in the different Bins and the different behavioral outputs: Hit, Late Lick, and Early Lick. (**C**) Division of the sessions to bins according to task experience in the training and testing phases. (**D**) Likelihood of licking before or after the auditory cue. During the training phase, mice started licking after the sound more often and less often in the pre-sound period (two-way ANOVA for Bins 1–3, bin x period interaction F = 8.38, *p* = 7e^−04^ and for Bins 1–6 bin x period interaction F = 5.8, *p* = 0.0001, *n* = 66 sessios). Values represent mean ± se across sessions. (**E**) Likelihood of licking following the auditory cue. During the training phase, mice exhibited an increased frequency of licking following the auditory cue, resulting in a reduction of missed responses (*n* = 30 sessions and 60 hit and miss rates, 1e two-way ANOVA bin x type of trial interaction F = 16.24, *p* = 3e^−06^). Values represent mean ± se across sessions. (**F**) Behavioral performance in the testing phase: d prime increased as mice gained experience with the task (*n* = 36 sessions). Values represent mean ± se across sessions. (**G**) Performance rate for the different behavioral outputs during testing; in trials that the mice licked after the sound (*n* = 3 outcome possibilities for each of the 36 sessions). Values represent mean ± se across sessions. (**H**) Length of lick burst for in the pre- and post-sound periods in the different Bins and trial outcomes (*n* = 14,450 licks). Lick bursts became shorter between training and testing and lick bursts in the post-sound period were generally longer than those in the pre-sound period. Values represent mean ± se. Source data are available online for this figure.

To study the development of behavioral choice representation in sensory cortices, we performed chronic two-photon calcium imaging in the ACtx of awake, head-fixed mice that expressed GCaMP6s non-selectively in L2/3 neurons (*n* = 4335 Cells/*N* = 6 Mice) while mice gained experience with a tone detection task with a delayed decision window (Fig. 1A). To disentangle the encoding of auditory stimuli and subsequent behavioral choice, we presented mice randomly with one of two auditory cues (6 or 16 kHz tone at 60 dB SPL). The mice were required to detect the tone and delay their response for 1.5 s. Following this delay period, they had 1.5 s to lick the water lickspout. If mice managed to detect the sound and delay their response (Hit) a big rewarding dose of sweetened water was dispensed 1 s after the lick (Fig. 1A,B). By introducing a delay in water delivery, we could analyze activity surrounding the lick independently from water-evoked activity. An early lick (EL) during the delay response window led to no water and a timeout. The mice could wait until the end of the high-water period without licking, and a small water droplet would be dispensed (late lick period - LL). This could be a valid, and potentially simpler strategy, with no need to calculate the delay accurately, but it offered less water. Licking in catch trials where no sound was presented was counted as a False Alarm (FA) and refraining from licking during the trials where the sound was presented was counted as a Miss. At the end of each trial, there was a silent period lasting between 5 to 10 s, randomly chosen from an exponential distribution (not to scale in Fig. 1A), preventing the mouse from anticipating when the next sound would be played. To trace the development of behavioral choice encoding over time, we initiated a week-long Pavlovian training period followed by a testing phase (Fig. 1C). During Pavlovian training, water rewards were provided at the end of the high-water period window (3 s after the sound) irrespective of the response timing, ensuring a reward for the mice regardless of when they licked. In this phase, mice got acquainted with their new environment, but there was no need to make a sound base decision. Mice could randomly lick and still get a reward. Despite this, mice started licking after the sound more often and less often in the pre-sound period (Fig. 1D two-way ANOVA for Bins 1–3, bin x period interaction F = 8.38, *p* = 7e^−04^ and for Bins 1–6 bin x period interaction F = 5.8, *p* = 0.0001), and the timing of their first lick post-sound neared 3 seconds, matching the water delivery timing (Appendix Fig. S1a, one-way Anova F = 14.46, *p* = 2.023e^−07^). Moreover, the miss rate went down (Fig. 1E, two-way ANOVA bin x type of trial interaction F = 16.24, *p* = 3e^−06^), suggesting a growing understanding that the water reward was contingent on the sound being played. After this initial phase, the mice progressed to the testing phase where the task rules changed, and correct timing of licking became crucial for obtaining a reward. During this testing phase, as mice got more experience with the new task’s rules, task performance improved (Fig. 1F, one-way ANOVA F = 4.14, *p* = 0.02).

Having a week of training followed by a change of rules, allowed us to categorize all behavioral and neural data into predefined bins representing distinct experience stages within the task. Importantly, it allowed us to examine if there were changes in the activity in the ACtx when the task rules changed. The first three bins constituted the training phase, while the last three bins constituted the test phase (Fig. 1C). Licks occurring before the auditory stimuli in the pre-sound period were deemed out-of-task licks with no consequences and no chance of obtaining a water reward.

While all licks following sound presentation could indicate tone detection, licks occurred more often in periods where licks were rewarded (Fig. 1G, two-way ANOVA, trial type: F = 55.5, *p* = 6.7e^−17^). The task’s structure ensured an adequate distribution of licks in the delay (EL trials), high water (Hit trials), and low water periods (LL trials), facilitating the exploration of diverse behavioral choices. Comparing Hit to LL trials allowed us to compare trials with similar motor responses, licking, but differentiated by different strategies. Active sound-based decision to get a large water reward in Hit trials versus a lick for a guaranteed small water reward in LL trials. Both Hits and EL trials indicated tone detection. Therefore, the comparison between Hit and EL trials provided insights into instances where the mice made a similar decision, even if prematurely executed in certain circumstances. By comparing pre-sound and post-sound licks, we were able to contrast similar motor and preparatory responses, which lack behavioral consequences in the former but exhibit behavioral outcomes in the latter. As mice gain experience with the task, we expect to find differences in the licking behavior and neural activity surrounding licks between the pre-sound period, where there are no behavioral repercussions, and the post-sound period, where licks result in either reward or timeout.

When we examined the lick dynamics, we observed differences in the lick patterns between the pre-and post-sound periods and the different behavioral outcomes (Hit, EL & LL). As the Bins advanced and animals gained experience with the task, there were notable changes in lick probability: the probability of the animals licking again after the first lick decreased, and the lick bursts became shorter (Fig. 1H). We found a significant difference in lick bursts’ length between train and test bins (Fig. 1H, two-way Anova Bin x time interaction F = 8.6, *p* = 3.7e^−08^). Lick bursts were shorter in Bins 4–6 compared to Bins 1–3 (post hoc *p* < 0.05 for all comparisons, Bonferroni corrected). This difference was particularly pronounced after Bin 1, both in the pre-and post-sound periods (Fig. 1H, two-way Anova Bin: F = 64.3 *p* = 1.1e^−66^; post hoc *p* < 0.01 for all comparisons between Bin 1 and all other Bins). Additionally, lick bursts in the post-sound period were generally longer than those in the pre-sound period (Fig. 1H, two-way Anova time: F = 109, *p* = 1.6e^−25^). This suggests that in the initial bins, lick timing was less critical, and mice were less familiar with the task rules and water delivery timing, resulting in longer lick bursts. As the mice gained experience, their lick bursts became shorter and more efficient.

We repeated this analysis using down-sampled lick data, reducing the sampling rate from 500 Hz to 30 Hz to match the neural data sampling rate. In both cases, we observed changes in lick patterns as the animal became more experienced with the task (Appendix Fig. S1b, lick burst length; two-way Anova F = 122, *p* = 1.9e^−28^, Bin x time interaction F = 7.3, *p* = 6.2e^−07^). We observed notable changes in lick probability and patterns, reflecting their growing experience with the task and the timing of water delivery. Especially, in Bin 1, when the mice were less experienced, they licked more indiscriminately. In contrast, by Bin 6 with increased experience, their licking became more efficient and targeted.

### Task-driven activity during behavioral choice evolves in the auditory cortex as the mice gain experience with the task

**Figure 2:**
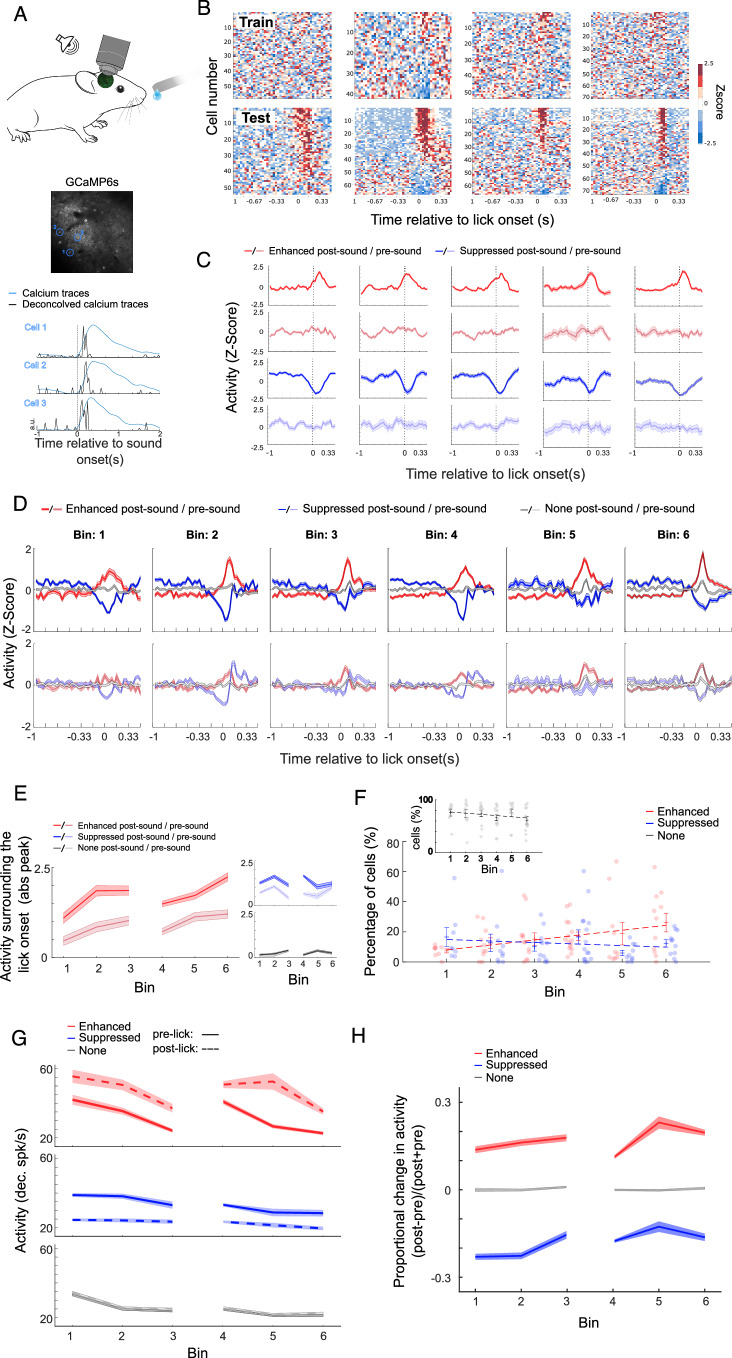
Task-driven activity during behavioral choice evolves in the auditory cortex as the mice gain experience with the task. (**A**) We performed chronic two-photon calcium imaging from the primary auditory cortex of awake, head-fixed mice that expressed GCaMP6s non-selectively in L2/3 neurons (*n* = 4335 Cells/*N* = 6 Mice) while mice gained experience with the behavioral task. All analysis was performed on the deconvolved calcium traces. Bottom: Example of calcium traces and deconvolved calcium activity traces for three ACtx cortical cells during white noise presentation at 70 dB SPL. (**B**) Maps of population activity of all cells (averaged across trials per cell) in training (session 3, bin 2) and testing (session 14, bin 6) for the same four mice. Time zero indicates the lick onset. (**C**) Example of the activity of the same ten cells in pre-sound (softer colors, bottom) and post-sound periods (darker colors, top). Time zero indicates the lick onset. Values represent mean ± se across trials. In many of the cells, the enhancement or suppression of activity starts before the lick. (**D**) Z-Scored activity surrounding the lick onset for: Enhanced-red, Suppressed-blue, and non-modulated-gray cells. Sessions were grouped by bin, shaded areas indicate se. Top: activity of cells classified in the post-sound period (licks with behavioral consequences – water or time out). Bottom: activity of the same cells during the pre-sound period (licks with no behavioral consequences). (**E**) Absolute peak of z-scored activity in the period surrounding the lick (from 165 ms before to 165 ms after the lick onset), per bin and cell group. Dark colors: activity in the post-sound period, Light colors: activity in the pre-sound period. Values represent mean ± se. Enhanced cells exhibited higher absolute peak activity during the post-sound period (two-way ANOVA, F = 127.7, *p* = 2.05e^−28^) and their activity increased as the mice gained experience with the task (post hoc, bin 1 vs bin 6 post-sound *p* < 0.001 Bonferroni corrected). (**F**) Percentage of cells divided by type and bin. The percentage of enhanced cells increased as the mice gained experience with the task. Points indicate individual sessions in each bin (pearson r = 0.9, *p* = 0.001 and two-way ANOVA, cell x bin interaction F = 10.4 *p* = 0.0093, post hoc bin 1 vs bin 6 *p* = 0.009 Bonferroni corrected). Values represent mean ± se across sessions. (**G**) Activity of enhanced, suppressed, and none cells before and after the lick (deconvolved spikes per second). The activity was calculated as the average activity during the 165 ms before the lick (solid line) and the average activity during the 165 ms following the lick (dash line). (**H**) Proportional change in activity per cell divided into enhanced, suppressed, and none. Values closer to zero mean no change in activity from the pre-lick to post-lick periods. Source data are available online for this figure.

When we examined the cortical activity surrounding the first lick, we noticed cells that increased or decreased their activity starting before the lick (Fig. 2A,B). To determine if this was an experienced-based change in activity, we examined the activity from the deconvolved calcium traces (Pachitariu et al 2016) surrounding the first lick in the post-sound period. We compared the activity during training, where mice were novices in the task (Fig. 2B top: example of a training session—session 3 bin 2), to the first lick during testing, where the mice had acquired more experience (Fig. 2B bottom: example of a testing session—session 14 bin 6). In the early training sessions, few units exhibited modulation in their activity surrounding the lick onset. However, a discernible shift occurred in later sessions, as mice gained experience with the task; several cells started to exhibit either enhanced or suppressed activity surrounding the lick (Fig. 2B, more examples in Appendix Fig. S2a). When we looked closer at the activity per cell, we found that the change in activity surrounding the lick was specific to licks in the post-sound period; the same cells did not exhibit a response to licks in the pre-sound period, where the licks had no behavioral consequence (Fig. 2C), suggesting task experience-dependent plasticity. These findings suggest a change in cortical activity specifically during post-sound licks. Crucially, this heightened activity does not manifest initially during the early stages of training but seems to evolve as mice progressively gain experience with the task and their new surroundings such as the presence of the lickspout or tones being played.

To further delineate the characteristics of cells influenced by behavioral choice and task experience, we categorized them into three groups—enhanced, suppressed, or non-modulated (Fig. 2D)—based on the activity surrounding the lick period after the sound (165 ms before to 165 ms after the lick onset compared to the same window of 330 ms earlier). This time frame was chosen to capture activity changes beginning before the lick (Clayton et al, 2021; Schneider et al, 2014), but without overlapping with any other stimulus. Pre-sound licks were used to dissect task experience-related modulation from predominantly motor-related signals. While we anticipated some activity modulation surrounding licks before the sound as mice familiarized themselves with their new environment, we expected it to be comparatively smaller due to the absence of sound information, lack of consequences for the licks, and no possibility of water reward during this period (Fig. 2D light colors).

When comparing activity in the pre-and post-sound periods, both enhanced and suppressed cells exhibited higher absolute peak activity surrounding the lick (Z-scored activity, 165 ms before to 165 ms after the lick onset) in the post-sound period (Fig. 2E, two-way ANOVA, F = 127.7 and 126.15, *p* = 2.05e^−28^ and 1.77e^−28^ accordingly). Interestingly, enhanced cell activity increased as the mice gained experience with the task and their environment (post hoc, bin 1 vs bin 6 and bin 1 vs bin 3 post-sound *p* < 0.001 and *p* = 0.01 Bonferroni corrected) while suppressed cells maintained higher absolute activity during post-sound periods without experience-related modulation (bin 1 vs bin 6 and bin 1 vs bin 3 post-sound *p* = 1 Bonferroni corrected). As expected, non-modulated cells showed no significant task or experience-related modulation (Fig. 2E, two-way ANOVA, F = 0.21, *p* = 0.96, bin 1 vs bin 6 post-sound *p* = 1). We hypothesized that the rise in peak activity might result from an increase in the percentage of cells active during the lick as the mice gained experience with the task. Indeed, as the mouse transitioned from novice to experienced, the proportion of cells that increased their activity surrounding the lick (165 ms before to 165 ms after the lick onset) increased significantly (Fig. 2F, Pearson r = 0.9, *p* = 0.001 and two-way ANOVA, cell x bin interaction F = 10.4 *p* < 0.0001, post hoc bin 1 vs bin 6 *p* < 0.01 Bonferroni corrected), while the proportion of cells that suppressed their activity remained unchanged (Fig. 2F, pearson r = −0.4, *p* = 0.355, post hoc bin 1 vs bin 6 *p* = 1 Bonferroni corrected). The heightened activity in enhanced cells, along with their increased prevalence in the population with task experience, suggests a potential role in encoding behavioral experience. To examine whether the distinct functional cell groups were spatially organized, we calculated the Euclidean distance between each pair of cells per cell group and found no significant difference (Appendix Fig. S2b, one-way ANOVA, F = 1.31, *p* = 0.27). Also, there was limited overlap between sound-responsive and enhanced or suppressed cells, with most enhanced or suppressed cells showing no modulation during sound presentation (Appendix Fig. S2c) and no special proximity to sound responsive cells (Appendix Fig. S2d).

Having identified distinct responses in various cell sub-populations, we examined with more detail the activity surrounding the lick period, categorizing it by cell type and distinguishing between activity before and after the lick (165 ms before and 165 ms after). A decrease in activity during both pre-lick and post-lick periods emerged as mice gained task experience (Fig. 2G). The more pronounced reduction in pre-lick activity (solid line) led to increased deltas of activity between the post and pre-lick periods for enhanced cells (Fig. 2H, two-way ANOVA, cell types x bin interaction F = 16.03, *p* = 1e^−28^, post hoc enhanced cells Bin 1 vs Bin 6 *p* = 0.01, Bonferroni corrected), but a reduction in activity difference for suppressed cells (Fig. 2H, post hoc Bin 1 vs Bin 6 suppressed *p* = 3e^−4^ Bonferroni corrected). This explains why, when examining the z-scored data in Fig. 2E, we observed an increase in peak activity: the larger difference in activity between the pre- and post-lick periods translated to higher peaks for enhanced cells when the activity was normalized per cell. The different way task experience modulates the activity of specific sub-populations in the ACtx could enhance the salience of choice-related information relayed to downstream areas to better inform behavioral decisions.

Interestingly, during the transition from training to testing, there was a resurgence to novice activity levels in enhanced cell activity (Fig. 2G,H). While, as anticipated, activity during the lick period was higher for enhanced cells and lower for suppressed cells in all bins, during the transition from training to testing (bin 3 to bin 4), there was an increase in both pre- and post-lick activity, specifically in enhanced cells. This rise in activity during both pre- and post-lick periods led to an overall increase in activity around the lick period. It also caused a decrease in the activity difference between pre-and post-lick periods in Bin 4, to values similar to baseline levels (Fig. 2H post hoc enhanced cells Bin 1 vs Bin 4 *p* = 0.9, Bonferroni corrected). This return to baseline values in Bin 4 explains the reduction in the lick-induced peak activity observed in the normalized data (Fig. 2E). The significant change in activity between Bin 3 and Bin 4 suggests an adaptation in response to the new task rules.

**Figure 3:**
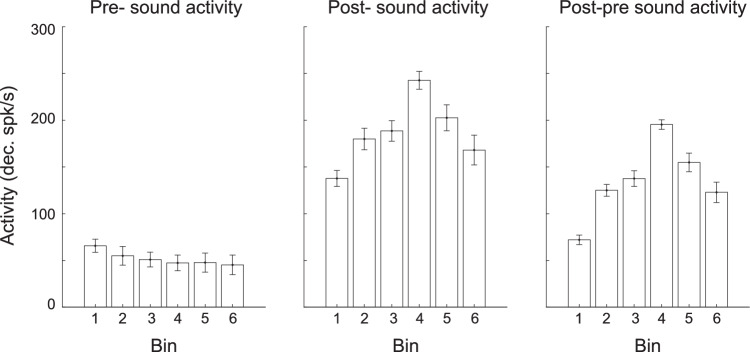
Experience-dependent changes in sound-evoked activity. Pre-sound and Post-sound activity of sound-responsive cells (*n* = 6 mice, responsive cells per bin = 143, 132, 128, 154, 162, and 148) were calculated during the half-second before or after the sound’s onset. Post-pre sound activity was the difference between the post- and pre-sound activity per cell. There was a significant change in the tone evoked activity (one-way Anova F = 211.8, *p* = 3.6e^−147^), characterized by an increase in activity during the first four bins, peaking at Bin 4 (post hoc, bin1 vs bins 3 and 4 *p* = 2.2e^−16^ and 6.4e^−18^, Bonferroni corrected), followed by a reduction in activity (post hoc, bin 4 vs bins 5 and 6 *p* = 1.4e^−16^ and 2.2e^−17^, Bonferroni corrected). Additionally, there was a small decrease in spontaneous activity as the bins progressed (one-way Anova F = 16.4, *p* = 1.7e^−15^). The bigger change in Post-sound activity compared to Pre-sound activity led to small changes when analyzing tone-evoked activity with baseline correction (one-way Anova F = 636, *p* = 3.9e^−286^). Values represent mean ± se. Source data are available online for this figure.

To explore if the reduction in activity was a global change in neural activity caused by learning and experience, we examined the activity surrounding the tone presentation. Here, we found a significant change in the tone-evoked activity (Fig. 3 middle, one-way Anova F = 211.8, *p* = 3.6e^−147^), characterized by an increase in activity during the first four bins, peaking at Bin 4 (post hoc, bin1 vs bins 3 and 4 *p* < 0.05, Bonferroni corrected), followed by a subsequent decrease in activity (post hoc, bin 4 vs bins 5 and 6 *p* < 0.05, Bonferroni corrected). Additionally, we observed a small decrease in spontaneous activity as the bins progressed (Fig. 3 left, one-way Anova F = 16.4, *p* = 1.7e^−15^). Interestingly, these changes in spontaneous activity didn’t seem to be driving the changes in tone-evoked activity across bins, as the same pattern of increased tone-evoked activity was observed even when accounting for pre-sound activity per cell (Fig. 3 right, one-way Anova F = 636, *p* = 3.9e^−286^). Therefore, while changes in spontaneous activity were present, they did not strongly influence changes in tone-evoked responses. In contrast, as we observed above, the greater reduction in pre-lick activity with increased task experience, did lead to a relative enhancement in neural responses following the lick.

To ascertain that the changes in activity surrounding the lick were driven by experience and task rule changes, we categorized cells also as enhanced, suppressed, or non-modulated based on their activity in the pre-sound period (Appendix Fig. S3a). During the pre-sound period there is information related to the environment the mouse is in, such as the presence of the lick spout or being head fixed, but there is no specific information about the sound and there is no chance of getting a water reward. Therefore, we expected to find lick-evoked activity (Clayton et al, 2021; Schneider et al, 2014; Zhou et al, 2014; Bigelow et al, 2019; Henschke et al, 2021; Vivaldo et al, 2023), but fewer task-dependent modulations. Indeed, while there was lick-dependent activity (Appendix Fig. S3a left) and an overall decrease in activity (Appendix Fig. S3b left), and a small dip in the proportional change for enhanced cells in Bin 4, there was no significant increase in the ratio between pre- and post-lick activity as mice gained experience in the task (Appendix Fig. S3b right, two-way ANOVA, cell types x bin interaction F = 2.2, *p* = 0.06 post hoc, enhance cells Bin 1 vs Bin 6 *p* = 1, Bonferroni corrected). As expected from this, experience had no significant effect on the peak activity of any of the cell types defined by the pre-sound activity (Appendix Fig. S3a right, normalized activity, two-way Anova Bin: F = 1.7 *p* = 0.1131; Bin 1 in all cell types vs bins 3 or 6 *p* < 0.05 for all post hoc Bonferroni corrected). The specificity of these changes to licks in the post-sound period implies that the alterations in activity cannot be accounted for only by the lick movement itself or the preparatory phase before the lick.

However, licking is not necessarily an all-or-nothing event; mice can exhibit a range of licking patterns, including prolonged bursts, brief episodes, and everything in between. Could different lick-bout dynamics in the pre-sound and post-sound periods be driving the changes we observed in neural activity? When we examined the lick probability in the 165 ms time window following the first lick (same window we used in our analysis above), we observed, as before, a general decrease in the lick probability following the first lick as mice gained experience with the task (Appendix Fig. S4a, two-way Anova, Bin: F = 142.9 *p* = 1.4e−148). This decrease appears to be primarily due to the reduced probability of licking after the first bin, as mice start to learn the rules of their new environment and understand when licking will result in water. Notably, lick probability in Bins 1 and 6 differed significantly from the other bins, with mice licking significantly more in Bin 1 and less in Bin 6 compared to the rest (post hoc *p* < 0.01 for all comparisons between Bin 1 and 6 and the remaining bins, Bonferroni corrected).

A decrease in lick activity between the pre- and post-sound periods could drive the changes in activity we found in enhanced cells. To test this hypothesis, we repeated the analysis examining changes in neural activity in lick-matched trials. For lick bursts starting with the first lick, we categorized the trials into three groups: short, intermediate, and long bursts. This categorization was performed twice—once using the down-sampled lick data for consistency with our neural data, and once using the original lick data for more precision and higher temporal resolution. A licking burst was defined as two or more consecutive licks with pauses greater than 500 ms (Boughter Jr et al, 2007; Johnson et al, 2010). The 500 ms threshold helped us to distinguish between bursts of licking (clusters of licks with short intervals between them) and pauses between bursts.

In all lick-burst-matched groups, we observed, consistent with our findings above, an increase in absolute peak activity around the lick in the post-sound period for both enhanced and suppressed cells and an increase in enhanced cell activity as the mice gained experience with the task (Appendix Fig. S4b, two-way ANOVA, short burst F = 41.43, *p* = 1.4e^−10^ and F = 29.62, *p* = 6.21e^−08^ accordingly, intermediate F = 45.3, *p* = 2.06e^−11^ and F = 28.1, *p* = 1.28e^−07^ accordingly, long F = 8.19, *p* = 0.004 and F = 9.01 *p* = 0.0027 accordingly & Appendix Fig. S4c Down-sampled data: short burst F = 31.16, *p* = 2.6e^−08^ and F = 11.9, *p* = 0.0006 accordingly, intermediate F = 32.7, *p* = 1.19e^−08^ and F = 30.9, *p* = 3.2e^−08^ accordingly, long F = 15.1, *p* = 0.0001 and F = 15.5, *p* = 0.0001 accordingly).

Consistent with Fig. 2G,H, we also found a decrease in activity during both pre-lick and post-lick periods across all lick-burst-matched groups as mice gained task experience (Appendix Fig. S5a). The more pronounced reduction in pre-lick activity (solid line) led to increased deltas of activity between the post and pre-lick periods for enhanced cells (Appendix Fig. S5b, two-way ANOVA, cell types x bin interaction short burst F = 11.21, *p* = 3.7e^−19^, intermediate F = 8.25, *p* = 2.2 e^−13^, long F = 3.76, *p* = 4.6e^−05^). Importantly, we also found here a resurgence to novice activity levels during the transition from training to testing.

Collectively, these results indicate that, as mice become more experienced with the behavioral task and the new environment, a sub-population of cells emerges, encoding the animals’ task-driven behavioral choices or expectations. This specific emerging cell ensemble modulates its activity in response to changing task demands, and it doesn’t seem to be driven by changing lick dynamics.

### Experience-dependent enhancement of noise correlations in auditory cortex sub-populations during behavioral choice

**Figure 4:**
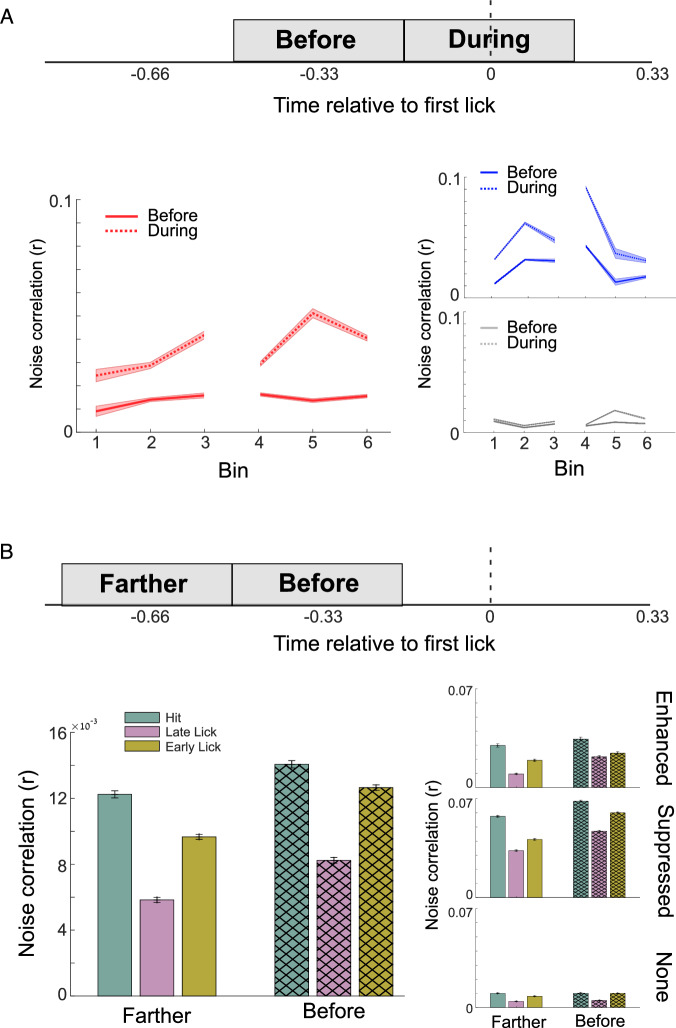
Experience-dependent enhancement of noise correlations in auditory cortex sub-populations during behavioral choice. (**A**) A comparison of noise correlations in neural activity across different cell types for the six mice participating in the behavioral task, both during and before the lick. “During”— a 330 ms window starting 165 ms before the lick, and “before”— a 330 ms window starting 495 ms before the lick. Enhanced and suppressed cells had higher noise correlations during the lick window compared to the window before the lick (two-way ANOVA, enhanced: F = 587.2, *p* = 4.5e^−127^, suppressed: F = 520.06, *p* = 3.8e^−14^), and enhanced cells showed a rise in noise correlations strength as the mice gained experience with the task (Bin 1 vs. Bin 6, *p* = 3.8e^−6^). (**B**) Left: Comparison of noise correlations in Hit, LL, and EL trials during two windows before the lick onset: “before”— the same window as above, and “farther”— a 330 ms window starting 825 ms before the lick. Noise correlations were higher in Hit trials and in the window closer to lick. Right: The same analysis was performed for each cell group. Values represent mean ± se. Source data are available online for this figure.

To establish the potential role of enhanced cells in encoding features of behavioral choice, it is crucial to assess if their elevated activity and greater representation in the population impact functional connectivity and information flow within the neural network as mice gain experience in the task. To test if this is the case, we calculated noise correlations as a measure of trial-to-trial co-variability of responses, providing an estimate of mutual connectivity and shared inputs between and within cell classes (Cohen and Kohn, 2011). We compared the noise correlations surrounding the first lick (Fig. 4A, 330 ms “during”, same window used in Fig. 2) and the period before (330 ms, “before”). We consistently observed higher noise correlations for enhanced and suppressed cell groups during the lick window (Fig. 4A, two-way ANOVA, enhanced: F = 587.2, *p* = 4.5e^−127^, suppressed: F = 520.06, *p* = 3.8e^−14^). This effect persisted when controlling for lick burst length (Appendix Fig. S6a, two-way ANOVA, enhanced cells short burst F = 217.7, *p* = 6.6e^−49^, intermediate F = 321, *p* = 3.6e^−71^, long F = 206, *p* = 5.2e^−58^, suppressed cells short burst F = 178, *p* = 1.6e^−40^, intermediate F = 197 *p* = 1.1e^−44^, long F = 144, *p* = 3.8e^−33^). This suggests a heightened level of functional connectivity or shared input among those neurons when making a behavioral choice. Notably, only enhanced cells showed an experience-related increase in noise correlations, with a significant rise in noise correlation strength from early to later sessions (Fig. 4A, Bin 1 vs. Bin 6, enhanced *p* = 3.8e^−6^, suppressed *p* = 1, non-modulated *p* = 1). This effect was much less pronounced when dividing the cells based on their pre-sound activity (Appendix Fig. S3c). This increase in noise correlations might reflect experience-driven circuit plasticity as the mice become more familiar with the task (Nassar et al, 2021). Interestingly, we also observed here a return to near baseline values during the transition from training to testing when task rules changed (Fig. 4A, bin 4).

While higher noise correlations during stimulus presentation limit the information capacity of a neural population, higher noise correlations during the decision period can enhance task performance by promoting information consistency and facilitating the conversion of sensory information into behavioral choices (Valente et al, 2021; Nassar et al, 2021). Thus, we anticipated higher noise correlations surrounding the lick period, when mice made the correct rather than incorrect behavioral choices, similar to higher noise correlations found in association areas when the mice executed correct behavioral choices (Valente et al, 2021). If noise correlations are indicative of a behavioral choice, we would also expect them to increase as the animal approaches a decision. To test these two predictions, we compared noise correlations’ strength in Hit, LL, and EL trials (Fig. 4B left) during the before window and a 330 ms window “farther” from the lick. We found that noise correlations were higher in Hit trials than EL and LL trials in both time windows (Fig. 4B left, two-way ANOVA, condition: F = 577.3, *p* = 3.5e^−251^, post hoc Hit vs LL (farther) *p* = 4.3e^−22^, Hit vs LL (before) *p* = 7.8e^−7^, Bonferroni corrected). The effect persisted when repeating the analysis in lick burst matched trials (Appendix Fig. S6b). We also found higher noise correlations in the window “before” the lick than the window “farther” from the lick (Fig. 3b, two-way ANOVA, time window- F = 256.66, *p* = 9.44e^−58^, time window x condition interaction F = 5, *p* = 0.006, post hoc Hit farther vs Hit before *p* < 0.0001 Bonferroni corrected). These results support previous findings showing higher noise correlations during decision periods (Nassar et al, 2021; Valente et al, 2021) and suggest a role for enhanced and suppressed cells in this process.

When performing the same analysis per cell group, we found that during the post-sound period, the noise correlation increased in the transition between farther to before time points for enhanced and suppressed cells but not for non-modulated cells (Fig. 4B right, two-way ANOVA, post hoc Hit “farther” vs. “before”, Enhanced *p* = 0.013, Suppressed *p* = 4.8e^−37^, non-modulated *p* = 1). Collectively, these results suggest a strengthening of coupling among enhanced and suppressed cell ensembles that starts before the lick. Correlations between enhanced cells also increased across behavioral sessions, suggesting specific experience-related circuit plasticity (Komiyama et al, 2010).

### Task experience improves discriminability between behavioral choices by specific populations in the auditory cortex

Next, we investigated how experience-related changes in both single-cell and pairwise activity impacted the ability of neuronal populations to differentiate between behavioral choices, such as licking with the prospect of a substantial water reward (Hit) versus licking for a small amount of guaranteed water (LL). To this end, we investigated the trajectories of neural population activity across trials. Neural trajectories are a simple way to express the network state of multi-neuronal data. Similar trajectories with small Euclidian distances between Hit and LL trials would suggest no difference in the neural representation between the conditions while differing trajectories would indicate a difference in the population activity structure (Churchland et al, 2012; Allsop et al, 2018; Asokan et al, 2023).

**Figure 5:**
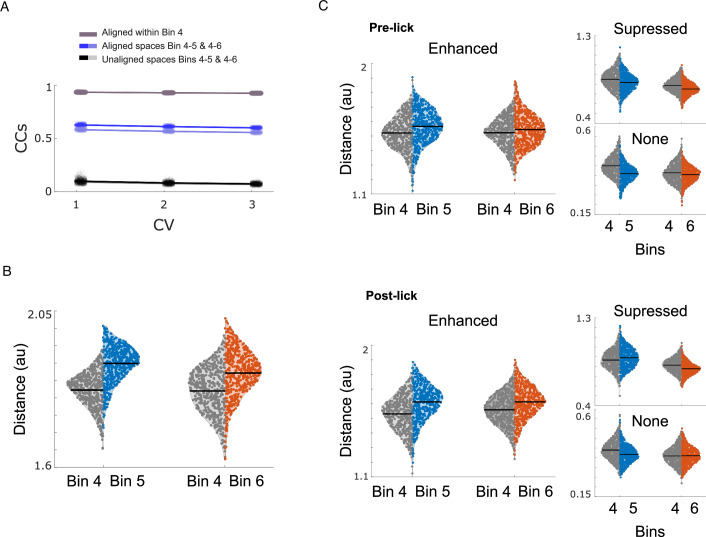
Task experience improves discriminability between behavioral choices by specific populations in the auditory cortex. (**A**) Canonical correlations for the three main canonical variables for alignment of Bins 4 & 5 and Bins 4 & 6, upper bound calculated from within-day variability in the latent dynamics in Bin 4, and the lower bound calculated by the unaligned spaces. The aligned spaces maintained a higher correlation across days than the unaligned spaces. The analyses were conducted using neural activity data from the six mice that participated in the behavioral task. (**B**) Comparison of Euclidian distance between Hit and LL trials in Bins 4 & 5 and Bins 4 & 6 in the aligned spaces. (**C**) Comparison of Euclidian distance between Hit and LL trials in Bins 4 & 5 and Bins 4 & 6 in the aligned spaces for the different cell types in the pre-lick (top) and post-lick (bottom) periods. Source data are available online for this figure.

Before calculating the Euclidian distances, we used canonical correlation analysis (CCA) to “align” the latent dynamics across the different Bins (Dabagia et al, 2023; Veuthey et al, 2020; Gallego et al, 2020, 2018). We found the linear transformations that make the latent dynamics from Bin 5 and 6 maximally correlated to those from Bin 4 and projected them back to the original data. These transformations should compensate for the changes in the recorded population of neurons caused by turnover. The three leading canonical correlations (CCs) were relatively high (Fig. 5A), demonstrating the preservation of significant components of the neural mode dynamics across bins (see methods for more details).

Examining the Euclidean distance between Hit and LL trajectories revealed an increased distance in Bins 5 and 6 when compared to Bin 4 (Fig. 5B, t-test *p* = 7.3e^−99^ and 5.1e^−33^ accordingly and permutation test *p* < 0.01 for both). We observed the same phenomenon when comparing Euclidean distances between Hit and LL trajectories in lick-matched trials (Appendix Fig. S7a, t-test Bin 4 vs Bin 5 *p* = 1.4e^−104^ and Bin 4 vs Bin 6 *p* = 5e^−74^). To determine whether this increased discriminability was due to the presence of the water reward—since the lick results in water consumption in LL trials but not in Hit trials—we repeated the analysis, dividing activity into pre- and post-lick periods. As expected, discriminability increased during the post-lick period; however, we also found heightened discriminability even before the lick occurred (Appendix Fig. S7b, t-test Bin 4 vs Bin 5/6 pre and post *p* < 0.001 for all comparisons).

To investigate the role of enhanced cells in this process, we tested whether cell-specific population activity could also discriminate between conditions (Fig. 5C). We examined the Euclidean distances between Hit and LL trials, categorizing them by cell type and distinguishing between pre- and post-lick periods. Examining the Euclidean distances, we found an increase in distances between Hit and LL trials for enhanced cells both in the pre-and post-lick periods when comparing Bin 4 to Bins 5 and 6 (Fig. 5C, one-sided t-test enhanced cells: pre-lick *p* = 7.5e^−10^ & *p* = 6.7e^−04^, post-lick: *p* = 5.7e^−34^ & *p* = 2.1e^−38^ Bin 4 vs 5 and Bin 4 vs 6 accordingly). We also found an increase in distance for suppressed cells, but only in the post-lick period between Bins 4 and 5 (pre-lick *p* = 1 & *p* = 1, post-lick: *p* = 9e^−12^ & *p* = 0.9 Bin 4 vs 5 and Bin 4 vs 6 accordingly) and no increase in distance for none cells (pre-lick *p* = 1 & *p* = 0.99, post-lick: *p* = 1 & *p* = 0.73 Bin 4 vs 5 and Bin 4 vs 6 accordingly). These findings suggest that as mice become more experienced with the task, the enhanced cell population shows an improved ability to discriminate between Hit and LL trials both before and after the lick.

These findings suggest that the enhanced population becomes increasingly adept at distinguishing between Hit and LL trials as mice become more experienced with the task. If the enhanced population activity indeed discerns between conditions involving licks with the potential for a significant water reward and licks for a small amount of certain water, we would expect the population activity to also differentiate between EL and LL trials. EL trials represent instances where a lick is made without the certainty of obtaining water, even if the lick was premature. We tested the discriminability between EL and LL trials, and indeed, we found higher discriminability in enhanced cells during the post-sound period when compared to the pre-sound period (Appendix Fig. S7c, t-test for Bin 4 vs 5 and Bin 4 vs 6, both *p* < 0.01). Together these findings suggest that the activity of the enhanced sub-population can distinguish between states where the mouse licks in order to get a large water reward and instances of licking for a guaranteed small water reward.

### The enhanced cell population in the auditory cortex can accurately discriminate between task phases

**Figure 6:**
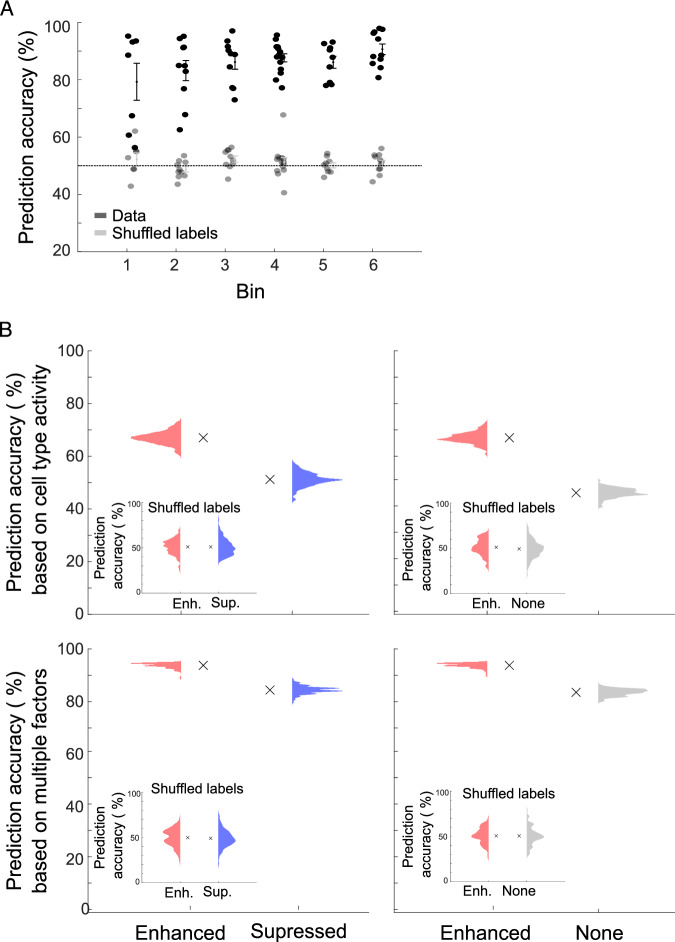
The enhanced cell population in the auditory cortex can accurately discriminate between the task’s phases. (**A**) Classification of licks to the pre-sound or post-sound period, based on the activity of all cell types (black, mean ± se across sessions, *n* = 66 sessions *N* = 6 mice). Gray: The decoder’s prediction accuracy dropped to chance levels when labels were shuffled. Prediction accuracy increased when comparing novice and experienced stages (two-way ANOVA, F = 675.7, *p* = 3.69e^−48^, post hoc Bin 1 vs. Bin 6, *p* = 0.006). (**B**) Top: Prediction accuracy by cell type activity. X marker represents the mean prediction accuracy. Inset — same analysis with shuffled labels. Enhanced cells exhibited higher prediction accuracy than suppressed and non-modulated cells (Permutation tests, *p* = 2e^−4^ and *p* = 2e^−4^). Bottom: incorporating additional behavioral measures, such as lick probability and the proportion of modulated cells, enhanced the overall prediction accuracy. Inset — same analysis with shuffled labels. Source data are available online for this figure.

Our analysis of enhanced cells’ activity consistently found task-specific modulations, specifically in the post-sound period. These observations led us to hypothesize that the activity of this sub-population would be enough to decode whether the mouse was licking in the pre- or post-sound phases of the task. To test this hypothesis, we used a neural decoder to determine if the observed licks could be accurately classified as occurring during the pre-sound or post-sound period solely by the activity in the ACtx. We first reduced the dimensionality of the ensemble data matrix with principal component analysis and then used a support vector machine on the principal component projections to classify whether the mouse was licking during the pre-sound or post-sound period. The general population neural data exhibited high prediction accuracy for distinguishing licks in different task phases when compared with data with shuffled labels (Fig. 6A, two-way ANOVA, F = 675.7, *p* = 3.69e^−48^). As expected, the decoder’s prediction accuracy increased when comparing novice and experienced stages (Fig. 6A, post hoc Bin 1 vs. Bin 6, *p* = 0.006).

We next examined which cell group could better discriminate between the task phases, with a particular focus on the enhanced cells based on our earlier findings. Indeed, the enhanced cell ensemble exhibited higher prediction accuracy than suppressed (Fig. 6B top left, Permutation tests, *p* = 2e^−4^, inset: same analysis with shuffled labels) and non-modulated cell groups (Fig. 6B top right, Permutation tests, *p* = 2e^−4^, inset: same analysis with shuffled labels).

Incorporating additional behavioral measures such as lick probability (Fig. 1D) and the proportion of modulated cells (Fig. 2F) improved the overall prediction accuracy. Even after incorporating these measures, the enhanced cell group maintained higher prediction accuracy than the suppressed and non-modulated cell groups (Fig. 6B bottom, Inset: same analysis with shuffled labels).

To test if the changes in lick probability between pre- and post-sound periods drove these findings, we repeated the analysis in match-lick trials. Similarly to our previous results, we found that the enhanced cell group had higher prediction accuracy when testing all groups (Appendix Fig. S8, long, short & intermediate lick bursts, Permutation tests, *p* < 0.0001), suggesting that changes in lick dynamics are not driving the phenomenon. As another control, we repeated the analysis after dividing the cells based on their pre-sound activity. We didn’t find a significant difference in the prediction accuracy between enhanced and suppressed or none cells (Appendix Fig. S3d), suggesting that while there is lick-induced activity in the pre-sound period, the effect of experience seems to be gated by the sound.

These findings underscore that the enhanced cell population can reliably predict whether the mouse is licking before or after the sound. This reaffirms our previous results, demonstrating that the enhanced cell population encodes not only motor signals but also aspects of behavioral choice.

## Discussion

We used two-photon calcium imaging and a reward-driven licking task with a delayed period (Fig. 1) to capture the activity of numerous cells in the ACtx of mice making behavioral decisions. Following the activity of the ACtx for more than two weeks of training and testing revealed a progressive increase in choice-dependent activity within a specific subpopulation of neurons as mice became experienced with the task. Groups of primarily non-sensory cells, initially mainly unresponsive at decision times, underwent task-specific alterations in their activity, particularly in activity starting before licks following the sound stimulus (Figs. 2–4). These changes aligned with the mice’s growing familiarity with the task, its environment, and the changes to the task rules. We categorized cells based on the type of modulations of activity they exhibited surrounding the lick: enhanced, suppressed, or non-modulated (Fig. 2). A closer examination revealed distinct dynamics in the modulated cells, especially in the activity of enhanced cells, which exhibited changes as the mice gained experience with the task. Our results indicate a dynamic restructuring of population activity in the ACtx to encode features of the decision-making process that develop over time with experience.

While both enhanced and suppressed cells exhibited activity related to the task, we observed distinctive changes, particularly in enhanced cells, encompassing alterations in the size of the representational ensemble, the magnitude of response, and the relationship between activity preceding and following the lick as mice gained experience with the task (Fig. 2). These patterns align with prior observations in the visual (Guo et al, 2014) and parietal cortex of expert mice (Goard et al, 2016), where suppressed neurons played less of a role in encoding task-relevant variables. Without specifically manipulating the activity of the suppressed neurons, it is difficult to determine what role they may play in the behavioral choice. One possibility could be that the suppression during the behavioral choice serves to diminish ongoing, task-irrelevant activity. This could amplify the readout of task-relevant activity by the enhanced cell population by higher-order regions. Interestingly, the changes in neural and behavioral activity began during the training phase, when sound detection was not required for obtaining a water reward, allowing alternative behavioral strategies. By Bin 2, we noted an increase in licking frequency post-sound, a reduction in lick burst length, and significant differences in neural activity surrounding licks between pre- and post-sound periods. The concurrent increase in the percentage of cells responsive during the lick period, coupled with an increase in activity as the mice gain experience with the task, indicates that the representation of features of the decision-making process in the ACtx is not innate but develops early and continues to be refined with experience by recruiting cortical cells to encode the behavioral choice. The dynamic recruitment of cortical cells suggests a reorganization of top-down signals to the ACtx, potentially facilitating the transmission of the decision’s significance upstream.

We utilized noise correlations as a metric for assessing functional connectivity and information flow within the network (Fig. 4). Our observations revealed heightened noise correlations during post-lick periods, with task-dependent variations, consistent with our earlier findings, particularly evident in enhanced cells. The noise correlations in enhanced cells increased progressively as mice gained experience with the task, with a notable decrease in Bin 4 as the animal transitioned from training to testing. The observed increase in noise correlations as mice approached a decision and the stronger correlations in correct trials provide additional evidence supporting the notion that enhanced cells contribute to the encoding of the diverse behavioral choices.

Our analysis of population activity revealed that the activity of the enhanced ensemble, as opposed to the suppressed and non-responsive ensembles (Fig. 6), was sufficient to decode whether a mouse licked in the pre- or post-sound stage of the task. Interestingly, the decoding accuracy of this small ensemble improves as the mice gain experience with the task, suggesting a circuit reorganization driven by experience.

Our analysis of population activity also uncovered a progressive improvement in differentiating between Hit trials, where mice had the potential for a substantial water reward, and LL trials, where mice were guaranteed a minor reward. This refinement in trial-type discrimination was also primarily driven by the varied activity trajectories exhibited by the ensemble of enhanced cells across the diverse behavioral choices (Fig. 5). Additionally, these ensembles demonstrated the ability to discriminate between EL and LL trials, suggesting that their population activity can effectively differentiate between trials involving different types of behavioral choices.

The population activity of enhanced cells may represent distinct choice types (e.g., taking a risk vs. playing it safe) or varying reward expectations (large vs. small water reward). However, disentangling decision-making from reward anticipation in our behavioral paradigm is challenging. Prior work has shown that the activity in the ACtx of expert mice (Jaramillo and Zador, 2011) or expert non-human primates (Brosch et al, 2011) can indeed reflect reward expectations. Yet, these studies often struggled to separate activity linked to specific movements from activity dependent on perceptual choices, as they compared periods of movement with periods of quiet waiting. To address this issue and delineate which part of the activity modulation is due to perceptual choice versus primarily motor-related activity (Clayton et al, 2021; Schneider et al, 2014, 2018), we compared lick-triggered activity during two distinct periods: the pre-sound period, where licks had no chance of eliciting a water reward, and the post-sound period, where the lick could trigger a reward. Although lick-triggered activity was observed in the pre-sound period, consistent with previous findings in ACtx (Clayton et al, 2021; Schneider et al, 2014; Zhou et al, 2014; Bigelow et al, 2019; Henschke et al, 2021; Vivaldo et al, 2023), the modulation of activity, correlations, and condition coding seem to be gated by the sound.

Since, as expected from the learning process and increased efficiency in task performance, we observed changes in lick dynamics during the different bins and trial outcomes, we further explore whether the effects we found in neural activity were driven by changes in lick dynamics or if motor signals that represent a behavioral choice in the auditory cortex were being gated by their auditory task relevance. To distinguish between these possibilities, we repeated all our main analyses in lick-burst matched trials and tested if changes in lick dynamics could explain our findings. We found that this wasn’t the case and that the alterations in activity in the ACtx—whether they are driven by choice or reward prediction—are not solely attributable to changes in lick dynamics. Instead, they indicate the presence of an experience-specific mechanism influencing motor signals in the ACtx.

An intriguing pattern that consistently emerged in our data is the resurgence of activity (Fig. 2) and noise correlations (Fig. 4) to levels near baseline during the transition from training to testing. This shift signifies a change in rules, moving from the assured receipt of water to the requirement of actively licking within the correct time window to secure the reward. The observed rebound in activity during this transition robustly supports our conclusion that the activity in the auditory cortex is not exclusively driven by motor functions but is intricately tied to task demands, expectations, and the gained experience. This is evident in the changes in the ACtx activity to alterations in task rules, despite the constancy of the motor output, i.e., licking. Importantly, this rebound phenomenon is notably driven by changes in the activity of enhanced cells (Fig. 2), implying a distinctive role for them in behavioral decision-making and potentially representing the certainty of the decision.

The temporal separation between sensory perception and the behavioral expression of the animal’s choice in the task allowed us to analyze distinct behavioral components. However, it is important to consider that in delayed-response tasks, the subject may reach its decision at various time points: immediately after hearing the sound, during the delay period, or just prior to the lick. enabling us to examine how cortical activity is modulated during the behavioral output phase. Consequently, our emphasis stayed on the activity surrounding the behavioral output of the decision—specifically, the lick onset. At this juncture, the animal has already made its decision, enabling us to examine how cortical activity is modulated during the behavioral output phase.

Our collective findings highlight experience-dependent modifications in the ACtx, encompassing neural activity, functional connectivity, and information representation, particularly within the enhanced cell population. We show that the representation of the diverse behavioral choices develops with time and experience and is modulated by changes in task rules. Furthermore, we establish that the alterations in activity in the ACtx could not be solely attributed to changes in lick dynamics; rather, they reflect the behavioral significance of the decision and motor action. The specificity of the alterations to licks in the post-sound period and the evolving nature of the changes in activity underscore the ACtx’s remarkable adaptability to represent more than sensory information. This adaptability enables the development and modulation of non-sensory, decision-related signals, especially in response to changing task demands.

## Methods

**Table:** Reagents and tools table

Reagent/Resource	Reference or Source	Identifier or Catalog Number
**Experimental Models**		
Mice PV-Cre x Ai14	Jackson Laboratory	Cross between stock no: 017320 and 024109
**Recombinant DNA**		
N/A		
**Antibodies**		
N/A		
**Oligonucleotides and other sequence-based reagents**		
N/A		
**Chemicals, Enzymes and other reagents**		
N/A		
**Software**		
MATLAB 2023	MATLAB	N/A
**Other**		
AAV5.Syn.GCaMP6s.WPRE.SV40	Addgene	100843-AAV5
Bergamo III Multiphoton Microscopes	Thorlabs	N/A
Mai Tai Laser	Spectra-Physics	N/A

### Experimental model and subject details

All procedures were approved by the Ben-Gurion University animal care and use. Data were collected from 6 adult mice (8–16 weeks postnatal; PV-Cre x Ai14, JAX stock no: 017320 and 024109, respectively). Mice of both sexes were used for this study. Mice were maintained on a reverse 12 h light/12 h dark cycle and were provided with ad libitum access to food and water unless they were undergoing behavioral testing, in which case they had restricted access to water in the home cage.

### Survival surgeries for awake, head-fixed imaging, and behavior experiments

Mice were anesthetized with isoflurane in oxygen (5% induction, 1.5% maintenance). The dorsal surface of the mice’s heads was trimmed and sterilized. ThermoStar homeothermic blanket monitoring system was used to maintain body temperature at 36.6 °C (RWD). Lidocaine hydrochloride was administered subcutaneously to numb the scalp. The dorsal surface of the scalp was reduced using surgical scissors, and the periosteum was removed. The skull surface was prepped with an etchant (C&B metabond) and vetbond (3 M) before affixing a custom stainless-steel headplate to the dorsal surface with dental cement (C&B metabond). At the conclusion of the headplate attachment and any additional procedures listed below, Buprenex (0.05 mg/kg) and meloxicam (0.1 mg/kg) were administered, and the animal was transferred to a warmed recovery chamber.

### Virus mediated gene-delivery

For mice used in imaging experiments, two burr holes were made in the skull over the auditory cortex (1.75–2.25 mm rostral to the lambdoid suture). A precision injection system (Nanoject III) was used to inject 75 nL of AAV5.Syn.GCaMP6s.WPRE.SV40 in each burr hole 180–230 mm below the pial surface. Before starting the imaging sessions, we waited ~3 weeks of virus incubation.

### Two-photon calcium imaging

Three round glass coverslips (one 4 mm, two 3 mm, #1 thickness) were etched with piranha solution and bonded into a vertical stack using transparent, UV-cured adhesive. Headplate attachment, anesthesia and analgesia follow the procedure described above. A 3 mm craniotomy was made over the right ACtx using a scalpel and the coverslip stack was cemented into the craniotomy. An initial widefield epifluorescence imaging session was performed to visualize the tonotopic gradients of the auditory cortex and identify the position of A1 as described previously (Romero et al, 2020). Two-photon excitation was provided by a Ti:Sapphire-pulsed laser tuned to 940 nm. Imaging was performed with a 16 X/0.8NA water-immersion objective (Nikon) from a 512 × 512 pixel field of view at 30 Hz with a Galvo-Resonant 8 kHz scanning microscope (Thorlabs). Scanning software was synchronized to the stimulus generation hardware using digital pulse trains. The microscope was rotated 50–60 degrees off the vertical axis to obtain images from the lateral aspect of the mouse cortex while the animal was maintained in an upright head position. Imaging was performed in a light-tight, sound-attenuating chamber mounted on a floating table. Animals were monitored throughout the experiment to confirm that all imaging was performed in the awake condition. Imaging was performed in layers L2/3, 180–220 mm below the pial surface. Each session we returned to the same area, guided by the blood vasculature, but not necessarily the same cells. Fluorescence images were captured at 2x digital zoom, providing an imaging field of (0.42 × 0.42 mm). Raw calcium movies were processed using Suite2P (Pachitariu et al, 2016), a publicly available two-photon calcium imaging analysis pipeline. Spike deconvolution was also performed in Suite2P (Pachitariu et al, 2016), using the default method based on the OASIS algorithm (Pachitariu et al, 2018; Stringer and Pachitariu, 2019). All the following analyses were performed in the deconvolved activity. Before starting the behavioral sessions, we checked for sound responsiveness of the neurons using white noise generated from a Gaussian distribution at different sound levels (15–70 dB SPL with 5 dB SPL steps) with a 3.5 trial duration.

### Behavioral task

Animals were weighed and placed on a water restriction schedule (at least 1 mL per day). During behavioral training and testing, animals were weighed daily to ensure they remained above 80% of their initial weight and examined for signs of dehydration, such as fur tenting. Mice were given supplemental water if they received less than 1 mL during a session or appeared excessively dehydrated. Mice performed the task in the dark while imaging was performed at the same time. Before starting the imaging/behavior sessions, the mice were acclimated to being head-fixed for three days (the waterspout was presented on the third day).

The task consisted of two phases: a training phase and a testing phase. During the training phase, a 6 kHz or a 16 kHz pure tone at a constant level of 60 dB was presented at random and followed by a reward of sweetened water delivery (1%) after a 3-s delay, irrespective of the mice’s licking behavior. After a week of training the mice moved to the testing phase. In the testing phase mice were rewarded based on their performance. Mice had to delay their decision (lick) for 1.5 s. Following this delay window, they had 1.5 s to express their choice by licking the lickspout. Correctly timed licks (Hit) resulted in a big dose of sweetened water (12 μL) a second later. Conversely, an early lick (EL) during the delay response window led to a timeout without water. The mice could wait until the end of the high-water period without licking and a small water droplet (4 μL) would be dispensed (late lick period – LL, also 1.5 s). Licking in catch trials where no sound was presented was counted as a False Alarm and refraining from licking during the trials where the sound was presented was counted as a miss. At the end of each trial, there was a period of silence (5–10 s randomly chosen from an exponential distribution), so the mouse would be able to anticipate when the sound would be played. In Fig. 1D we compared the percentage of trials with licks for the same time windows (4.7 s). To do so, we added to the pre-sound period 3.7 s from the previous trial quiet period. For Fig. 1F, d’ was calculated as d’ = z(H)−z(F). Where H is the hit rate and F is the false alarm rate calculated from the catch trials. In Fig. 1E,G, the analysis was done in the post-sound period.

## Data analysis

### Two-photon calcium imaging

Neural data was aligned with behavioral task events using MATLAB (Mathworks) scripts. Behavioral data was down-sampled from 500 to 30 Hz to match the neural data sampling rate. Cells with firing rates lower than 1 sp/s were removed from further analysis. For the analysis of behavioral choice, we analyzed the activity of licks that occurred after a lapse of at least 330 ms without any other licks or stimulus, aiming to isolate activity directly associated with the individual lick event. Per session, a matrix of Cells X Frames X Trials was obtained for further analysis. We used trials where the mouse licked for further analyses.

We examined the spatial distance between cells by calculating the Euclidean distance of each pair in the Enhanced, Suppressed, and None cell groups per session and mouse. Then, we averaged across sessions. We repeated this analysis between sound-responsive cells and Enhanced, Suppressed, and None cell groups.

### Cell categorization

Cells were classified as enhanced, suppressed, and none-modulated based on the comparison of the activity surrounding the lick period (165 ms before to 165 ms after the lick onset compared to the same window of 330 ms beforehand) using a non-parametric statistical test; Wilcoxon signed-rank test. We chose this time window to capture changes in activity starting before the lick (Clayton et al, 2021; Schneider et al, 2014), but without overlapping with any other stimulus. We found no difference between the response surrounding the lick after the 6 kHz or a 16 kHz tone trials; therefore, we combined all the data for subsequent analysis. For Appendix Fig. S3, the cells were classified using the same activity window in the pre-sound period. Cells were classified as sound responsive based on the comparison of the activity 500 ms before and 500 ms after the sound onset across trials.

We used each cell’s x and y locations per imaging session to calculate the Euclidean distance. We calculated the Euclidean distance of each pair of cells in the Enhanced, Suppressed, and None cell groups or per sound-responsive cell and Enhanced, Suppressed, and None cell per session. Then, we averaged across sessions.

### Lick-burst length

We categorized the trials into three groups: short, intermediate, and long lick bursts. This categorization was performed twice—once using the down-sampled lick data for consistency with our neural data, and once using the original lick data for more precision and higher temporal resolution. A licking burst was defined as two or more consecutive licks with pauses greater than 500 ms (Boughter Jr et al, 2007; Johnson et al, 2010). The 500 ms threshold helped us to distinguish between bursts of licking (clusters of licks with short intervals between them) and pauses between bursts. We categorized lick bursts based on their duration as follows: short lick bursts (0.002 ≤ x < 0.15 s), intermediate lick bursts (0.15 ≤ x < 0.5 s), and long lick bursts (0.5 ≤ x < 1 s). For the down-sampled lick data, the categories were adjusted to short lick bursts (0.033 ≤ x < 0.2 s), intermediate lick bursts (0.2 ≤ x < 0.5 s), and long lick bursts (0.5 ≤ x < 1.33 s). Bursts longer than 1 s (or 1.33 s for the down-sampled data) were excluded from this analysis due to insufficient trial numbers across all bin-trial outcome combinations.

### Noise correlations

We quantified noise correlations as the Pearson’s correlation coefficient between normalized activity of the entire cell ensemble, or per cell type, per session with the ‘Corrcoef ’ function in MATLAB and aggregated the outputs per bin. Pair-wise noise correlations of windows of 0.33 s were compared at different windows during the lick-triggered activity. If there was no activity at all through the entire window, the trial was discarded. From the entire noise correlations matrix, values were averaged over conditions or cell groups according to the analysis.

### Canonical correlations and Eucledian distances

As a first step, we use CCA to align the neural spaces from Bins 5 & 6 to Bin 4. The method systematically finds new directions within each neural space such that the corresponding one-dimensional projected activities are maximally correlated. For this analysis, we included all the concatenated trials for each of the Bins. We first equalized the number of Hit and Late Lick trials within the corresponding session to assemble these data matrices. CCA models were calculated using the MATLAB function canoncorr.

We used the within-day variability in the latent dynamics across blocks of trials in Bin 4 to obtain an upper bound for the across-day CCs. We split all the trials in one day into two nonoverlapping sets of trials, ensuring that the groups were matched by types of trials, and performed CCA on the latent dynamics (500 repetitions). To set the lower bound we computed the pairwise correlations between unaligned spaces.

Next, we calculated the Euclidean distance between the different trial types (Hit vs LL and EL vs LL) in the aligned spaces. This approach allowed us to compare the distances between the trials in Bin 4 vs Bin 5 and Bin 4 vs Bin 6.

### Support vector machine classifier

To determine if the ensemble activity could decode whether the lick occurred during the pre-sound or post-sound periods, we used a support vector machine classifier (SVM) with a non-linear radial basis function (RBF) kernel. We fitted the classifier model to a data matrix of cell activity. For lick phase classification using the entire cell population, the data matrix consisted of the mean activity rate within a 330 ms period, starting 165 ms before the lick per trial and phase. Ensemble analyses included all identified neurons in any given field of view. Because there were fewer licks during the pre-sound phase (because the mice learned the task rules) and to not bias the classifier towards the majority class, we used an over-sampling technique that balances class distribution by synthetically generating new minority class instances along directions from existing minority class instances towards their nearest neighbors (SMOTE) (SMOTE: Synthetic Minority Over-sampling Technique|Journal of Artificial Intelligence Research). To reduce the influence of any possible inequities in sample sizes across mice or conditions, and to avoid overestimation and an unstable result resulting from the larger number of features than the number of samples, we used principal components analysis to reduce the dimensionality of the data matrix before classification. We ran the SVM on the principal components that explained 80% of the variance. 10-fold cross-validation was then used to train the classifier and compute a misclassification rate. This process was then iterated according to the number of sessions. As a control, we repeated this analysis with shuffled labels. The SVM training and cross-validation procedure was carried out in MATLAB using the ‘fitcsvm’, ‘crossval’, and ‘kfoldLoss’ functions. To classify the licks by the activity of each cell group separately, we used the mean trial activity per cell group to create a decoder across sessions. We calculated the decoding accuracy 100 times, using the ‘kfoldLoss’ function to create the distribution for each cell group. In Fig. 5. Bottom, we added to the decoder information about behavioral measures per session: the percentage of trials with licks (as presented in Fig. 1D) and the percentage of modulated cells (as presented Fig. 2F).

### Statistical analysis

All statistical analyses were performed in MATLAB R2023a (Mathworks). Data shown in all analyses is the mean activity ± SEM unless otherwise indicated. Post hoc pairwise comparisons were corrected for multiple comparisons using the Bonferroni correction. Blinding was not applicable.

## Supplementary information

Appendix

Peer Review File

Source data Fig. 1

Source data Fig. 2

Source data Fig. 3

Source data Fig. 4

Source data Fig. 5

Source data Fig. 6

## Data Availability

The source data of this paper is collected in the following free access database: BioStudies, accession number S-BSST1639: https://www.ebi.ac.uk/biostudies/studies/S-BSST1639.
