# Supplementary material for: Experience-driven development of decision-related representations in the auditory cortex
Source: EMBO Rep. 2024 Nov 11;26(1):84–100. doi: 10.1038/s44319-024-00309-0 (PMC11723978; doi:10.1038/s44319-024-00309-0)
Supplement: Supplementary file 1 — Appendix [file 44319_2024_309_MOESM1_ESM.pdf]

Table of Contents for Appendix Figure — Page number

Appendix Figure	Page number
**Appendix Figure S1.** Lick dynamics and timing	2
**Appendix Figure S2.** Spatial and sensory activity in enhanced, suppressed and none cells.	3
**Appendix Figure S3.** Activity dynamics in cell groups defined in the pre-sound period.	4
**Appendix Figure S4.** Activity surrounding the first lick in lick-matched trials.	6
**Appendix Figure S5.** Pre- and post-lick activity in Lick-matched trials.	9
**Appendix Figure S6.** Lick-matched trials - correlations.	10
**Appendix Figure S7.** Lick-matched trials - Euclidean distances.	11
**Appendix Figure S8.** Prediction accuracy in lick-matched trials.	12

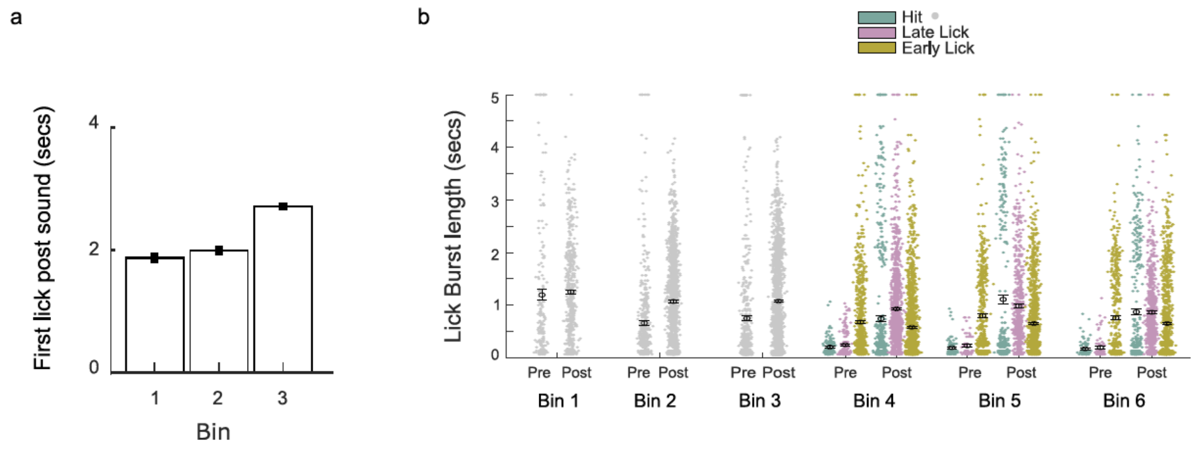

Appendix Figure S1. Lick dynamics and timing.

(a) Average timing of first lick post sound for training Bins 1, 2 & 3 (mean ± se). The timing of the first lick post-sound during training bins neared 3 secs, matching the water delivery timing (1-way Anova F=14.46 p=2.023e-07)

(b) Length of lick bursts. Same as Fig. 1h, but with down-sampled lick data, reducing the sampling rate from 500 Hz to 30 Hz to match the neural data sampling rate.

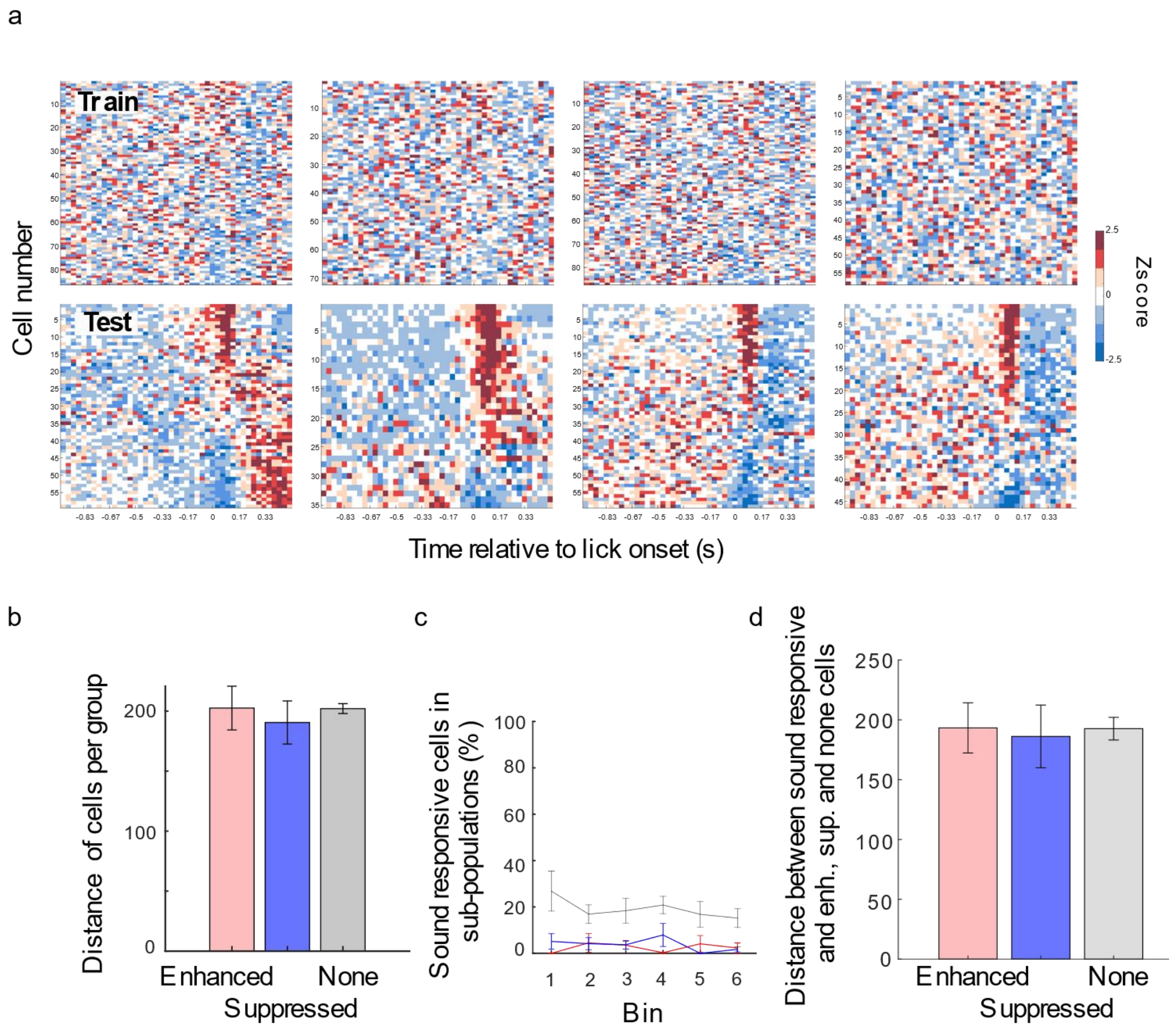

Appendix Figure S2. Spatial and sensory activity in enhanced, suppressed and none cells.

(a) Example of activity surrounding the first lick for train and test sessions as in Fig. 2b. Top: example of a training session - session 2 bin 1. Bottom: example of a testing session - session 15 bin 6.

(b) There was no significant difference in the Euclidian distance between cells in each cell group (mean ± se across sessions, 1-way ANOVA, F = 1.31, p = 0.27).

(c) The percentage of sound-responsive cells in the different sub-populations (mean ± se).

(d) Distance between sound-responsive cells and Enhanced, Suppressed, and None cells.

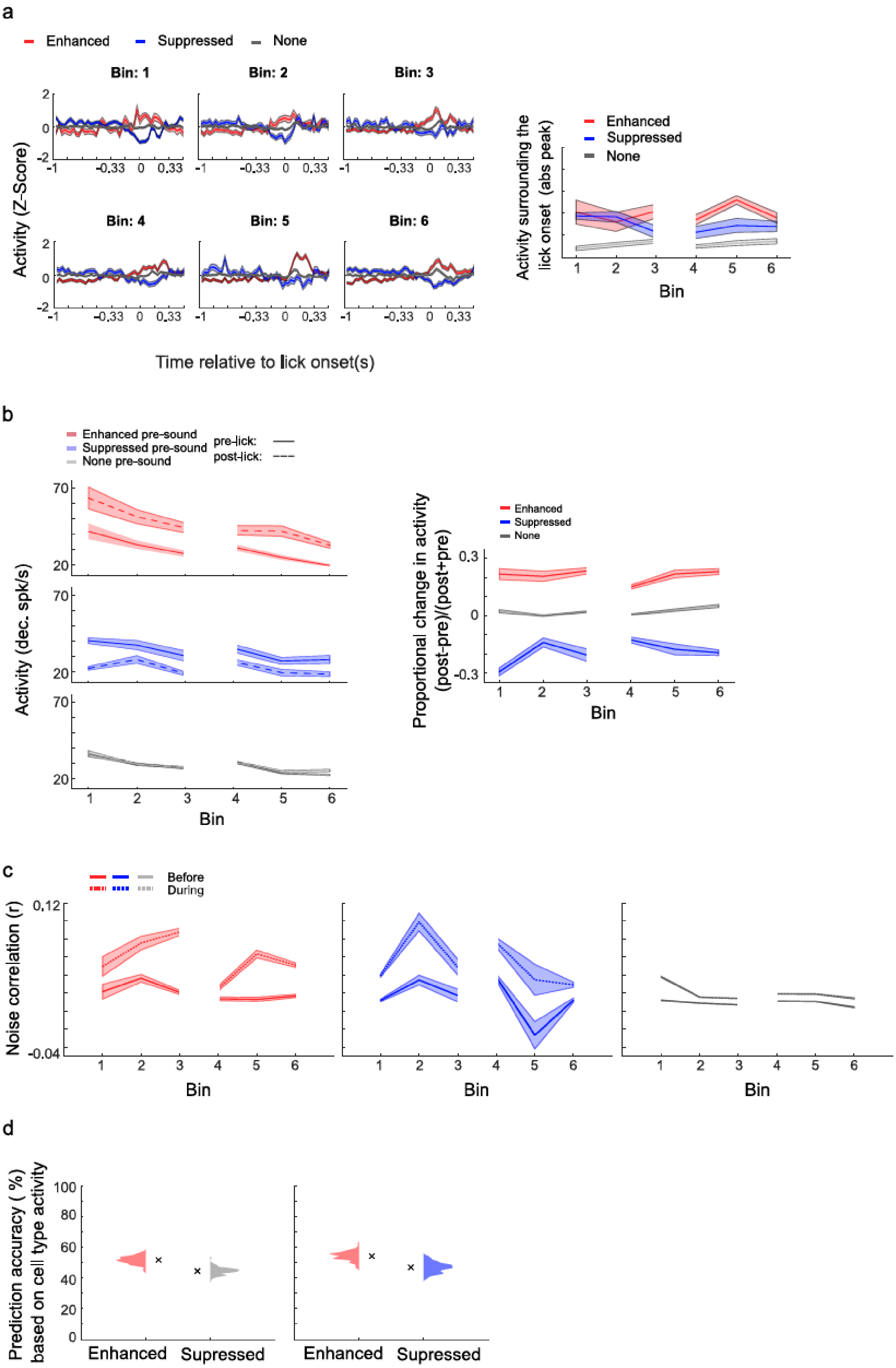
a
Enhanced
Suppressed
None
Bin: 1
Bin: 2
Bin: 3
Bin: 4
Bin: 5
Bin: 6
Activity (Z-Score)
Time relative to lick onset(s)
Activity surrounding the lick onset (abs peak)
Bin
b
Enhanced pre-sound
Suppressed pre-sound
None pre-sound
pre-lick:
post-lick:
Activity (dec. spk/s)
Proportional change in activity (post-pre)/(post+pre)
Bin
c
Before
During
Noise correlation (r)
Bin
d
Prediction accuracy (%) based on cell type activity
Enhanced
Supressed

Appendix Figure S3. Activity dynamics in cell groups defined in the pre-sound period.

(a) Left: Z-Scored activity surrounding the lick onset in the pre-sound period for: Enhanced-red, Suppressed-blue, and non-modulated-gray cells. Sessions were grouped by bin, shaded areas indicate se. The activity of cells was classified in the pre-sound period. Right: Absolute peak of z-scored activity in the period surrounding the first lick.

(b) Activity of enhanced, suppressed, and none cells before and after the lick. The activity of cells was classified in the pre-sound period. The activity was calculated as the average activity during the 165 ms before the lick (solid line) and the average activity during the 165 ms following the lick (dash line). Right: Proportional change in activity per cell period divided into enhanced, suppressed, and none groups. There was no significant increase in the ratio between pre- and post-lick activity as mice gained experience in the task (2-way ANOVA, cell types x bin interaction $F=2.2$, $p=0.06$ post-hoc, enhance cells Bin1 vs Bin6 $p=1$, Bonferroni corrected).

(c) Comparison of noise correlations of activity during and before the lick for the different cell types defined in the pre-sound period. “During”- a 330 ms window starting 165 ms before the lick, and “before” - a 330 ms window starting 495 ms before the lick.

(d) Prediction accuracy by cell type defined in the pre-sound period (Permutation tests, $p>0.05$ for all comparisons).

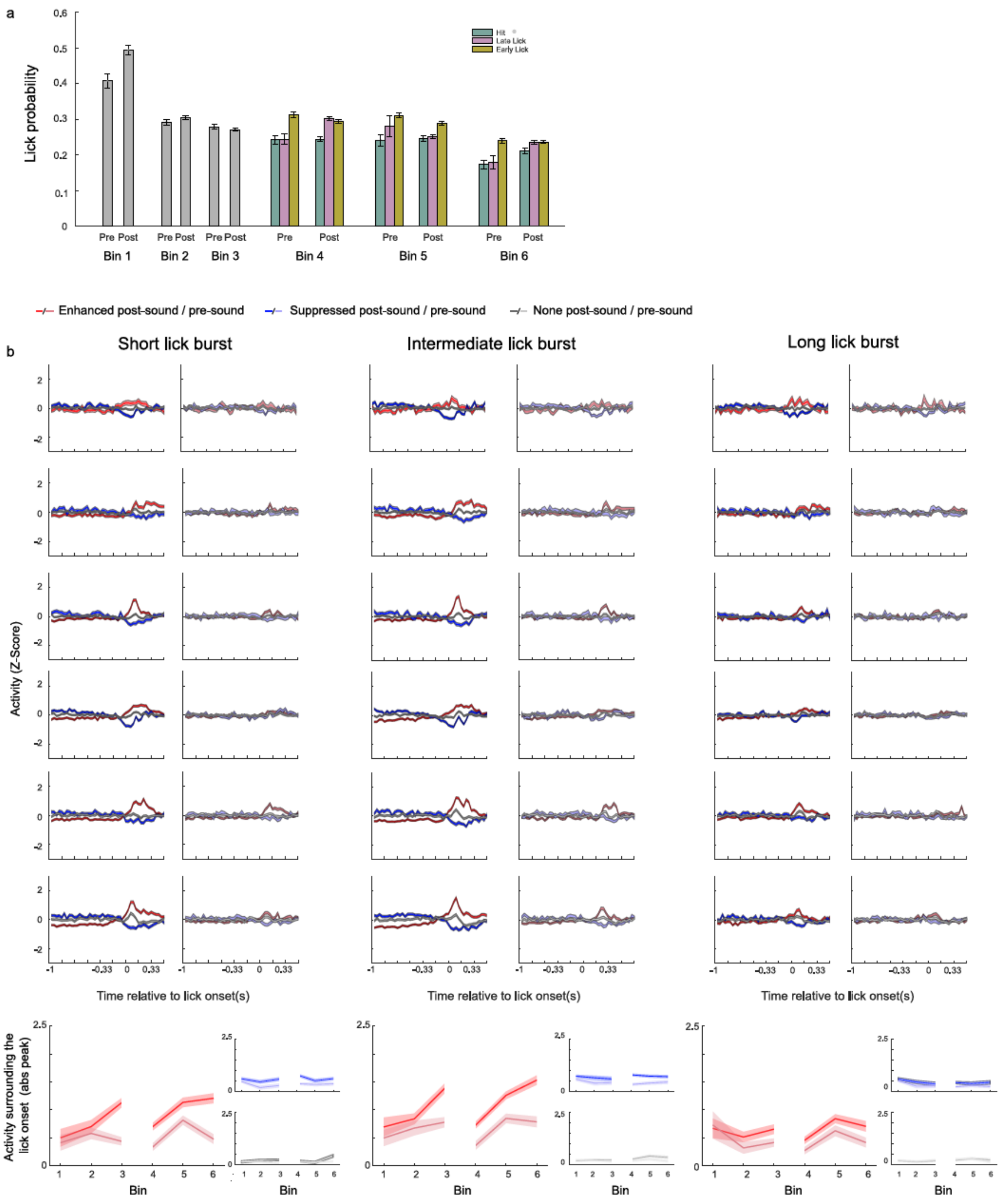
a
Lick probability
Hit
Late Lick
Early Lick
Pre Post
Bin 1
Bin 2
Bin 3
Bin 4
Bin 5
Bin 6
Enhanced post-sound / pre-sound
Suppressed post-sound / pre-sound
None post-sound / pre-sound
b
Short lick burst
Intermediate lick burst
Long lick burst
Activity (Z-Score)
Time relative to lick onset(s)
Activity surrounding the lick onset (abs peak)
Bin

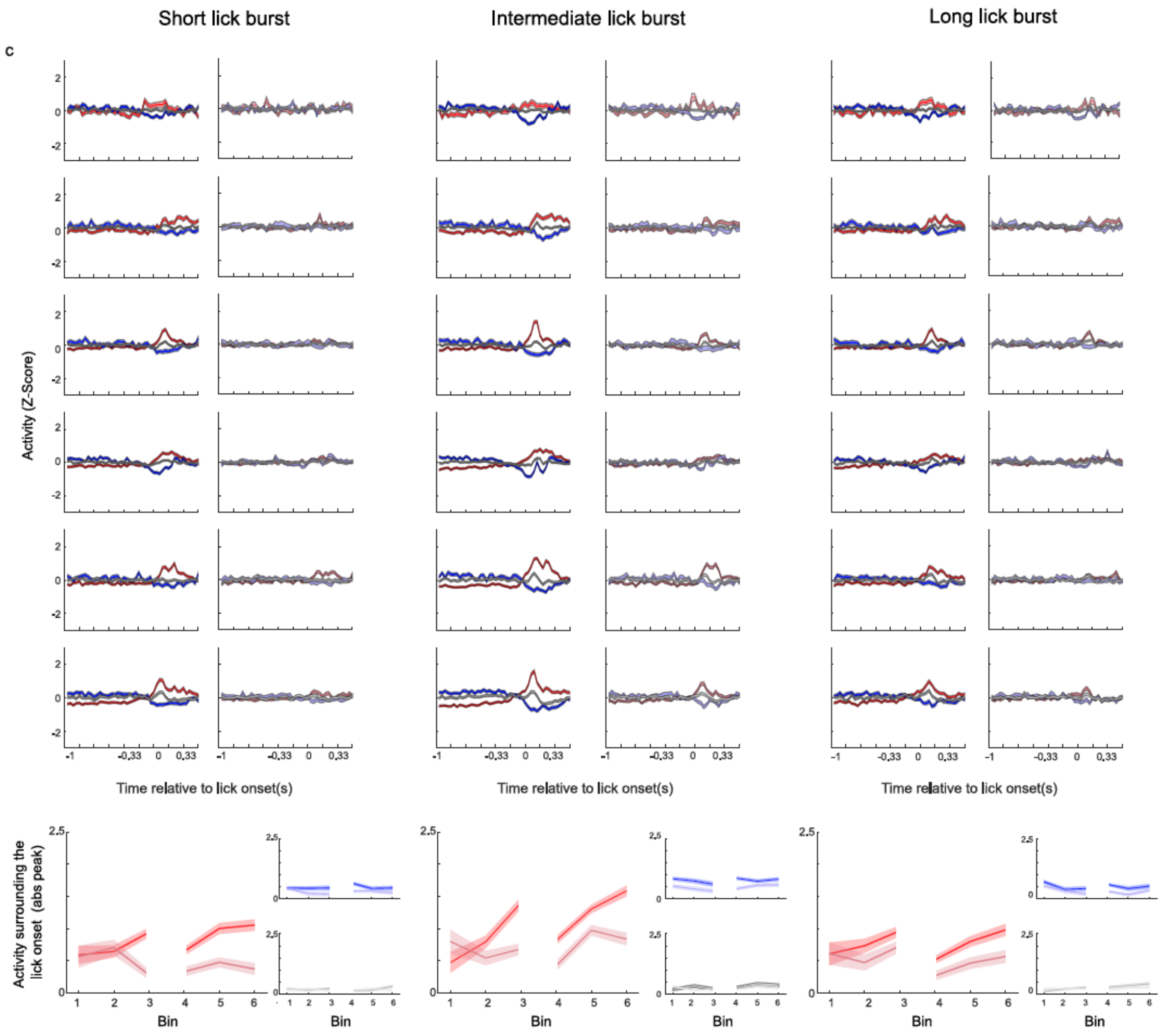

Appendix Figure S4. Activity surrounding the first lick in lick-matched trials.

(a) Lick probability in the 165 ms time window following the first lick in the pre- and post-sound periods for the different Bins and behavioral outcomes. We observed a general decrease in the lick probability following the first lick as mice gained experience with the task (2-way Anova, Bin: $F=142.9$ $p= 1.4e-148$).

(b) Z-Scored activity surrounding the first lick onset in the pre- and post-sound periods, for Enhanced-red, Suppressed-blue, and non-modulated-gray cells in lick-matched trials. The trials were divided into short, intermediate, and long lick bursts according to the length of the lick burst after the first lick post sound. Bottom: Absolute peak of z-scored activity in the period surrounding the lick. Same analysis as Fig. 2e for lick-matched trials.

(c) Same as (b) for down-sampled lick data. Here we also observed an increase in absolute peak activity around the lick in the post-sound period for both enhanced and suppressed cells and an increase in enhanced cell activity as the mice gained experience with the task (2-way ANOVA, short burst $F=31.16$ $p=2.6$ e-08 and $F=11.9$ $p=0.0006$ accordingly, intermediate $F=32.7$ $p=1.19$ e^{-08} and $F=30.9$ $p=3.2e^{-08}$ accordingly, long $F=15.1$ $p=0.0001$ and $F=15.5$ $p=0.0001$ accordingly).

(d) we observed, consistent with our findings above, & Appendix Figure S4c Down-sampled data:

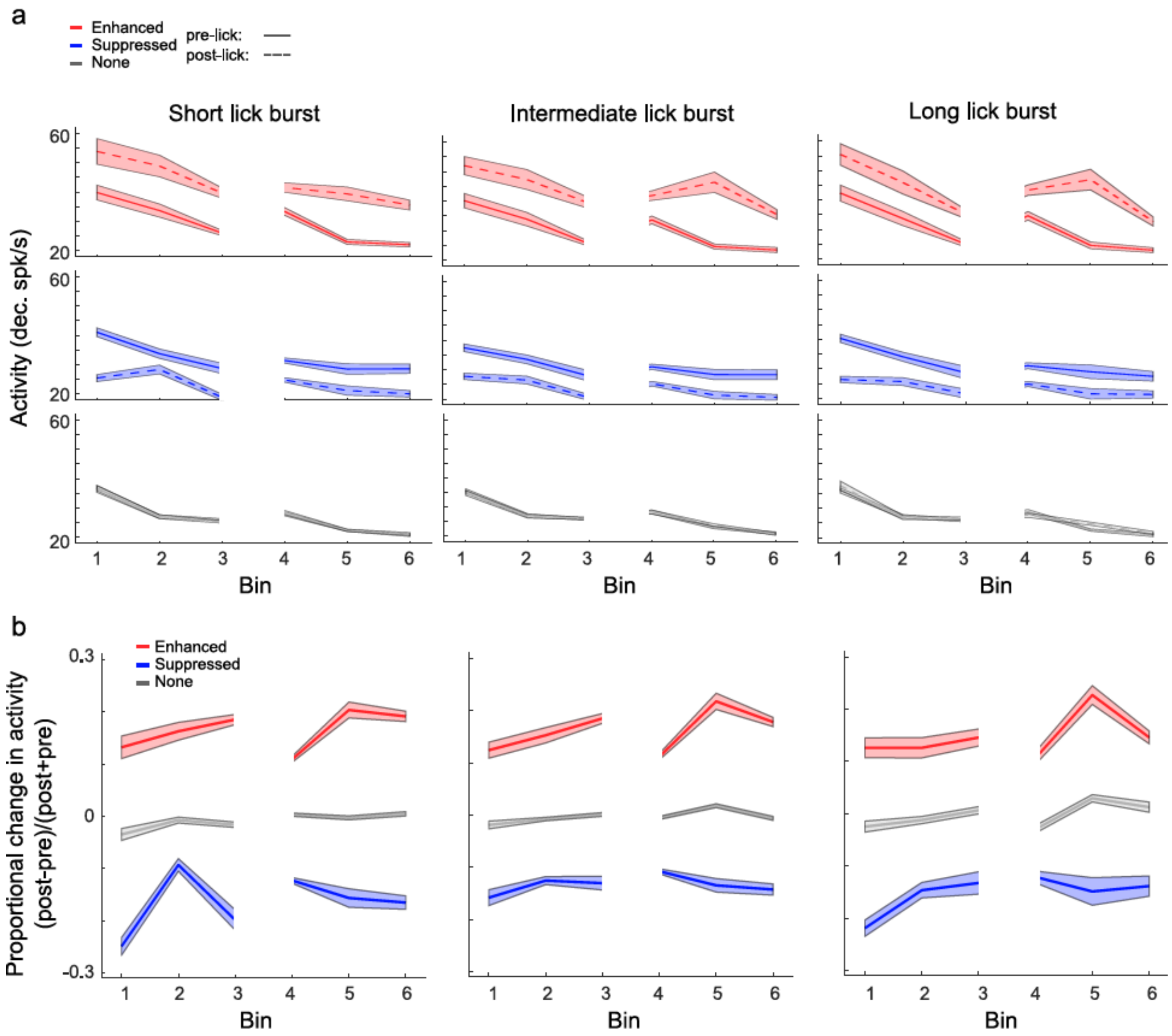

Appendix Figure S5. Pre- and post-lick activity in Lick-matched trials.

(a) Activity of enhanced, suppressed, and none cells before and after the lick in the pre-sound period. Same analysis as Fig. 2g for lick-matched trials.

(b) Proportional change in activity per cell in the pre-sound period. Same analysis as Fig. 2h for lick-matched trials. The more pronounced reduction in pre-lick activity (solid line) led to increased deltas of activity between the post and pre-lick periods for enhanced cells (2-way ANOVA, cell types x bin interaction short burst $F=11.21$, $p=3.7\ e^{-19}$, intermediate $F=8.25$ $p=2.2\ e^{-13}$, long $F=3.76$,$P=4.6\ e^{-05}$).

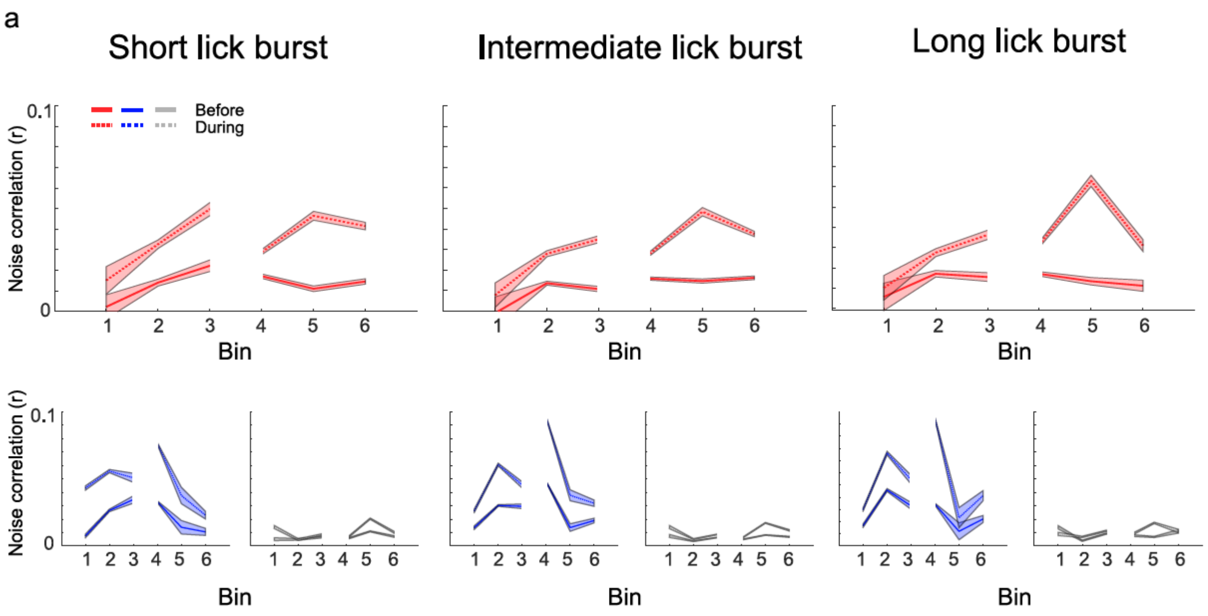

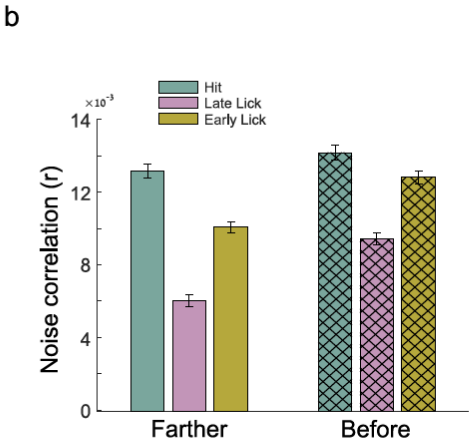

Appendix Figure S6. Lick-matched trials - correlations.

(a) Comparison of noise correlations in lick-matched trials during and before the first lick for the different cell types. "During"- a 330 ms window starting 165 ms before the lick, and "before" - a 330 ms window starting 495 ms before the lick. Same analysis as Fig. 3a for lick-matched trials. We consistently observed higher noise correlations for enhanced and suppressed cell groups during the lick window (2-way ANOVA, enhanced cells short burst $F=217.7$, $p=6.6\ e^{-49}$, intermediate $F=321$ $p=3.6\ e^{-71}$, long $F=206$,$P=5.2\ e^{-58}$, suppressed cells short burst $F=178$, $p=1.6\ e^{-40}$, intermediate $F=197$ $p=1.1\ e^{-44}$, long $F=144$,$P=3.8\ e^{-33}$).

(b) Comparison of noise correlations in Hit, LL, and EL trials during two windows before the lick onset: "before" – the same window as above, and "farther" – a 330 ms window starting 825 ms before the lick. Same analysis as Fig. 3b for matched intermediate lick-burst trials.

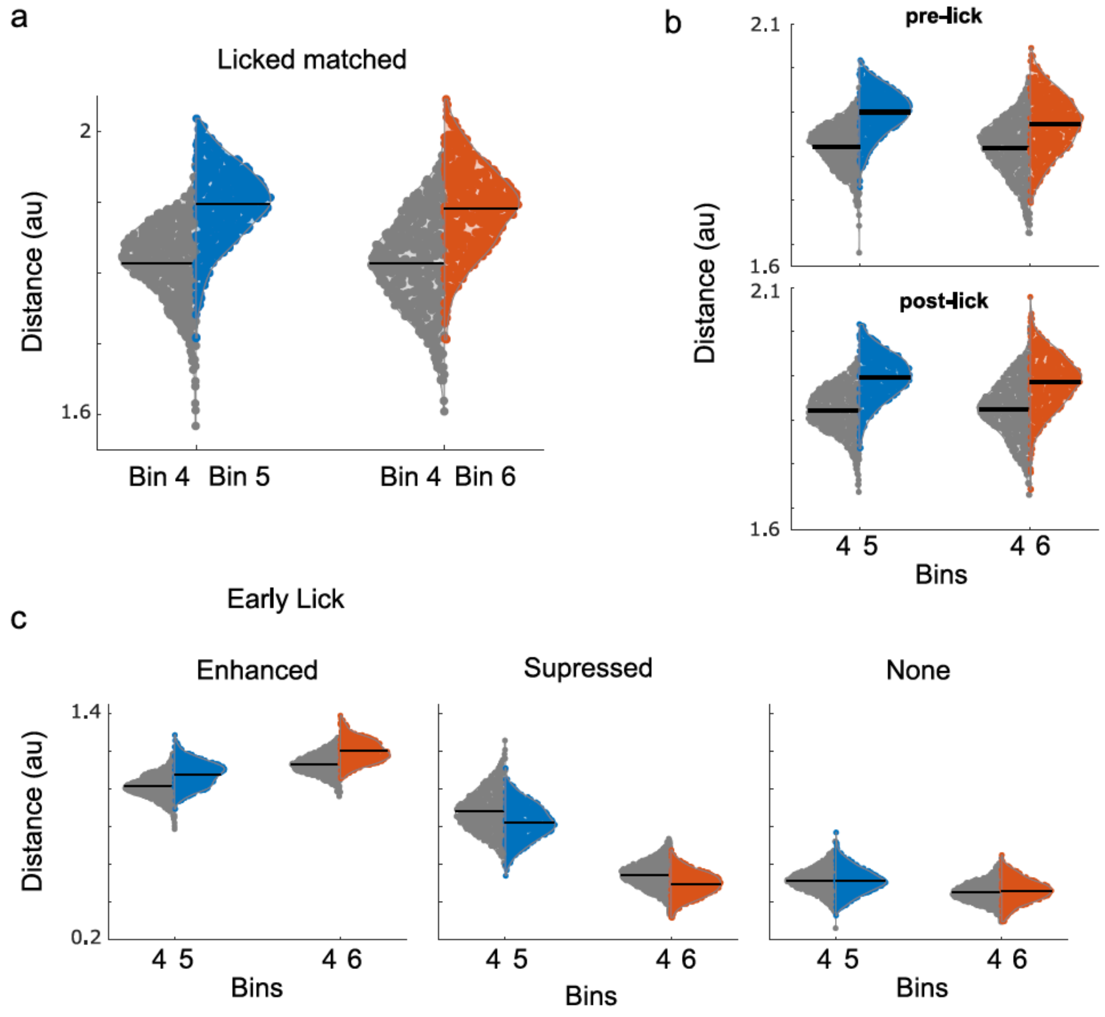

Appendix Figure S7. Lick-matched trials - Euclidean distances.

(a) Euclidean distances between lick-matched (intermediate burst length) Hit and LL trials. Same analysis as in Fig. 4b for lick-matched trials.

(b) Euclidean distances between Hit and LL trials in the pre- and post-lick periods.

(c) Euclidean distances between Hit and EL trials for Enhanced, Suppressed and None cells.

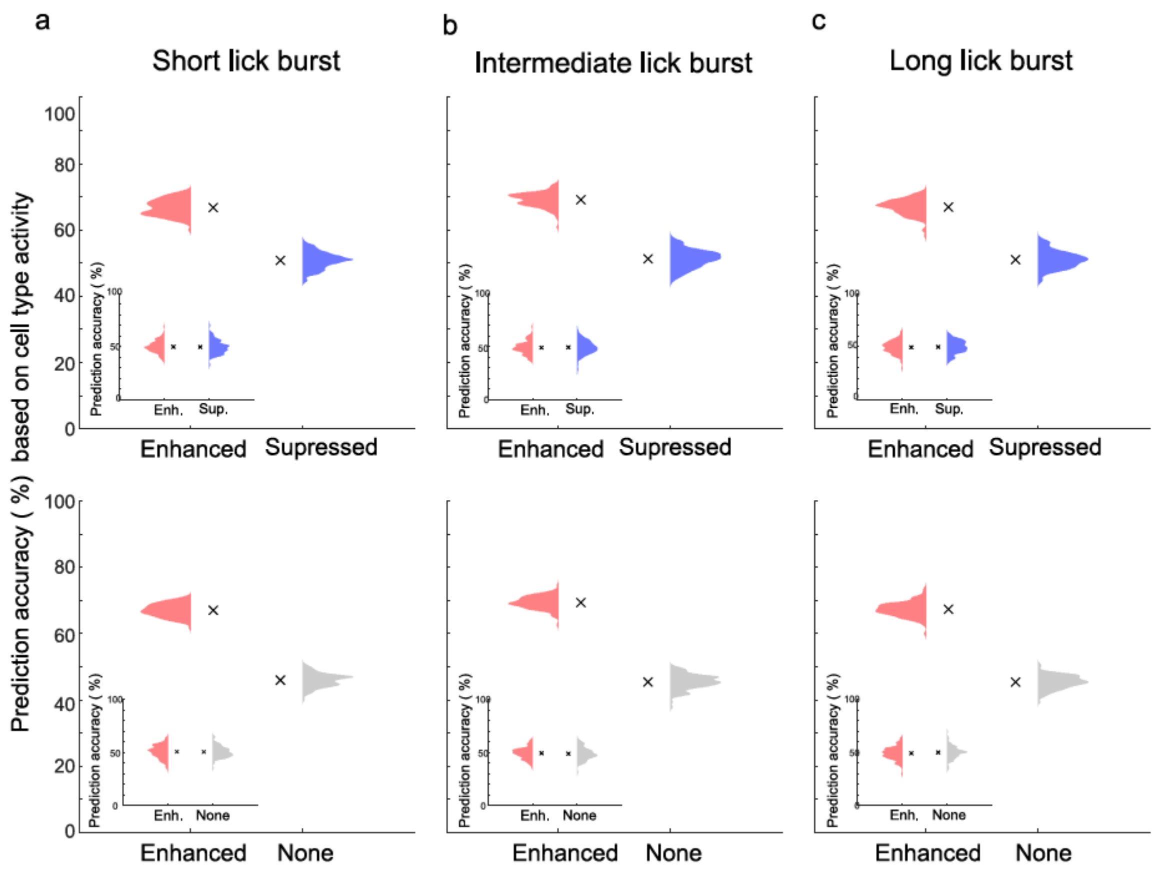

Appendix Figure S8. Prediction accuracy in lick-matched trials.

Prediction accuracy by cell type activity in lick-matched trials: (a) Short lick bursts, (b) intermediate lick bursts, and (c) long lick bursts. X marker represents the mean prediction accuracy for licked-matched trials. Inset – same analysis with shuffled labels. Top: prediction of Enhanced cells vs Suppressed cells. Bottom: prediction of Enhanced cells vs None cells. Same analysis as in Fig. 5b for lick-matched trials.
