# Supplementary material for: Experience-driven development of decision-related representations in the auditory cortex
Source: EMBO Rep. 2024 Nov 11;26(1):84–100. doi: 10.1038/s44319-024-00309-0 (PMC11723978; doi:10.1038/s44319-024-00309-0)
Supplement: Supplementary file 2 — Peer Review File [file 44319_2024_309_MOESM2_ESM.pdf]

EMBO reports

Experience-driven development of decision-related representations in the auditory cortex

Jennifer Resnik, Itay Kazanovich, and Shir Itzhak

Corresponding author(s): Jennifer Resnik (resnikj@bgu.ac.il)

Review Timeline:

Submission Date:	27th Mar 24
Editorial Decision:	14th May 24
Revision Received:	12th Sep 24
Editorial Decision:	10th Oct 24
Revision Received:	15th Oct 24
Accepted:	22nd Oct 24

Editor: Esther Schnapp

Transaction Report:

(Note: With the exception of the correction of typographical or spelling errors that could be a source of ambiguity, letters and reports are not edited. Depending on transfer agreements, referee reports obtained elsewhere may or may not be included in this compilation. Referee reports are anonymous unless the Referee chooses to sign their reports.)

1st Editorial Decision 14th May 2024

Dear Dr. Resnik

Thank you for the submission of your manuscript to EMBO reports. We have now received the full set of referee reports that is pasted below.

As you will see, the referees acknowledge that the findings are interesting. However, they also have some suggestions for how the study could be improved. I think the main concern raised by referee 1 is important, and the other referees seem to raise a similar point. I would like to invite you to address all concerns. Please let me know if you have any comments or questions regarding the revisions and we can discuss this further, also in a video chat, if you wish.

I would thus like to invite you to revise your manuscript with the understanding that the referee concerns must be fully addressed and their suggestions taken on board. Please address all referee concerns in a complete point-by-point response. Acceptance of the manuscript will depend on a positive outcome of a second round of review. It is EMBO reports policy to allow a single round of major revision only and acceptance or rejection of the manuscript will therefore depend on the completeness of your responses included in the next, final version of the manuscript.

We realize that it is difficult to revise to a specific deadline. In the interest of protecting the conceptual advance provided by the work, we recommend a revision within 3 months (14th Aug 2024). Please discuss the revision progress ahead of this time with the editor if you require more time to complete the revisions.

You can either publish the study as a short report or as a full article. For short reports, the revised manuscript should not exceed 27,000 characters (including spaces but excluding materials & methods and references) and 5 main plus 5 expanded view figures. The results and discussion sections must further be combined, which will help to shorten the manuscript text by eliminating some redundancy that is inevitable when discussing the same experiments twice. For a normal article there are no length limitations, but it should have more than 5 main figures and the results and discussion sections must be separate. In both cases, the entire materials and methods must be included in the main manuscript file.

IMPORTANT NOTE: we perform an initial quality control of all revised manuscripts before re-review. Your manuscript will FAIL this control and the handling will be DELAYED if the following APPLIES:
1) A data availability section providing access to data deposited in public databases is missing. If you have not deposited any data, please add a sentence to the data availability section that explains that.
2) Your manuscript contains statistics and error bars based on n=2. Please use scatter blots in these cases. No statistics should be calculated if n=2.

When submitting your revised manuscript, please carefully review the instructions that follow below. Failure to include requested items will delay the evaluation of your revision.

1) a .docx formatted version of the manuscript text (including legends for main figures, EV figures and tables). Please make sure that the changes are highlighted to be clearly visible.

2) individual production quality figure files as .eps, .tif, .jpg (one file per figure). See https://wol-prod-cdn.literatumonline.com/pb-assets/embo-site/EMBOPress_Figure_Guidelines_061115-1561436025777.pdf for more info on how to prepare your figures.

3) We replaced Supplementary Information with Expanded View (EV) Figures and Tables that are collapsible/expandable online. A maximum of 5 EV Figures can be typeset. EV Figures should be cited as 'Figure EV1, Figure EV2" etc... in the text and their respective legends should be included in the main text after the legends of regular figures.

- For the figures that you do NOT wish to display as Expanded View figures, they should be bundled together with their legends in a single PDF file called *Appendix*, which should start with a short Table of Content. Appendix figures should be referred to in the main text as: "Appendix Figure S1, Appendix Figure S2" etc. See detailed instructions regarding expanded view here: <https://www.embopress.org/page/journal/14693178/authorguide#expandedview>

- Additional Tables/Datasets should be labeled and referred to as Table EV1, Dataset EV1, etc. Legends have to be provided in a separate tab in case of .xls files. Alternatively, the legend can be supplied as a separate text file (README) and zipped together with the Table/Dataset file.

4) a .docx formatted letter INCLUDING the reviewers' reports and your detailed point-by-point responses to their comments. As part of the EMBO Press transparent editorial process, the point-by-point response is part of the Review Process File (RPF), which will be published alongside your paper.

5) a complete author checklist, which you can download from our author guidelines <https://www.embopress.org/page/journal/14693178/authorguide>. Please insert information in the checklist that is also

reflected in the manuscript. The completed author checklist will also be part of the RPF.

6) Please note that all corresponding authors are required to supply an ORCID ID for their name upon submission of a revised manuscript (<https://orcid.org/>). Please find instructions on how to link your ORCID ID to your account in our manuscript tracking system in our Author guidelines
<https://www.embopress.org/page/journal/14693178/authorguide#authorshipguidelines>

7) Before submitting your revision, primary datasets produced in this study need to be deposited in an appropriate public database (see https://www.embopress.org/page/journal/14693178/authorguide#datadeposition). Please remember to provide a reviewer password if the datasets are not yet public. The accession numbers and database should be listed in a formal "Data Availability" section placed after Materials & Method (see also https://www.embopress.org/page/journal/14693178/authorguide#datadeposition). Please note that the Data Availability Section is restricted to new primary data that are part of this study. * Note - All links should resolve to a page where the data can be accessed. *
If your study has not produced novel datasets, please mention this fact in the Data Availability Section.

8) At EMBO Press we ask authors to provide source data for the main manuscript figures. Our source data coordinator will contact you to discuss which figure panels we would need source data for and will also provide you with helpful tips on how to upload and organize the files.

9) Our journal also encourages inclusion of *data citations in the reference list* to directly cite datasets that were re-used and obtained from public databases. Data citations in the article text are distinct from normal bibliographical citations and should directly link to the database records from which the data can be accessed. In the main text, data citations are formatted as follows: "Data ref: Smith et al, 2001" or "Data ref: NCBI Sequence Read Archive PRJNA342805, 2017". In the Reference list, data citations must be labeled with "[DATASET]". A data reference must provide the database name, accession number/identifiers and a resolvable link to the landing page from which the data can be accessed at the end of the reference. Further instructions are available at https://www.embopress.org/page/journal/14693178/authorguide#referencesformat

10) Regarding data quantification (see Figure Legends: https://www.embopress.org/page/journal/14693178/authorguide#figureformat)

The following points must be specified in each figure legend:

- the name of the statistical test used to generate error bars and P values,

- the number (n) of independent experiments (please specify technical or biological replicates) underlying each data point,

- the nature of the bars and error bars (s.d., s.e.m.),

- If the data are obtained from n<2, use scatter blots showing the individual data points.

Discussion of statistical methodology can be reported in the materials and methods section, but figure legends should contain a basic description of n, P and the test applied.

- Please also include scale bars in all microscopy images.

11) The journal requires a statement specifying whether or not authors have competing interests (defined as all potential or actual interests that could be perceived to influence the presentation or interpretation of an article). In case of competing interests, this must be specified in your disclosure statement. Further information: https://www.embopress.org/competing-interests

We would also welcome the submission of cover suggestions, or motifs to be used by our Graphics Illustrator in designing a cover.

As part of the EMBO publication's Transparent Editorial Process, EMBO reports publishes online a Review Process File (RPF) to accompany accepted manuscripts. This File will be published in conjunction with your paper and will include the referee reports, your point-by-point response and all pertinent correspondence relating to the manuscript.

You are able to opt out of this by letting the editorial office know (emboreports@embo.org). If you do opt out, the Review Process File link will point to the following statement: "No Review Process File is available with this article, as the authors have chosen not to make the review process public in this case."

I look forward to seeing a revised form of your manuscript when it is ready.

Best regards,
Esther

Esther Schnapp, PhD
Senior Editor
EMBO reports

Referee #1:

Summary
1. Does this manuscript report a single key finding? YES
The main finding of the paper is that motor-related responses in the auditory cortex are impacted by task learning and performance.

2. Is the reported work of significance (YES), or does it describe a confirmatory finding or one that has already been documented using other methods or in other organisms etc (NO)? YES
This is the first report to my knowledege that specifically tracks motor responses in auditory cortex during the task.

3. Is it of general interest to the molecular biology community? NO
This paper is of general interest to the integrative neuroscience community but does not appear relevant for molecular biology.

4. Is the single major finding robustly documented using independent lines of experimental evidence (YES), or is it really just a preliminary report requiring significant further data to become convincing, and thus more suited to a longer¬format article (NO)? UNDECIDED
The report documents its findings well but as I point out below there are major oversights in the analysis that weaken the main conclusions. I think these can be addressed within the format of the article but require substantive revision

Main report
In this study, the authors record neural activity in the auditory cortex as mice progress through various stages of learning an auditory task. This interesting data set allows them to document how responses, in particular to motor activity (lick responsiveness), change throughout the task. They find a striking change in lick responsiveness that increases as animals learn to link sounds with the licking and between periods in which licks are likely sound-driven vs random. This raises the interesting possibility that motor signals in the auditory cortex may be gated by their auditory task relevance.
These findings rely on the task design in the report that separates in time the sound-driven activity and the motor/decision related activity by teaching mice to wait to lick. However, the task design and in particular the analysis provided do not allow to disambiguate changes in motor activity (ie more or less licking) from the task-related changes that the authors claim are linked to neural activity changes. Without a clearer comparison and accounting for potential differences, the author's claim that the changes in neural activity are "decision-related" and not the consequence of task-driven changes in motor behavior cannot be well supported. This is the main limitation of the study which I comment more amply below and encourage the authors to address thoroughly.
Lick dynamics : Since the main point of the paper is to disentangle purely motor and task-modulated motor activity, a good control of the motor activity itself is essential. The licks are only very cursorily described (fig1b), it is not clear whether mice lick multiple times in a row, whether this differs between pre/post sound and trial types and how the authors deal with this in their analysis of neural responses to licks. A clear quantification of number of licks, lick timing etc for each trial type and stage should be added to the manuscript
Changes in licking amplitude or dynamics constitutes a potentially major confound for multiple findings, if there are indeed changes in licks controlling for this bias is crucial by calculating partial correlation or selecting subsets of trials with matched licks :
- The evolution in neural responses to licks throughout training if licking profiles evolve in time
- The different level of synchrony of activity (fig 3) between trial types, if there are multiple, potentially regular, licks this could trigger synchronized firing
- The difference between neural trajectories in fig 4
- The decoding in figure 5 : can pre- and post-sound licks simply be discriminated based on the fact that the licks are more vigorous/synchronous/numerous?
Training phase : The authors highlight that during the training phase, mice don't need to attend to the sound to get water but nonetheless the mice do seem to use the sound to sculpt their behavior during this phase. The authors suggest that during this period what is going on is mainly familiarization with the environment etc but clearly figure 1 shows that mice are using the sound to modify their behavior since they increase the probability of post-sound licks and decrease the miss trials. It would be interesting to know whether the animals are also already waiting to lick (since water only comes after 3s) during this phase. This would clarify the nature of the change in behavior between the training and testing phase.
Relation to literature : Literature on "innnate", non task related motor activity in the cortex and its association with sensory

responses is not well-cited and should be used to contextualize the reports findings (ex: Bimbard et al, 2023, Lohuis et al, 2024). This is important since the authors report essentially no lick responses outside the context of the task. In particular the studies by Schneider et al are cited but the fact that they show activity changes related to motor activity per se, at odds with the current study, should be clearly discussed and reasons for discrepancies explained.
The authors discuss the importance of separating in time the stimulus, the decision and the motor action to disambiguate between them and they indeed provide a task that allows for this. However since they mainly analyse lick responses it's not clear what is being separated. Could the authors clarify their strategy given the actual behavior in the task and how this relates to other studies?
Neural response interpretation : The authors should describe any global changes in neural activity level that could accompany task engagement and learning (this has been reported in many studies : Otazu et al, 20019; Rodgers et al, 2014; Bagur et al, 2018). Such changes can lead to apparent changes in lick responsiveness driven by lower baselines for example. Spontaneous activity can be hard to evaluate in calcium imaging but sound responses could be used for comparison for example.
Sensory properties : The authors comment that the lick reactive cells are non-sensory, could they provide a figure describing this since? I understand this is not the main point of the paper but the relation between lick responses and sound responses will be of interest to many people?

Minor points :
- Could the authors add a time bar to fig 1a?
- Could they clarify between what types of trials they calculate the dprime in figure1
- Could the authors clarify the factors that were added to the decoder in the lower part of fig 5b?
- Lines 413-416 : the authors link their findings that mainly enhanced cells encode pre vs post licks and link this to the paper by Francis et al. I am not sure the link they make is clear here since the "small neuronal networks" referred to by this paper correspond to sparsely connected small sets of cells. Could they clarify what they meant?
- I am pretty sure the authors are not tracking the same neurons across days, could they just clarify this in the methods ?
- Figures 2e and 2i show contradictory results regarding the overall trend for enhanced and suppressed cells (change vs no change). It seems that this may be due to the fact that one analysis is done on z-scored activity and not the other (as far as I could gather from the legends that are not clear on this point). Given that the peaks become narrower in time in d, if the traces are independently z-scored this will also enhance their height. Could the authors be clearer on what is quantified here?

Referee #2:

Description: This is an interesting paper that adds new evidence about experience-driven plasticity in the auditory cortex in a mouse model. The paper has a good experimental design that enables the differentiation of decision-making-related, reward-related, and motor-related neural activity from those that could be modified by experience. The authors found that as mice gain more experience with the auditory task, there were more enhanced neurons, whose activity was enhanced by lick behavior. Further, the performance of decoders designed to differentiate the neural encoding of lick-behavior occurred with/without behavioral outputs also depends on the experience of mice. To sum up, the paper provided evidence that experience-driven modifications on decision-making representations of A1 neurons in a mice model. Our comments below are meant to improve this manuscript:

Major concerns:
1. The strongest evidence demonstrating that plasticity in A1 is sensory-experience-driven instead of motor-driven is comparing post-sound and pre-sound licks in experienced mouse. Have you considered how the large trial imbalance between pre-sound and post-sound licks affects the other analyses except the decoding? It will be helpful to includes the percentage of pre-sound licks for bin 4-6 in Figure 1g.

2. From the Method section of dimensionality reduction analysis and Figure 4, it seems that the authors conducted their PCA analysis for data recorded in each bin separately and then calculated the Euclidian distances within each PCA space. We do not believe it is appropriate to compare Euclidean distances that were calculated in different PCA spaces. Even when you keep incorporate all of the PCs, it does not guarantee the Euclidean distance calculated in different spaces are at the same scale. Consequently, you might find different Euclidean distances when projecting the same pair of datapoints into different PCA spaces, although there is only one ground truth distance. We suggest the authors use distribution alignment, hierarchical Wasserstein alignment, or CCA to align different spaces before comparing Euclidean distances calculated in different spaces (see Dabagia, M., Kording, K.P. & Dyer, E.L. Aligning latent representations of neural activity. Nat. Biomed. Eng 7, 337-343 (2023). https://doi.org/10.1038/s41551-022-00962-7).

3. Fig. 2e&i,j & line197: How do the authors interpret their finding that neurons show decreased average spiking activity in the pre- and post-lick window but an increased absolute peak firing rate during the same time window? If it is because more cells that are spatially overlapped and firing more weakly individually, then wouldn't we also expect a decrease of the pair-wise distance within each cell group in the test bins relative to those in the training bins? Also, to delineate the "purely" sensory -related and experience-driven plasticity, wouldn't an examination of the post-sound and pre-sound activity be a cleaner

analysis? This removes any motor-related and experience-driven plasticity. Indeed, from our eye, it looks like there is also experience induced effect for pre-sound licks in fig. 2e and extended fig. 2.

4. Line 101-106: It is not clear the behavioral difference between a late lick and a miss trial. From a signal detection point of view, they would be the same.

Minor concerns:
The grey shading around lines in the figures makes it difficult to distinguish line colors. The trajectories plots need more details about meanings of dashed vs solid line, squares, stars, and circles. It would be more helpful if the authors showed 95% confidence intervals as error bars instead of SEMs.

Line 128: Fig. 2g should be fig. 1g.

Fig. 1a: Please clarify the duration of the lower water reward window.

Fig. 2h: Please provide a legend and better color choices for all lines. We find it hard to parse the different line colors.

Fig. 4: Shouldn't the figure legend a comparison between late lick vs hit trials instead of miss vs hits trials?

Line 152: There is not a fig. 1h.

Line 162: Are all trials with licks before or after sounds used to delineate the characteristics of cells? Will the experience effects of enhanced cells be the same if you just use trials with pre-sound licks when delineating the cells' characteristics?

Line 190-191: Please discuss in more detail how you spatially segregated the different cell groups.

Line 192: It may be helpful to provide a figure showing the spatial overlap between lick-responsive and sound-responsive cells.

Line 220: Can the authors discuss the difference in the findings of bin4 in fig. 2i and fig. 2e.

Line 223: how the decrease of spike difference between pre- and post- lick period explains the decreased peak activity during these two lick windows?

Line 245: We believe there is a missing word: 'increase'?

Line303: Please remove the extra 'the'.

Referee #3:

Summary
The authors aim to build on recent studies demonstrating that sensory cortices represent aspects of decision-making in addition to stimuli-related information by asking whether decision-related activity in auditory cortex is innate or acquired throughout learning. Chronic 2-photon imaging allows them to record changes in fluorescent calcium signaling longitudinally while mice learn an auditory detection task with a delayed response window. As they point out, this delayed window is important in helping segregate cue-related activity from decision-related activity and is a step often skipped by other studies. They find that subsets of auditory cortical cells either increase or decrease as animals report the stimuli and that these responses develop and become more synchronized with training. The activity of these subsets can decode whether a lick occurred before or after the stimulus. The paper was very well written, with clear, concise explanations of the experiments and the logic behind them, making it an easy paper to read and understand. My comments on the paper are largely minor comments and the majority pertain to the figures, which I believe could better complement the text with minor alterations.

The main text doesn't describe this, but the task design included catch trials. Performance on these trials was used to calculate d' for Fig 1F. People familiar with psychophysics will understand what d' represents and that the numbers in Fig 1F are quite good, but others could benefit from a brief explanation in the main text. The methods for calculating this should also be included in the methods.
Animals are first trained on a Pavlovian task (Bins 1-3) and then trained on an operant task (Bins 4-6). Oftentimes, comparisons were made between Bin 1 and Bin 6, but I wonder whether this is a fair comparison to make given how the task changes between those two bins and how often they see the activity "reset" when the task changes. For example, in Figure 2E there doesn't seem to be a difference between Bin 1 and Bin 6, but there does seem to be a difference between Bin 1 & 3 (first and last bins of Pavlovian) and between Bins 4 & 6 (first and last bins of operant).

Figure-related minor points

Generally speaking, the figures could benefit from markers of statistical significance, since a lot of the differences are so subtle but are significant.
In Figure 1 and Figure 4, black bars are used with dark data points and black confidence intervals, which makes it hard to read. Open bars might work better to show the other features of the graphs.
Figure 1B is not mentioned in the text and it is unclear what kind of session this data is from, Pavlovian or test? Are there differences in the distributions in the two task versions?
Figure 2 & Figure 4: Font sizes should be standardized and, in many places, much larger. It was difficult to read much of the text in these figures.
Figure 2: These colors are also very hard to distinguish. The pastels largely look the same, and in many places, the error bars are gray no matter what color the mean line is. The legends are so small that it's hard to tell what is what.
Figure 2E / I / F: The authors repeatedly make the point that the responses seem to reset at the start of Bin 4 when the task rules change. However, here the data is plotted continuously between Bins 3 and 4, which masks that rest a bit. Having a break in the line plot between Bins 3 and 4 would make that reset more obvious while also emphasizing the timepoint where the rules change.
Figure 2I: The usage of the word "activity" seems inconsistent across panels. It's unclear which panels represent the activity overall vs. the activity of specific cells, and I think this is an important distinction given how the overall population activity increases while specific cells seem to decrease their responses (if I'm interpreting this correctly).
Figures 2 & 3: The colors/line styles are very hard to distinguish, especially in panels 2C, 2F, and 3A. Gray is used to denote cells that do not respond to the lick, but the confidence intervals are often gray no matter the response type, which makes the lines blur together.
Figure 4A / 4D: The text says Hit vs LL while the figure says Hit vs Miss.
Figure 4B: I'm not sure whether having all of the individual data points plotted is helpful here. It might be clearer to just have the errorbars.
Figure 5A could benefit from a legend, while Figure 5B could benefit from some labels to help denote the difference between the graphs on the top vs the graphs on the bottom

Non-Figure minor points:
Line 42: ACtx is defined later in the main text, but it should be defined here
Line 128: incorrect figure reference - maybe 1G? 1B?
Lines 147-148: What was the logic for picking these two sessions?
Line 152: Incorrect figure reference (in general would double check the figure references for Fig 2, sometimes the context of the sentence seemed to better match a different panel in the figure than the one cited)
Line 245: "showing an in the strength" - showing an increase in the strength?
Line 291: "Distance difference" is only defined in the next sentence, so there might be a clearer way to introduce this here - "difference in the distance between pre- and post-lick periods."
Line 364: "time interval", this period is referred to as a delay everywhere else, and could be changed for consistency

We appreciate the reviewers' thorough evaluation of our work, as well as their positive comments and constructive feedback, which have allowed us to improve our manuscript significantly.

Based on the reviewers' recommendation, we have thoughtfully analyzed the lick activity during the task's different stages and behavioral outcomes and repeated all our main analyses using lick-matched trials. The results of these analyses are presented in **New Fig. 2b**, **New Fig. 2h**, **New Appendix Figure S1b**, **New Appendix Figure S4a-c**, **New Appendix Figure S5**, **New Appendix Figure S6**, **New Appendix Figure S7**, and **New Appendix Figure S8**.

Additionally, we revisited our analyses of discriminability between behavioral choices using canonical correlation analysis to align the different spaces (**New Fig. 5**), included details about sound-responsive cells (**New Fig. 3** and **New Appendix Figure S2d**), and further examined activity in the pre-sound period (**New Appendix Figure S3**). Based on the reviewers' suggestions, we have also refined the introduction, results, discussion, and methods sections of the manuscript.

We believe that these revisions effectively address the reviewers' comments and have significantly strengthened the manuscript. Please find our detailed responses to each reviewer's comment below.

Referee #1:
Main report
In this study, the authors record neural activity in the auditory cortex as mice progress through various stages of learning an auditory task. This interesting data set allows them to document how responses, in particular to motor activity (lick responsiveness), change throughout the task. They find a striking change in lick responsiveness that increases as animals learn to link sounds with the licking and between periods in which licks are likely sound-driven vs random. This raises the interesting possibility that motor signals in the auditory cortex may be gated by their auditory task relevance.
These findings rely on the task design in the report that separates in time the sound-driven activity and the motor/decision related activity by teaching mice to wait to lick. However, the task design and in particular the analysis provided do not allow to disambiguate changes in motor activity (ie more or less licking) from the task-related changes that the authors claim are linked to neural activity changes. Without a clearer comparison and accounting for potential differences, the author's claim that the changes in neural activity are "decision-related" and not the consequence of task-driven changes in motor behavior cannot be well supported. This is the main limitation of the study which I comment more amply below and encourage the authors to address thoroughly.
Lick dynamics : Since the main point of the paper is to disentangle purely motor and task-modulated motor activity, a good control of the motor activity itself is essential. The

licks are only very cursorily described (fig1b), it is not clear whether mice lick multiple times in a row, whether this differs between pre/post sound and trial types and how the authors deal with this in their analysis of neural responses to licks. A clear quantification of number of licks, lick timing etc for each trial type and stage should be added to the manuscript
Changes in licking amplitude or dynamics constitutes a potentially major confound for multiple findings, if there are indeed changes in licks controlling for this bias is crucial by calculating partial correlation or selecting subsets of trials with matched licks :
- The evolution in neural responses to licks throughout training if licking profiles evolve in time
- The different level of synchrony of activity (fig 3) between trial types, if there are multiple, potentially regular, licks this could trigger synchronized firing
- The difference between neural trajectories in fig 4
- The decoding in figure 5 : can pre- and post-sound licks simply be discriminated based on the fact that the licks are more vigorous/synchronous/numerous?

We appreciate the reviewer's feedback on the need for appropriate controls for motor activity, and we fully agree that this is crucial. To address this concern and determine whether the observed changes in neural activity in the auditory cortex across sessions are driven by variations in motor behavior (such as changes in licking patterns) or are decision-related, we implemented the reviewer's suggestions in the following ways:

(1) We characterized the dynamics of licking and compared them across different conditions and bins.
(2) We repeated all our main analyses—including changes in firing rate (Fig 2), levels of synchrony in neural activity (original Fig 3), differences in neural trajectories (original Fig 4), and decoding accuracy (original Fig 5)—using lick-matched trials.

Further details on these analyses are provided below:

(1) <u>Lick dynamics</u>

As a first step, we examined the lick dynamics. We calculated the lick probability after the first lick for each bin and trial outcome. As expected, we observed differences in the lick patterns based on the timing of water delivery (**new Fig. 1b**). More interestingly, we found that as the animal gained experience with the task, there were notable changes in lick probability: the probability of the animals licking again after the first lick decreased.

When we focused on the 165 ms time window we used for our neural activity analysis in the manuscript, we also observed here a general decrease in the lick probability following the first lick as mice gained experience with the task (**new Appendix Figure**

S4a, 2-way Anova, Bin: F=142.9 p= $1.4e^{-148}$). This decrease appears to be primarily due to the reduced lick probability after the first bin, which coincides with the mice starting to learn the rules of their new environment and understanding when licks would lead to water. Notably, lick probability in Bins 1 and 6 differed significantly from the other bins, with mice licking significantly more in Bin 1 and less in Bin 6 compared to the rest (post hoc p<0.01 for all comparisons between Bin 1 and 6 and the remaining bins, Bonferroni corrected for both original and down-sampled data).

Overall, these results demonstrate that as mice learned the task rules, their licking behavior adapted significantly. We observed notable changes in lick probability and patterns, reflecting their growing experience with the task and the timing of water delivery.

However, licking is not necessarily an all-or-nothing event; mice can exhibit a range of licking patterns, including prolonged bursts, brief episodes, and everything in between and the preparation for longer or shorter motor activities could influence neural activity. Therefore, we also examined the lick burst length across different bins, both in the pre- and post-sound periods, and for the different behavioral outcomes (**new Fig. 1h**). As detailed in the manuscript, our primary focus is on the activity surrounding the first lick, so we specifically analyzed the length of the lick burst following the first lick.

When analyzing the lick burst durations, we observed a significant change in the lick bursts' length between the first bin (both in the pre- and post-sound periods) and the remaining bins (**new Fig. 1h**, 2-way Anova Bin: F=64.3 p= $1.1e^{-66}$; down-sampled F=61.5 p=$1.2e^{-63}$; post-hoc p<0.01 for all comparisons with Bin 1). This difference was influenced by the division between train bins and test bins (Fig. 1h, 2-way Anova Bin x time interaction F=8.6 p=$3.7e^{-08}$). Lick bursts were shorter in Bins 4-6 compared to Bins 1-3 (post hoc p<0.05 for all comparisons, Bonferroni corrected). Additionally, lick bursts in the post-sound period were generally longer than those in the pre-sound period (**new Fig. 1h**, 2-way Anova time: F=109 p=1.6 e^{-25}). This suggests that in the initial bins, lick timing was less crucial, mice were less familiar with the task rules and the timing of water delivery, resulting in longer lick bursts. As the mice gained experience, their lick bursts became shorter and more efficient.

Interestingly, lick burst length in hit trials during the post-sound period exhibited a bimodal distribution. In most trials, mice refrained from additional licking after their initial post-sound lick. However, in some trials, mice continued licking until water delivery.

We repeated this analysis using down-sampled lick data, reducing the sampling rate from 500 Hz to 30 Hz to match the neural data sampling rate. In both cases, we observed changes in lick patterns as the animal became more experienced with the task (**New Supp. Fig.1b**, lick burst length; 2-way Anova down-sampled F=122 p=1.9 e^{-28}, Bin x time interaction F=7.3 p=$6.2e^{-07}$). As mice learned the task rules, their licking

behavior adapted accordingly. We observed notable changes in lick probability and patterns, reflecting their growing experience with the task and the timing of water delivery. Specifically, in Bin 1, when the mice were less experienced, they licked more indiscriminately. In contrast, by Bin 6, with increased experience, their licking became more efficient and targeted.

Together, these analyses demonstrate that, as the reviewer suggested, mice modify their lick patterns based on their experience and the task rules.

(2) Lick matched analyses

To determine if these changes in licking dynamics affect neural activity in the auditory cortex and explain the changes in activity we found as the mice gain experience with the tasks, we repeated all our mean analyses in lick-matched trials as the reviewer suggested. This included examining firing rate changes (Fig. 2), variations in levels of synchrony (now Fig. 4), differences in neural trajectories (new Fig. 5), and decoding accuracy (Fig. 6) with selected subsets of trials with matched lick burst length.

We categorized the trials into three groups: short, intermediate, and long lick bursts. This categorization was performed twice - once using the down-sampled lick data for consistency with our neural data, and once using the original lick data for more precision and higher temporal resolution. A licking burst was defined as two or more consecutive licks with pauses greater than 500 ms (Johnson, A. W. et al. 2010, Boughther Jr et al 2006). The 500 ms threshold helped us to distinguish between bursts of licking (clusters of licks with short intervals between them) and pauses between bursts. We categorized lick bursts based on their duration as follows: short lick bursts ($0.002 \le x < 0.15$ seconds), intermediate lick bursts ($0.15 \le x < 0.5$ seconds), and long lick bursts ($0.5 \le x < 1$ second). For the down-sampled lick data, the categories were adjusted to short lick bursts ($0.033 \le x < 0.2$ seconds), intermediate lick bursts ($0.2 \le x < 0.5$ seconds), and long lick bursts ($0.5 \le x < 1.33$ seconds). Bursts longer than 1 second (or 1.33 seconds for the down-sampled data) were excluded from this analysis due to insufficient trial numbers across all bin-trial outcome combinations.

The results of the repeated analysis with lick-matched trials are as follows:

Changes in activity: as in Figure 2, in all lick-burst matched groups, both enhanced and suppressed cells demonstrated increased absolute peak activity around the lick in the post-sound period when compared to the pre-sound period (**New Supp, Fig. 4b & c**, 2-way ANOVA, short burst $F=41.43$ $p=1.4\ e^{-10}$ and $F=29.62$ $p=6.21e^{-08}$ accordingly, intermediate $F=45.3$ $p=2.06e^{-11}$ and $F=28.1$ $p=1.28e^{-07}$ accordingly, long $F=8.19$ $p=0.004$ and $F=9.01$ $p=0.0027$ accordingly & defined by down-sampled lick data: short burst $F=31.16$ $p=2.6\ e^{-08}$ and $F=11.9$ $p=0.0006$ accordingly, intermediate $F=32.7$ $p=1.19$

e-08 and F=30.9 $p=3.2e^{-08}$ accordingly, long F=15.1 p=0.0001 and F=15.5 p=0.0001 accordingly). We also found an increase in enhanced cells peak activity as the mice gain experience with the task.

As in Fig. 2g-h, we also found in all lick-burst matched groups a decrease in activity during both pre-lick and post-lick periods as mice gained task experience (**New Appendix Figure S5a**). The more pronounced reduction in pre-lick activity (solid line) led to increased deltas of activity between the post and pre-lick periods for enhanced cells (**New Appendix Figure S5b**, 2-way ANOVA, cell types x bin interaction short burst F=11.21, $p=3.7\ e^{-19}$, intermediate F=8.25 $p=2.2\ e^{-13}$, long F=3.76 ,$P=4.6\ e^{-05}$). Importantly, we also observed in all lick-burst matched groups a resurgence to novice activity levels during the transition from training to testing.

These analyses suggest that the described changes in activity are not being driven by changes in licking activity.

<u>Correlation analysis</u>: To test this further, we also repeated our correlation analysis. As with the original analysis (now Fig. 4a) we consistently observed higher noise correlations for enhanced and suppressed cell groups during the lick window (**New Appendix Figure S6a**, 2-way ANOVA, enhanced cells short burst F=217.7, $p=6.6\ e^{-49}$, intermediate F=321 $p=3.6\ e^{-71}$, long F=206 ,$P=5.2\ e^{-58}$, suppressed cells short burst F=178, $p=1.6\ e^{-40}$, intermediate F=197 $p=1.1\ e^{-44}$, long F=144 ,$P=3.8\ e^{-33}$).

<u>Neural trajectories-Discriminability analysis</u>: We also repeated our new discriminability analysis (please see the answer to reviewer 2 on this topic below) with licked-matched trials and observed the same phenomenon: an increased Euclidean distance between Hit and LL trajectories in Bins 5 and 6 when compared to Bin 4 (**New Appendix Figure S7a**, t-test Bin 4 vs Bin 5 p=1.4 e-104 and Bin 4 vs Bin 6 p=5 e-74).

<u>Classification analysis</u>: We also repeated our analysis to determine if the observed licks could be accurately classified as occurring during the pre-sound or post-sound period solely by the activity in the ACtx (Fig. 5). Similarly to our previous results, we found that the enhanced cell group had higher prediction accuracy when testing all groups (**New Appendix Figure S8**, long, short & intermediate lick bursts, Permutation tests, p<0.0001), suggesting that changes in lick dynamics are not driving the phenomenon.

The repetition of our main analyses with lick-matched trials argues against changes in lick dynamics being the main force driving the observed modulation of activity in the auditory cortex and supports the nonmotor nature of the decision or decision output in A1. These new analyses suggest that motor or behavioral decision signals in the auditory cortex may be gated by their auditory task relevance, as the reviewer suggested. Moreover, this modulation of cortical activity seems to be refined with experience.

We believe we have addressed the reviewer's concern by thoroughly analyzing lick dynamics and matching trials by burst lengths to control for motor activity. Our findings indicate that as mice gain experience with the task, their licking behavior significantly adapts, with notable changes in lick probability, burst length, and response patterns based on trial outcomes. This adaptation reflects the mice's growing familiarity with the task and the timing of water delivery. Crucially, our results demonstrate that these changes in licking dynamics do not account for the observed alterations in auditory cortex activity across sessions, as similar neural patterns were found when lick bursts were matched across conditions. Instead, our findings point to motor signals representing a behavioral choice, being gated by their auditory task relevance.

We are grateful to the reviewer for highlighting this important issue. These additional analyses have refined and strengthened our study, making it more focused and innovative.

Training phase : The authors highlight that during the training phase, mice don't need to attend to the sound to get water but nonetheless the mice do seem to use the sound to sculpt their behavior during this phase. The authors suggest that during this period what is going on is mainly familiarization with the environment etc but clearly figure 1 shows that mice are using the sound to modify their behavior since they increase the probability of post-sound licks and decrease the miss trials. It would be interesting to know whether the animals are also already waiting to lick (since water only comes after 3s) during this phase. This would clarify the nature of the change in behavior between the training and testing phase.

We agree with the reviewer that the mice are using the sound to modify their behavior, we didn't mean to imply otherwise. Our point was that other behavioral strategies could be at play as well. To explore this point further, we followed the reviewer's suggestion and analyzed the time of lick post sound during the training period. There is indeed a significant shift in the timing of the first lick (**New Supp. Fig.1a**,1-way Anova $F=14.46$ $p=2.023e^{-07}$).

This result, combined with our previous analyses (the increase in post-sound licking shown in Fig. 1d-e, and changes in neural activity starting as early as Bin 2 shown in Fig. 2), supports the hypothesis that mice are indeed using the sound during the training phase and adjusting their behavior accordingly. However, we cannot entirely rule out the possibility that the mice might be employing other strategies at certain times.

We made this point clearer in the result and discussion section.

Relation to literature : Literature on "innnate", non task related motor activity in the cortex and its association with sensory responses is not well-cited and should be used to contextualize the reports findings (ex: Bimbard et al, 2023, Lohuis et al, 2024). This is important since the authors report essentially no lick responses outside the context of the task. In particular the studies by Schneider et al are cited but the fact that they show activity changes related to motor activity per se, at odds with the current study, should be clearly discussed and reasons for discrepancies explained.

Our study does not include measurements from purely non-task conditions. However, we can consider Bin 1, where the mice have no prior task experience along with the licking activity that occurs before the sound, as representative of "innate" or non-task-specific motor activity. In our original manuscript, we examined neural activity in these contexts and demonstrated the presence of lick-evoked activity before the sound, consistent with previous studies. Our aim was to emphasize that this pre-sound lick-related activity is not modulated by experience, unlike the activity associated with licks that occur after the sound. We acknowledge that this distinction may not have been clearly articulated, so we provide further clarification below.

We show that in the initial Bin (Fig. 2d), lick-evoked activity is present, consistent with recent literature. Furthermore, as the mice gain more experience, lick-evoked activity persists in the pre-sound period (e.g., Fig. 2d, light colors, Fig. 2e, light colors**, New Appendix Figure S3a & b, New Appendix Figure S4b & c** light colors and **New Appendix Figure S5a**). These observations align with prior studies showing that motor-related modulation of individual neurons is highly variable within and across experimental paradigms. For example, Schneider et al. (2014), Zhou et al. (2014), and Bigelow et al. (2019) reported a general suppression of spontaneous and sound-evoked activity, while Henschke et al. (2021) and Vivaldo et al. (2022) documented enhanced responses.

The consensus in the literature is that motor-related activity effects in auditory areas are heterogeneous. For instance, Morandel et al. observed variable effects of locomotion on sound-evoked responses and spontaneous activity at the single-cell level, showing both suppression and enhancement. On average, spontaneous fluorescence was enhanced during running, walking, postural adjustments, and sniffing, while neural responses were suppressed during grooming. Similarly, we observed a heterogeneous effect in our study, with some cells showing enhancement, suppression, or no change in activity due to licking.

Our findings extend this body of work by showing that lick-evoked activity in the auditory cortex following the sound is influenced by experience and task rules, whereas pre-sound lick-evoked activity is less affected. Although overall A1 activity decreases as the sessions progress, the relative activity during licking remains elevated (or decreased for

suppressed cells) compared to the pre-lick period. These results align with literature demonstrating that state changes and behavioral factors impact sensory cortex activity (e.g., Vinck et al., 2015; Neil et al., 2010; Bimbard et al., 2023; Lohuis et al., 2024, as the reviewer noted). Our results further suggest that, in our task, sound serves as a gate for modulating lick-evoked activity, with this effect becoming more pronounced as mice gain experience. We have made this point clearer in the result and discussion sections and cited the corresponding literature.

The authors discuss the importance of separating in time the stimulus, the decision and the motor action to disambiguate between them and they indeed provide a task that allows for this. However since they mainly analyse lick responses it's not clear what is being separated. Could the authors clarify their strategy given the actual behavior in the task and how this relates to other studies?

Our study builds upon two key areas of research that have explored auditory cortex activity in task engagement and training contexts.

First, prior studies have demonstrated that stimulus-evoked activity in the auditory cortex varies significantly when a mouse is actively engaged in a task compared to passive listening. This activity also evolves with experience (e.g., Blake et al., 2002; Paraouty et al., 2023; Recanzone et al., 1993; Polley et al., 2006, among others). These works primarily focus on changes in sound-evoked responses.

Second, a separate line of research has shown that, once a task is mastered, the auditory cortex encodes not only the sound identity but also the animal’s choice and anticipated reward size (e.g., Guo et al., 2019; Rodgers et al., 2014). However, these studies often examine animals at expert stages, leaving open interesting questions aburst the development of task-related activity in the auditory cortex: Is it an innate response, or does it evolve with experience? Additionally, some studies struggle to distinguish between task-related and movement-related neural activity, frequently comparing active periods against passive ones (e.g., Guo et al., 2019).

Our aim was to build on these two lines of work by investigating whether task-dependent or decision-related activity in the auditory cortex (distinct from sensory representation) evolves as mice gain experience. Our findings show that activity in specific sub-populations of neurons changes as mice gain experience with the task. This was particularly apparent within the ‘enhanced’ cell subgroup, whose activity is modulated by task rules and experience.

We acknowledge the challenge of disentangling sensory, motor, and experience-driven plasticity, as these processes are deeply intertwined. To address this, we designed a task that temporally separates sound-evoked activity from the decision phase.

Specifically, in delayed-response tasks, decision-making can occur immediately after the sound, during the delay, or just before the motor action (licking). Therefore, our emphasis stayed on the activity surrounding the behavioral output of the decision – specifically, the lick onset. At this juncture, the animal has made its decision, allowing us to examine the modulation of cortical activity during the behavioral output phase of that decision.

Our approach allows us to differentiate licks driven by different decision intentions and compare their associated neural activity. By integrating the original analysis with the additional analyses recommended by the reviewer, we demonstrate that the observed changes in auditory cortex activity are not merely due to differences in lick rates. Instead, they reflect evolving decision-related processing as the mice gain task experience.

Neural response interpretation : The authors should describe any global changes in neural activity level that could accompany task engagement and learning (this has been reported in many studies : Otazu et al, 20019; Rodgers et al, 2014; Bagur et al, 2018). Such changes can lead to apparent changes in lick responsiveness driven by lower baselines for example. Spontaneous activity can be hard to evaluate in calcium imaging but sound responses could be used for comparison for example.

Learning-related global effects on spontaneous and stimulus-evoked activity in auditory areas are heterogeneous across studies. For example, Bagur *et al*. (2018) reported that spontaneous activity before stimulus presentation increased in engaged conditions, while baseline-corrected stimulus-evoked activity remained unchanged when comparing passive listening to active discrimination sessions. In contrast, Otazu *et al*. (2009) found that engagement in an auditory task suppressed stimulus-evoked responses in the auditory cortex without affecting the preceding spontaneous activity. Scott *et al*. (2007) observed an elevation in spontaneous discharge during behavior, while Buran *et al*. (2014) noted a 26% reduction in spontaneous activity during task engagement.

Most of these studies compare passive vs engaged conditions. We don't have such a comparison in our paradigm since we didn't do passive trials in the training or testing phases. In our original submission, we examined the activity before the lick and observed a decrease in activity (original Fig. 2i, now Fig. 2g). As discussed in more detail below, in the response to the reviewer's question regarding Fig. 2e and 2i (now Fig. 2g), both pre-lick and post-lick activity decline as training progresses. However, the more pronounced reduction in pre-lick activity results in a relative increase in lick-evoked activity as the mice become more experienced with the task. So, we agree with the reviewer that the combination of global and stimulus/action-evoked changes in activity is important to understand how the auditory cortex relays information to

downstream areas. That is why we examined the activity both before and after the lick in the pre-sound and post-sound periods.

To further explore this point, we analyzed the spontaneous activity during the half-second before the sound's onset for each trial using deconvolved data, along with the tone evoked-activity with and without baseline correction. We found a significant change in the tone evoked activity (**new Fig. 3**, 1-way Anova F=211.8 P=3.6 e^{-147}), characterized by an increase in activity during the first four bins, peaking at bin 4 (post-hoc, bin1 vs bins 3 and 4 p<0.05, Bonferroni corrected), followed by a reduction in activity (post-hoc, bin4 vs bins 5 and 6 p<0.05, Bonferroni corrected). Additionally, we found a small decrease in spontaneous activity as the bins progressed (1-way Anova F=16.4 P=1.7e^{-15}). The bigger change in tone evoked activity compared to spontaneous activity led to small changes when analyzing tone-evoked activity with baseline correction. We again found a significant increase peaking at bin 4 (1-way Anova F=636 P=3.9 e^{-286}).

These results resemble findings from Blake et al., 2002, where training 2 monkeys in an auditory task led to an increase in activity to the target tone until the animal achieved a behavioral "break point". Beyond this point, auditory cortex activity declined but remained higher for the target tone compared to a non-target tone. In our study, tone-evoked activity peaks at bin 4, coinciding with when the timing of sound becomes crucial.

Together, these results suggest that although global changes in spontaneous activity are present, they do not strongly affect tone-evoked responses. In contrast, analysis of neural activity around licks revealed that the greater reduction in pre-lick activity with increased task experience leads to a relative enhancement in neural responses following the lick."

We explained this point further in the result and discussion sections.

Sensory properties : The authors comment that the lick reactive cells are non-sensory, could they provide a figure describing this since? I understand this is not the main point of the paper but the relation between lick responses and sound responses will be of interest to many people?

While this is not the primary focus of our study, we agree that it is an interesting point, so we show in Appendix Figure S2c that the percentage of sound-responsive cells within the "none" group aligns with what is expected in the general population. However, this percentage is notably lower for the enhanced and suppressed cell groups. To further investigate this, we calculated the average Euclidean distance between sound-responsive cells and each of the enhanced, suppressed, and none cell groups. Our

analysis revealed no significant differences in distance across these groups (**New Appendix Figure S2d**).

We have also described the changes in tone-evoked activity and spontaneous activity as mentioned above (**new Fig. 3**).

Minor points :

- Could the authors add a time bar to fig 1a? – Of course, we added a time bar of 0.5 sec.

- Could they clarify between what types of trials they calculate the dprime in figure1

For Figure 1f, d' was calculated as d' = z(H) - z(F). Where H is the hit rate, and F is the false alarm rate calculated from the catch trials. We extended the explanation of the catch trials to the result section and methods section.

- Could the authors clarify the factors that were added to the decoder in the lower part of fig 5b? - We added a clarification in the methods section, lines 888-890: "In Fig 5. Bottom, we added to the decoder information about behavioral measures per session: the percentage of trials with licks (as presented in Fig. 1d) and the percentage of modulated cells (as presented Fig. 2f)."

- Lines 413-416 : the authors link their findings that mainly enhanced cells encode pre vs post licks and link this to the paper by Francis et al. I am not sure the link they make is clear here since the "small neuronal networks" referred to by this paper correspond to sparsely connected small sets of cells. Could they clarify what they meant?

Francis et al. showed that small neural networks in the auditory cortex were enough to predict behavioral choice. Similarly, our findings indicate that the activity of a small subgroup of neurons, specifically enhanced cells, is sufficient to predict or encode features of the animal's behavioral choice or output. To avoid any misunderstanding, we took the comparison to Francis et al. out of the discussion.

- I am pretty sure the authors are not tracking the same neurons across days, could they just clarify this in the methods ? - We added a clarification in the methods section lines 763:764 "Each session we returned to the same area, guided by the blood vasculature, but not necessarily the same cells."

- Figures 2e and 2i show contradictory results regarding the overall trend for enhanced and suppressed cells (change vs no change). It seems that this may be due to the fact that one analysis is done on z-scored activity and not the other (as far as I could gather

from the legends that are not clear on this point). Given that the peaks become narrower in time in d, if the traces are independently z-scored this will also enhance their height. Could the authors be clearer on what is quantified here?

The reviewer is correct that the difference is the z-score. The difference is that in Figure 2e, the data is normalized (z-scored), while the analysis in Figure 2i-j (now Fig. 2g-h) is not normalized. As the reviewer pointed out correctly, the peaks become higher and narrower with experience, with a setback in bin 4 where the task rules change, resulting in a lower peak. We acknowledge that this was not clearly explained. We now explain this better below and in the manuscript.

To normalize the data, we first z-scored the average activity from a second before the lick-onset to half a second after the lick onset (across trials) for each cell in each Bin as presented in Fig. 2d.

We then calculated the peak activity, from the normalized data, in the period closely surrounding the lick (from 165 ms before to 165 ms after the lick onset) as presented in Fig. 2e. The peak could be before or after the lick onset. As the sessions progressed, we observed an increase in absolute peak activity.

In Figure 2I (new Fig. 2g), we compare the average activity during the 165 ms before the lick (solid line, not z-scored) and the average activity during the 165 ms following the lick (dash line). Here, we found a reduction in both pre- and post-lick activity during the first three bins. However, the more pronounced reduction in pre-lick activity (solid line) led to increased deltas of activity between the post- and pre-lick periods for enhanced cells (Figure 2h). This explains why, when examining the z-scored data in Figure 2e, we observed an increase in peak activity: the larger difference in activity between the pre- and post-lick periods translates to higher peaks when the activity is z-scored per cell.

In Bin 4, when task rules changed, there was a notable increase in both pre- and post-lick activities. Since the pre-lick activity increase was more pronounced, it resulted in a decrease in the proportional change in activity (Fig. 2h) and a reduction in the peak of the z-scored data (Fig. 2d-e).

These findings indicate a general reduction in activity surrounding the lick as the sessions progress, with the relationship between pre- and post-lick activities being crucial for interpreting how the auditory cortex processes task-related neural activity. Notably, regardless of whether the data is normalized, we found that the changes in activity are experience-dependent, with significant shifts or resets in the activity of enhanced cells when task rules are altered, especially in the post-sound period.

We have updated the text, figure legends, and graph axes to better communicate these findings.

<u>Referee #2</u>:

Description: This is an interesting paper that adds new evidence aburst experience-driven plasticity in the auditory cortex in a mouse model. The paper has a good experimental design that enables the differentiation of decision-making-related, reward-related, and motor-related neural activity from those that could be modified by experience. The authors found that as mice gain more experience with the auditory task, there were more enhanced neurons, whose activity was enhanced by lick behavior. Further, the performance of decoders designed to differentiate the neural encoding of lick-behavior occurred with/without behavioral outputs also depends on the experience of mice. To sum up, the paper provided evidence that experience-driven modifications on decision-making representations of A1 neurons in a mice model. Our comments below are meant to improve this manuscript:

Major concerns:
1. The strongest evidence demonstrating that plasticity in A1 is sensory-experience-driven instead of motor-driven is comparing post-sound and pre-sound licks in experienced mouse. Have you considered how the large trial imbalance between pre-sound and post-sound licks affects the other analyses except the decoding? It will be helpful to includes the percentage of pre-sound licks for bin 4-6 in Figure 1g.

We appreciate the reviewer's feedback on the need for appropriate controls for motor activity, and we fully agree that this is crucial. To address this concern and determine whether the observed changes in neural activity in the auditory cortex across sessions are driven by variations in motor behavior (such as changes in licking patterns) or are decision-related, we implemented the reviewer's suggestions in the following ways:

(1) We included pre-sound Lick percentages in Figure 1.
(2) We characterized the dynamics of licking and compared them across different conditions and bins.
(3) We repeated all our main analyses—including changes in firing rate (Fig 2), levels of synchrony in neural activity (now Fig 4), differences in neural trajectories (now Fig 5), and decoding accuracy (now Fig 6)—using lick-matched trials.

Further details on these analyses are provided below:

(1) <u>Pre-sound percentages</u>

In Figure 1g, we show the performance rate for the different behavioral outputs during testing; in trials that the mice licked after the sound. The 3 options together make 100 percent of trials that the mice licked after the sound. So, it might be more informative to

add the percentage of trials with licks in the pre-sound period in Figure 1d, where we can compare in all the bins the percentage of trials where there was a lick in the pre-or post-sound periods. Here, the sum of the bins is not 100 percent since there were some trials that the mice licked both in the pre- and post-sound periods. When we look at all the bins together, we can see a decline in the percentages of trials where there is a pre-sound lick and an increase in the percentages of trials where there is a post-sound lick (2-way ANOVA for Bins 1-3, bin x period interaction $F=8.38$ $p=7e^{-04}$ and for Bins 1-6 bin x period interaction $F=5.8$ $p=0.0001$). We can also see in bin 4, the small reduction in post-sound licks when we changed the rules.

(2) <u>Lick dynamics</u>

As a first step, we examined the lick dynamics. We calculated the lick probability after the first lick for each bin and trial outcome. As expected, we observed differences in the lick patterns based on the timing of water delivery (**new Fig. 1b**). More interestingly, we found that as the animal gained experience with the task, there were notable changes in lick probability: the probability of the animals licking again after the first lick decreased.

When we focused on the 165 ms time window we used for our neural activity analysis in the manuscript, we also observed here a general decrease in the lick probability following the first lick as mice gained experience with the task (**new Appendix Figure S4a**, 2-way Anova, Bin: $F=142.9$ $p= 1.4e^{-148}$). This decrease appears to be primarily due to the reduced lick probability after the first bin, which coincides with the mice starting to learn the rules of their new environment and understanding when licks would lead to water. Notably, lick probability in Bins 1 and 6 differed significantly from the other bins, with mice licking significantly more in Bin 1 and less in Bin 6 compared to the rest (post hoc $p<0.01$ for all comparisons between Bin 1 and 6 and the remaining bins, Bonferroni corrected).

Overall, these results demonstrate that as mice learned the task rules, their licking behavior adapted significantly. We observed notable changes in lick probability and patterns, reflecting their growing experience with the task and the timing of water delivery.

However, licking is not necessarily an all-or-nothing event; mice can exhibit a range of licking patterns, including prolonged bursts, brief episodes, and everything in between and the preparation for longer or shorter motor activities could influence neural activity. Therefore, we also examined the lick burst length across different bins, both in the pre- and post-sound periods, and for the different behavioral outcomes (**new Fig. 1h**). As detailed in the manuscript, our primary focus is on the activity surrounding the first lick, so we specifically analyzed the length of the lick burst following the first lick.

When analyzing the lick burst durations, we observed a significant change in the lick bursts' length between the first bin (both in the pre- and post-sound periods) and the remaining bins (**new Fig. 1h**, 2-way Anova Bin: F=64.3 p= 1.1e^{-66}; down-sampled F=61.5 p=1.2e^{-63}; post-hoc p<0.01 for all comparisons with Bin 1). This difference was influenced by the division between train bins and test bins (Fig. 1h, 2-way Anova Bin x time interaction F=8.6 p=3.7e^{-08}). Lick bursts were shorter in Bins 4-6 compared to Bins 1-3 (post hoc p<0.05 for all comparisons, Bonferroni corrected). Additionally, lick bursts in the post-sound period were generally longer than those in the pre-sound period (**new Fig. 1h**, 2-way Anova time: F=109 p=1.6 e^{-25}). This suggests that in the initial bins, lick timing was less crucial, mice were less familiar with the task rules and the timing of water delivery, resulting in longer lick bursts. As the mice gained experience, their lick bursts became shorter and more efficient.

Interestingly, lick burst length in hit trials during the post-sound period exhibited a bimodal distribution. In most trials, mice refrained from additional licking after their initial post-sound lick. However, in some trials, mice continued licking until water delivery.

We repeated this analysis using down-sampled lick data, reducing the sampling rate from 500 Hz to 30 Hz to match the neural data sampling rate. In both cases, we observed changes in lick patterns as the animal became more experienced with the task (**New Supp. Fig.1b**, lick burst length; 2-way Anova down-sampled F=122 p=1.9 e^{-28}, Bin x time interaction F=7.3 p=6.2e^{-07}). As mice learned the task rules, their licking behavior adapted accordingly. We observed notable changes in lick probability and patterns, reflecting their growing experience with the task and the timing of water delivery. Specifically, in Bin 1, when the mice were less experienced, they licked more indiscriminately. In contrast, by Bin 6, with increased experience, their licking became more efficient and targeted.

(3) Lick matched analyses

To test if these changes in licking dynamics affect neural activity in the auditory cortex and explain the changes in activity we found as the mice gain experience with the tasks we repeated all the mean analyses, including the changes in firing rate (Fig. 2), different levels of synchrony of activity (now Fig. 4), differences in between neural trajectories (now Fig. 5) and decoding accuracy (now Fig. 6) with selected subsets of trials with matched lick burst length.

We categorized the trials into three groups: short, intermediate, and long lick bursts. This categorization was performed twice - once using the down-sampled lick data for consistency with our neural data, and once using the original lick data for more precision and higher temporal resolution. A licking burst was defined as two or more consecutive licks with pauses greater than 500 ms (Johnson, A. W. et al. 2010, Boughther Jr et al 2006). The 500 ms threshold helped us to distinguish between bursts

of licking (clusters of licks with short intervals between them) and pauses between bursts. We categorized lick bursts based on their duration as follows: short lick bursts ($0.002 \leq x < 0.15$ seconds), intermediate lick bursts ($0.15 \leq x < 0.5$ seconds), and long lick bursts ($0.5 \leq x < 1$ second). For the down-sampled lick data, the categories were adjusted to short lick bursts ($0.033 \leq x < 0.2$ seconds), intermediate lick bursts ($0.2 \leq x < 0.5$ seconds), and long lick bursts ($0.5 \leq x < 1.33$ seconds). Bursts longer than 1 second (or 1.33 seconds for the down-sampled data) were excluded from this analysis due to insufficient trial numbers across all bin-trial outcome combinations.

The results of the repeated analysis with lick-matched trials are as follows:

Changes in activity: as in Figure 2, in all lick-burst matched groups, both enhanced and suppressed cells demonstrated increased absolute peak activity around the lick in the post-sound period when compared to the pre-sound period (**New Appendix Figure. 4b & c**, 2-way ANOVA, short burst $F=41.43$ $p=1.4\ e^{-10}$ and $F=29.62$ $p=6.21e^{-08}$ accordingly, intermediate $F=45.3$ $p=2.06e^{-11}$ and $F=28.1$ $p=1.28e^{-07}$ accordingly, long $F=8.19$ $p=0.004$ and $F=9.01$ $p=0.0027$ accordingly & defined by down-sampled lick data: short burst $F=31.16$ $p=2.6\ e^{-08}$ and $F=11.9$ $p=0.0006$ accordingly, intermediate $F=32.7$ $p=1.19$ e-08 and $F=30.9$ $p=3.2e^{-08}$ accordingly, long $F=15.1$ $p=0.0001$ and $F=15.5$ $p=0.0001$ accordingly). We also found an increase in enhanced cells peak activity as the mice gain experience with the task.

As in Fig. 2g-h, we also found in all lick-burst matched groups a decrease in activity during both pre-lick and post-lick periods as mice gained task experience (**New Appendix Figure S5a**). The more pronounced reduction in pre-lick activity (solid line) led to increased deltas of activity between the post and pre-lick periods for enhanced cells (**New Appendix Figure S5b**, 2-way ANOVA, cell types x bin interaction short burst $F=11.21$, $p=3.7\ e^{-19}$, intermediate $F=8.25$ $p=2.2\ e^{-13}$, long $F=3.76$,$P=4.6\ e^{-05}$). Importantly, we also found in all lick-burst matched groups a resurgence to novice activity levels during the transition from training to testing.

These analyses suggest that the described changes in activity are not being mainly driven by changes in licking activity.

Correlation analysis: To test this further, we also repeated our correlation analysis to test if our results are driven by lick dynamics. As with the original analysis (now Fig. 4a) we consistently observed higher noise correlations for enhanced and suppressed cell groups during the lick window (**New Appendix Figure S6a**, 2-way ANOVA, enhanced cells short burst $F=217.7$, $p=6.6\ e^{-49}$, intermediate $F=321$ $p=3.6\ e^{-71}$, long $F=206$,$P=5.2\ e^{-58}$, suppressed cells short burst $F=178$, $p=1.6\ e^{-40}$, intermediate $F=197$ $p=1.1\ e^{-44}$, long $F=144$,$P=3.8\ e^{-33}$).

Neural trajectories-Discriminability analysis: We also repeated our new discriminability analysis (please see on this topic below) with licked-matched trials and observed the same phenomenon: an increased Euclidean distance between Hit and LL trajectories in Bins 5 and 6 when compared to Bin 4 (**New Appendix Figure S7a**, t-test Bin 4 vs Bin 5 p=1.4 e-104 and Bin 4 vs Bin 6 p=5 e-74).

Classification analysis: We also repeated our analysis to determine if the observed licks could be accurately classified as occurring during the pre-sound or post-sound period solely by the activity in the ACtx (now Fig. 6). Similarly to our previous results, we found that in lick-matched trials the enhanced cell group had higher prediction accuracy when testing all groups (**New Appendix Figure S8**, long, short & intermediate lick bursts, Permutation tests, p<0.0001), suggesting that changes in lick dynamics are not driving the phenomenon.

The repetition of our main analyses with lick-matched trials argues against changes in lick dynamics being the main force driving the observed modulation of activity in the auditory cortex and supports the nonmotor nature of the decision or decision output in A1. These new analyses suggest that motor or behavioral decision signals in the auditory cortex may be gated by their auditory task relevance, and this modulation of cortical activity is refined with experience.

2. From the Method section of dimensionality reduction analysis and Figure 4, it seems that the authors conducted their PCA analysis for data recorded in each bin separately and then calculated the Euclidian distances within each PCA space. We do not believe it is appropriate to compare Euclidean distances that were calculated in different PCA spaces. Even when you keep incorporate all of the PCs, it does not guarantee the Euclidean distance calculated in different spaces are at the same scale. Consequently, you might find different Euclidean distances when projecting the same pair of datapoints into different PCA spaces, although there is only one ground truth distance. We suggest the authors use distribution alignment, hierarchical Wasserstein alignment, or CCA to align different spaces before comparing Euclidean distances calculated in different spaces (see Dabagia, M., Kording, K.P. & Dyer, E.L. Aligning latent representations of neural activity. Nat. Biomed. Eng 7, 337-343 (2023). https://doi.org/10.1038/s41551-022-00962-7).

We thank the reviewer for highlighting this point. To address this, we completely re-did the analysis in Fig. 5 (originally named Fig.4). We used Canonical Correlation Analysis (CCA) to align the different neural spaces before comparing Euclidean distances. Specifically, we identified the linear transformations that maximally correlated the latent dynamics of Bin 5 and Bin 6 to those of Bin 4, projecting these transformations back into

the original data. This approach aims to compensate for neuron turnover and other changes in the recorded population. We then calculated Euclidean distances within the aligned space. The analyses were conducted as described below, with results presented in the **new Figure 5** and **new Appendix Figure S7**.

For this analysis, we included all concatenated trials for each bin. To construct the data matrices, we equalized the number of Hit and Late Lick trials within each session to ensure balanced comparisons. If the true latent dynamics across sessions were stable, the trajectories of the empirically estimated latent dynamics would remain similar after alignment, with leading canonical correlations (CCs) approaching one. Conversely, if there were no stable latent dynamics between sessions, the trajectories would differ significantly even after alignment, resulting in low CCs. Our results showed relatively high canonical correlations, suggesting similar latent dynamics across bins.

To provide context, we used the within-day variability in latent dynamics across blocks of trials in Bin 4 as an upper bound for CCs. We split all trials from one day into two nonoverlapping sets, matched by trial types, and performed CCA on the latent dynamics (500 repetitions). To establish a lower bound, we calculated the pairwise correlations between unaligned spaces.

We calculated Euclidean distances in the aligned space and found significant differences between Bins 4 and 5 and Bins 4 and 6 (t-test p=7.3e-99 and p=5.1e-33, respectively; $p<0.01$ permutation test). Further, when analyzing distances per cell type, we observed, consistent with our previous analyses, that the distance between Hit and Late Lick trials was greater for enhanced cells compared to suppressed and non-modulated cells. Importantly, we found that the increase in discriminability starts before the lick.

3. Fig. 2e&i,j & line197: How do the authors interpret their finding that neurons show decreased average spiking activity in the pre- and post-lick window but an increased absolute peak firing rate during the same time window? If it is because more cells that are spatially overlapped and firing more weakly individually, then wouldn't we also expect a decrease of the pair-wise distance within each cell group in the test bins relative to those in the training bins?

The key difference is that in Figure 2e, the data is normalized (z-scored), while the analysis in Figure 2i-j (now Fig. 2g-h) is not normalized. This was not clearly explained. We now explain this better below and in the manuscript.

To normalize the data, we first z-scored the average activity from a second before the lick-onset to half a second after the lick onset (across trials) for each cell and Bin as presented in Fig. 2d.

We then calculated the peak activity, from the normalized data, in the period closely surrounding the lick (from 165 ms before to 165 ms after the lick onset) as presented in Fig. 2e. The peak could be before or after the lick onset. As the sessions progressed, we observed an increase in absolute peak activity.

In Figure 2I (new Fig. 2g), we compare the average activity during the 165 ms before the lick (solid line, not z-scored) and the average activity during the 165 ms following the lick (dash line). Here, we found a reduction in both pre- and post-lick activity during the first three bins. However, the more pronounced reduction in pre-lick activity (solid line) led to increased deltas of activity between the post- and pre-lick periods for enhanced cells (Figure 2h). This explains why, when examining the z-scored data in Figure 2e, we observed an increase in peak activity: the larger difference in activity between the pre- and post-lick periods translates to higher peaks when the activity is z-scored per cell.

In Bin 4, when task rules changed, there was a notable increase in both pre- and post-lick activities. Since the pre-lick activity increase was more pronounced, it resulted in a decrease in the proportional change in activity (Fig. 2h) and a reduction in the peak of the z-scored data (Fig. 2d-e).

These findings indicate a general reduction in activity surrounding the lick as the sessions progress, with the relationship between pre- and post-lick activities being crucial for interpreting how the auditory cortex processes task-related neural activity. Notably, regardless of whether the data is normalized, we found that the changes in activity are experience-dependent, with significant shifts or resets in the activity of enhanced cells when task rules are altered, especially in the post-sound period.

We have updated the text, figure legends, and graph axes to better communicate these findings.

Also, to delineate the "purely" sensory -related and experience-driven plasticity, wouldn't an examination of the post-sound and pre-sound activity be a cleaner analysis? This removes any motor-related and experience-driven plasticity. Indeed, from our eye, it looks like there is also an induced effect for pre-sound licks in Fig. 2e and extended fig. 2.

Previous studies have shown that stimulus-evoked activity in the auditory cortex changes when a mouse engages in a task and evolves with experience. We aimed to investigate whether decision coding (non-sensory activity) in the auditory cortex is similarly influenced by task relevance and experience.

We acknowledge the reviewer’s point on the challenge of disentangling sensory, motor, and experience-driven plasticity, as these processes are deeply interconnected. To address this, we designed a paradigm that separates sound-evoked activity (i.e. “purely” sensory) from the decision phase.

In examining the activity surrounding the lick, we considered that in delayed-response tasks, the decision-making process could occur at various times: immediately after the sound, during the delay period, or just before the lick. Analyzing activity immediately after sound presentation would predominantly capture sensory responses, as the auditory cortex can continue to exhibit sound-evoked activity even after the sound has ended. Examining the delay period was another option; however, the timing of decision-making can vary across cells and mice, occurring at different points during this period. Therefore, we focused on the neural activity around the behavioral output of the decision—the onset of licking. At this point, the animal's decision has been made, allowing us to assess how cortical activity is modulated during the execution phase of the decision.

To control for motor effects, we conducted a detailed analysis of licking patterns and their impact on all our main analyses (see the previous response for details). While we observed changes in lick dynamics, they did not account for the experience-dependent changes observed in enhanced cells.

Additionally, to the reviewer's point, we analyzed the pre-sound activity during the half-second before the sound's onset, along with the tone activity with and without baseline correction to assess purely sensory responses. We found a significant change in the tone evoked activity (**new Fig. 3**, 1-way Anova $F=211.8$ $P=3.6\ e^{-147}$), characterized by an increase in activity during the first four bins, peaking at bin 4 (post-hoc, bin1 vs bins 3 and 4 $p<0.05$, Bonferroni corrected), followed by a reduction in activity (post-hoc, bin4 vs bins 5 and 6 $p<0.05$, Bonferroni corrected). Additionally, we found a small decrease in spontaneous activity as the bins progressed (1-way Anova $F=16.4$ $P=1.7e^{-15}$). The larger changes in tone-evoked activity compared to pre-sound activity resulted in minimal differences when baseline-corrected (1-way Anova $F=636$ $P=3.9\ e^{-286}$).

Together, these analyses, along with our previous results, demonstrate that multiple changes occur in Auditory cortex activity as mice gain task experience. Changes to "purely" sensory activity and changes to motor or behavioral choice signals.

4. Line 101-106: It is not clear the behavioral difference between a late lick and a miss trial. From a signal detection point of view, they would be the same.

We agree with the reviewer that, from a signal detection perspective, late lick and miss trials are similar. However, the key distinction lies in their motor components. Our objective was to compare neural activity in the auditory cortex between conditions where the mouse detected the sound and licked (hit or early lick) and conditions where the mouse did not detect the sound but still licked. This approach allows us to examine cortical activity associated with different behavioral and signal detection outcomes while maintaining similar motor actions across conditions.

To facilitate this comparison, we introduced a condition where a small amount of water was delivered if the mouse did not detect the sound and refrained from licking within the first 3 seconds, encouraging the mouse to lick afterward. This enabled us to compare trials where the mouse detected the sound and licked with trials where it failed to detect the sound or chose to wait for the small water reward but still performed a licking action.

In miss trials, the mouse neither detected the sound nor licked. We did not focus on these trials, as the absence of licking behavior would make comparisons with trials involving licking less meaningful. We have clarified this point in the manuscript.

Minor concerns:
The grey shading around lines in the figures makes it difficult to distinguish line colors. The trajectories plots need more details about meanings of dashed vs solid line, squares, stars, and circles. It would be more helpful if the authors showed 95% confidence intervals as error bars instead of SEMs. We improved the Figures to make them clearer, and we re-did the analysis for the original Fig.4 (with the trajectories), as suggested by the reviewer.

Line 128: Fig. 2g should be fig. 1g. – Yes, thank you, we corrected that accordingly.

Fig. 1a: Please clarify the duration of the lower water reward window. It was also 1.5 seconds. We added the information to the methods section.

Fig. 2h: Please provide a legend and better color choices for all lines. We find it hard to parse the different line colors. We made the lines thicker and added a legend next to them.

Fig. 4: Shouldn't the figure legend a comparison between late lick vs hit trials instead of miss vs hits trials? Yes, thank you for pointing this out. We fixed the legend.

Line 152: There is not a fig. 1h. – We fixed this reference to refer to figure 2b.

Line 162: Are all trials with licks before or after sounds used to delineate the characteristics of cells? Will the experience effects of enhanced cells be the same if you just use trials with pre-sound licks when delineating the cells' characteristics?

All trials with licks after the sound were used to classify cells as enhanced, suppressed, or non-modulated. Given that lick-induced activity occurs in the auditory cortex regardless of task engagement (for example - Clayton et al., 2021), we used licks

occurring in the pre-sound period as a control, where activity is primarily driven by motor output. Since licks in this period happen before the sound, there is no possibility of receiving a water reward, and we anticipated that this motor-driven activity would be less influenced by task experience.

While analyzing the data, we considered whether categorizing cells based on pre-sound activity would yield different results, as suggested by the reviewer. We conducted this analysis and reported it in the original extended data for Figure 2. In the initial manuscript, we stated: “To ascertain that this was a task-specific modulation of activity, we examined cell activity during licks in the pre-sound period, categorizing cells as enhanced, suppressed, or non-modulated (Extended data for Fig. 2). During the pre-sound period, the environment provides contextual information, such as the presence of the lick spout or the head-fixed condition, but no specific cues aburst the sound or potential water rewards. Therefore, we expected fewer task-dependent modulations. Indeed, while there was an overall decrease in activity, there was no significant increase in the ratio of pre- to post-lick activity as mice gained task experience for enhanced cells (Extended data for Fig. 2; 2-way ANOVA, cell types*bin interaction F=2.2, p=0.06; post-hoc, enhanced cells Bin1 vs Bin6 p=1, Bonferroni corrected), similar to Figures 2i-j.”

To further explore this, we examined cell characteristics and responses when cells were categorized by pre-sound period activity, resulting in a new Supplemental Figure 3.

This included:

Z-scored activity per bin as shown in Figure 2d: There was no significant effect of bin number (ANOVA F=1.7, p=0.1131), and Bin 1 activity was not significantly greater than that in Bins 3 or 6 for any cell type (p<0.05 for all post hoc tests, Bonferroni corrected).

Activity surrounding the lick as shown in Figure 2e: We observed no significant effect of bins on the activity patterns (ANOVA F=1.7, p=0.1131).

Comparison of noise correlations, as in Figure 4a: Noise correlations during the lick were higher than those before the lick across all cell types (2-way ANOVA: enhanced cells F=153.6, p=1.1e-34; suppressed cells F=65.2, p=9.1e-16; non-modulated cells F=1688, p<<0.01). However, the strength of noise correlations did not increase and, in some cases, decreased as mice gained task experience (Bin 1 vs Bin 6: enhanced p=1; suppressed p=1; none p<0.01).

Prediction accuracy by cell type activity, as in Figure 6: We did not find significant differences in prediction accuracy among enhanced, suppressed, and non-modulated cells (Permutation tests, p>0.05).

Overall, our findings indicate that although there is lick-induced activity in the pre-sound period, the influence of task experience on this activity is much weaker compared to the post-sound period as mice learn the relevance of licks.

Line 190-191: Please discuss in more detail how you spatially segregated the different cell groups. For each imaging session, we have the x and y locations of each cell (the z position was kept constant throughout the recording sessions). We calculated the Euclidean distance of each pair in the Enhanced, Suppressed, and None cell groups per session. Then, we averaged across sessions (lines 824:827).

Line 192: It may be helpful to provide a figure showing the spatial overlap between lick-responsive and sound-responsive cells.
To achieve this across sessions and mice, we would require a large number of maps. To simplify the presentation of the analysis, we calculated the average Euclidean distance between the sound-responsive cell and the enhanced, suppressed, and non-responsive cells – **new Appendix Figure S2d**. Our results showed no significant differences between these groups.

Line 220: Can the authors discuss the difference in the findings of bin4 in fig. 2i and fig. 2e. and Line 223: how the decrease of spike difference between pre- and post- lick period explains the decreased peak activity during these two lick windows?

The key difference is that in Figure 2e, the data is normalized (z-scored), while the analysis in Figures 2i-j (renamed 2g-h in the revised version) does not.

In Figure 2e, we z-scored the average activity (across trials) for each cell in each bin and calculated the peak activity. As the sessions progressed and the peaks became higher and narrower, we observed an increase in absolute peak activity.

In Figure 2i (now 2g), we compare the non-z-scored average activity during the 165 ms before the lick (solid line) and the 165 ms after the lick (dashed line). We found a reduction in both pre- and post-lick activity during the first three bins, with a more pronounced reduction in pre-lick activity (solid line). This led to increased differences in activity between the post- and pre-lick periods for enhanced cells (Figure 2j, now 2h). This explains why we observed an increase in peak activity when examining the z-scored data in Figure 2e: the larger difference between pre- and post-lick activity translates to higher peaks when the activity is z-scored per cell.

In Bin 4, when the task rules changed, activity increased in both the pre- and post-lick periods. The more pronounced increase in pre-lick activity led to a decrease in the

proportional activity, as seen in Figure 2j (new 2g), and a decrease in the peak of the z-scored data, as seen in Figures 2d-e.

From this analysis, we conclude that there is a general reduction in activity surrounding the lick as the sessions progress. Additionally, the relationship between pre- and post-lick activity changes with experience, which is crucial for understanding how the auditory cortex processes and conveys task-related neural activity.

Importantly, regardless of whether the data is normalized, we found that the change in activity is experience-dependent. Notably, there was a significant reset or change in the activity of enhanced cells when task rules were altered, which was particularly pronounced in the post-sound period.

We have updated the text, figure legends, and graph axes to clarify these findings.

Line 245: We believe there is a missing word: 'increase'? – Yes, thank you. We corrected that accordingly.
Line303: Please remove the extra 'the'. – Done.

<u>Referee #3</u>:

Summary
The authors aim to build on recent studies demonstrating that sensory cortices represent aspects of decision-making in addition to stimuli-related information by asking whether decision-related activity in auditory cortex is innate or acquired throughout learning. Chronic 2-photon imaging allows them to record changes in fluorescent calcium signaling longitudinally while mice learn an auditory detection task with a delayed response window. As they point out, this delayed window is important in helping segregate cue-related activity from decision-related activity and is a step often skipped by other studies. They find that subsets of auditory cortical cells either increase or decrease as animals report the stimuli and that these responses develop and become more synchronized with training. The activity of these subsets can decode whether a lick occurred before or after the stimulus. The paper was very well written, with clear, concise explanations of the experiments and the logic behind them, making it an easy paper to read and understand. My comments on the paper are largely minor comments and the majority pertain to the figures, which I believe could better complement the text with minor alterations.

The main text doesn't describe this, but the task design included catch trials. Performance on these trials was used to calculate d' for Fig 1F. People familiar with psychophysics will understand what d' represents and that the numbers in Fig 1F are

quite good, but others could benefit from a brief explanation in the main text. The methods for calculating this should also be included in the methods.

We extended the explanation of the catch trials to the main text and further described the calculation of the d' in the methods section.

"Licking in catch trials where no sound was presented was counted as a False Alarm, and refraining from licking during the trials where the sound was presented was counted as a Miss. At the end of each trial, there was a silent period lasting between 5 to 10 seconds, randomly chosen from an exponential distribution, preventing the mouse from anticipating when the sound would be played"

"… For Figure 1f, d' was calculated as $d' = z(H) - z(F)$. Where H is the hit rate, and F is the false alarm rate calculated from the catch trials".

Animals are first trained on a Pavlovian task (Bins 1-3) and then trained on an operant task (Bins 4-6). Oftentimes, comparisons were made between Bin 1 and Bin 6, but I wonder whether this is a fair comparison to make given how the task changes between those two bins and how often they see the activity "reset" when the task changes. For example, in Figure 2E there doesn't seem to be a difference between Bin 1 and Bin 6, but there does seem to be a difference between Bin 1 & 3 (first and last bins of Pavlovian) and between Bins 4 & 6 (first and last bins of operant). We agree with the reviewer that besides the difference in activity between Bins 1 & 6, there are changes in activity between Bins 1 & 3, Bins 3 & 4, and between Bins 4 & 6. Each comparison has different meanings. Comparing the activity or behavior between Bins 1 & 3 can help explain what the mice learned in the Pavlovian stage. Comparing the activity and behavior between Bins 3 & 4 allows us to understand what happens when we change the task rules. Comparison between Bins 1 & 6 allows us to explore the changes in activity when the mice have an extended experience with the task.

To further explore the changes in activity, we added in Figure 2e the comparison statistics between Bins 1 & 3, as the reviewer suggested (Fig. 2e, 2-way ANOVA, $F=127.7$ and 126.15, $p=2.05e^{-28}$ and $1.77e^{-28}$ enhanced and suppressed cells). Interestingly, enhanced cell activity increased as the mice gained experience with the task and their environment (post hoc, bin 1 vs bin 6 and bin 1 vs bin 3 post-sound $p<0.001$ and $p=0.01$ Bonferroni corrected) while suppressed cells maintained higher absolute activity during post-sound periods without experience-related modulation (bin 1 vs bin 6 and bin 1 vs bin 3 post-sound $p=1$ Bonferroni corrected).

Figure-related minor points

Generally speaking, the figures could benefit from markers of statistical significance, since a lot of the differences are so subtle but are significant. We attempted to add statistical significance markers to the figures, but they made the visuals too cluttered. Therefore, we opted to describe the statistical significance in the text and figure legends instead.

In Figure 1 and Figure 4, black bars are used with dark data points and black confidence intervals, which makes it hard to read. Open bars might work better to show the other features of the graphs. – We changed the bars to open bars in Fig 1, and we have a new Fig. 5 (original Fig.4).

Figure 1B is not mentioned in the text and it is unclear what kind of session this data is from, Pavlovian or test? Are there differences in the distributions in the two task versions? The original figure in 1B is from a test session. The training sessions don't have different phases. The water always comes at the same time. We made this clearer in the explanation of the task.

More importantly, we have now thoroughly analyzed the lick dynamics and created new lick related figures (please see **new Figures: Fig. 1b, Fig. 1h, Appendix Figure S1b, and Appendix Figure S4a** and the answer to reviewer 1 above) and found that while there are changes in lick dynamics through the task, they are not driving the experience dependent changes in enhance cells.

Figure 2 & Figure 4: Font sizes should be standardized and, in many places, much larger. It was difficult to read much of the text in these figures. Done.

Figure 2: These colors are also very hard to distinguish. The pastels largely look the same, and in many places, the error bars are gray no matter what color the mean line is. The legends are so small that it's hard to tell what is what.

We change the borders of the error bars to the corresponding cell color (red, blue or gray) to make it clearer to distinguish between groups. We also enlarged the legends, titles, and other labels in the figures.

Figure 2E / I / F: The authors repeatedly make the point that the responses seem to reset at the start of Bin 4 when the task rules change. However, here the data is plotted continuously between Bins 3 and 4, which masks that rest a bit. Having a break in the line plot between Bins 3 and 4 would make that reset more obvious while also emphasizing the timepoint where the rules change. Done.

We did the same for all the other figures, which made a big visual difference. Thank you for the suggestion.

Figure 2I: The usage of the word "activity" seems inconsistent across panels. It's unclear which panels represent the activity overall vs. the activity of specific cells, and I think this is an important distinction given how the overall population activity increases while specific cells seem to decrease their responses (if I'm interpreting this correctly). We clarified in the figure and legends when the activity is normalized and when it isn't, and when we are talking about all the cells or a specific population.

Figures 2 & 3: The colors/line styles are very hard to distinguish, especially in panels 2C, 2F, and 3A. Gray is used to denote cells that do not respond to the lick, but the confidence intervals are often gray no matter the response type, which makes the lines blur together.
Figure 4A / 4D: The text says Hit vs LL while the figure says Hit vs Miss. Fixed, thank you.

Figure 4B: I'm not sure whether having all of the individual data points plotted is helpful here. It might be clearer to just have the errorbars. After doing Canonical Correlation Analysis, we have a new Figure 5 (original Fig.4), so that plot is no longer part of the manuscript.

Figure 5A could benefit from a legend, while Figure 5B could benefit from some labels to help denote the difference between the graphs on the top vs the graphs on the bottom. Done

Non-Figure minor points:
Line 42: ACtx is defined later in the main text, but it should be defined here –We corrected that accordingly.
Line 128: incorrect figure reference - maybe 1G? 1B? – Yes, 1G, we corrected that accordingly.

Lines 147-148: What was the logic for picking these two sessions?

We wanted to give an example of the activity in the earlier sessions compared to the latter ones. It takes the animal one or two sessions to understand the link between the sound and the water, so we chose Bin 3 where most of the animals understand that there is a connection. We wanted to compare the activity in this early bin to the activity in the last bin where the mice have the most experience. Following the reviewer's comment, we added more examples in the **new Appendix Figure S2a**.

Line 152: Incorrect figure reference (in general would double check the figure references for Fig 2, sometimes the context of the sentence seemed to better match a different panel in the figure than the one cited) – Thank you. We corrected the references in the text to figure 2.

Line 245: "showing an in the strength" - showing an increase in the strength? – Yes, thank you. We corrected it accordingly.

Line 291: "Distance difference" is only defined in the next sentence, so there might be a clearer way to introduce this here -"difference in the distance between pre- and post-lick periods." – After doing Canonical Correlation Analysis before examining the trajectories of neural population activity across trials, we decided to focus on the distance between the Hit and Late Lick trials, as it is a more straightforward concept than the difference in distances.

Line 364: "time interval", this period is referred to as a delay everywhere else, and could be changed for consistency – we changed the term to delay period, thank you.

1st Revision - Editorial Decision **10th Oct 2024**

Dear Dr. Resnik,

Thank you for the submission of your revised manuscript. We have now received the enclosed reports from the referees and I am happy to say that all support its publication now. Referee 1 still has a few more minor suggestions that I would like you to incorporate before we can proceed with the official acceptance of your manuscript.

A few editorial requests will also need to be addressed:

- Please add up to 5 keywords to the ms file.

- "Resource and data availability" needs to be corrected to "Data Availability Section" (DAS) and needs to be placed at the end of the Methods. This section should only contain links to deposited data. We also need the direct link for the BioStudies deposition of (S-BSST1639) in the DAS.

- The conflict of interest subheading needs to be renamed to "Disclosure Statement and Competing Interests"

- The author credits need to be removed from the ms file. All credits need to be entered during ms submission.

- The REFERENCE FORMAT needs to be alphabetical, not numerical; et al needs to be used after 10 author. The EMBO reports style is also in EndNote.

- Please answer the question about blinding in the author checklist.

- The APPENDIX FILE is missing a table of content with page numbers on the title page

- The Methods section needs to include a (separate) Reagents and Tools Table (listing key reagents, experimental models, software and relevant equipment and including their sources and relevant identifiers). A downloadable template (.docx) for the Reagents and Tools Table can be found in our author guidelines: < https://www.embopress.org/page/journal/14693178/authorguide#manuscriptpreparation

- The manuscript sections should be in the following order: Title page - Abstract & Keywords - Introduction - Results - Discussion - Methods - Data Availability - Acknowledgments - Disclosure Statement & Competing Interests - References - Figure Legends - (Main Tables with legends) - Expanded View Figure Legends.

- Materials and Methods should be Methods

Figure Legends - Comments

- Please note that the exact p values are not provided in the legends of figures 2F, 3.

- Please note that the p value is not represented in the figure 1D, E, 2E, F; 3; 4A; 6A, B, however statistical test related information is provided in the legend of the corresponding figure. This needs to be rectified.

- Please note that information related to n is missing in the legends of figures 1D-H, 4B; 6A.

- Please note that the error bars are not defined in the legends of figures 1H, 3, 4B.

I would like to suggest some minor changes to the abstract that needs to be written in present tense. Please let me know whether you agree with the following:

Associating sensory stimuli with behavioral significance induces substantial changes in stimulus representations. Recent studies suggest that primary sensory cortices not only adjust representations of task-relevant stimuli, but actively participate in encoding decision-making features. We sought to determine whether this trait is innate in sensory cortices or if choice representation develops with time and experience. To trace choice representation development, we perform chronic two-photon calcium imaging in the primary auditory cortex of head-fixed mice while they gain experience in a tone detection task with a delayed decision window. Our results reveal a progressive increase in choice-dependent activity within a specific subpopulation of neurons, aligning with growing task familiarity and adapting to changing task rules. Task experience correlates with heightened synchronized activity in these populations and the ability to differentiate between different types of behavioral decisions. Notably, the activity of this subpopulation accurately decodes the same action at different task phases. Our findings establish a dynamic restructuring of auditory cortex activity to encode decision-making features that develop over time and are refined by experience.

EMBO press papers are accompanied online by A) a short (1-2 sentences) summary of the findings and their significance, B) 2-3 bullet points highlighting key results and C) a synopsis image that is exactly 550 pixels wide and 200-600 pixels high (the height is variable). The synopsis image should provide a sketch of the major findings, like a graphical abstract. Please note that text needs to be readable at the final size. Please send us this information along with the final manuscript.

I look forward to seeing a final version of your manuscript as soon as possible.

Best regards,
Esther

Esther Schnapp, PhD
Senior Editor
EMBO reports

Referee #1:

The authors have made a vary thorough review and detailed response to all the queries I raised. I found that the match between results from low, mid and long lick bursts was quite striking. Looking for example at S4C the average activity is quite similar between the three conditions. This provides an interesting argument in favor of auditory cortex encoding the decision / motor outcome and not fine motor details.
I just had two minor points during reading where I wasn't sure I followed the text exactly and so maybe the authors can clarify a little :

- It seems at some times that the authors consider the 165ms before the lick onset as a baseline (for example line 274 when they point out the reduction in pre-lick activity leads to the increased peak) and sometimes as part of the response to the lick (justified by the ramping up of activity prior to the lick).
- On line 279 the authors use as a control neurons classified as enhanced/suppressed/non on pre-sound data. When I read this I realized I hadn't properly understood that they had defined the previous groups during the post-sound phase only (I though it was all data together). Unless I missed this, could they specify that when they first introduce the first cell groups (line 199)?

Referee #2:

The authors did a wonderful job addressing the comments of all the reviewers. I have no further concerns.

Referee #3:

The authors have addressed my concerns. I have no further comments.

Dear Esther,

1. -<u>Referee 1's final concerns</u>: It seems at some times that the authors consider the 165ms before the lick onset as a baseline (for example line 274 when they point out the reduction in pre-lick activity leads to the increased peak) and sometimes as part of the response to the lick (justified by the ramping up of activity prior to the lick).

In our analysis, we primarily consider the 165 ms preceding the lick as part of the response window. To gain deeper insights into the activity changes, we also separately examined the activity before and after the lick. We found that modulation begins prior to the lick, and the relationship between pre- and post-lick activity shifts significantly (Fig. 2h). This is further elaborated in lines 242-250. *“Having identified distinct responses in various cell sub-populations, we examined with more detail the activity surrounding the lick period, categorizing it by cell type and distinguishing between activity before and after the lick (165 ms before and 165 ms after). A decrease in activity during both pre-lick and post-lick periods emerged as mice gained task experience (Fig. 2g). The more pronounced reduction in pre-lick activity (solid line) led to increased deltas of activity between the post and pre-lick periods for enhanced cells (Fig. 2h 2-way ANOVA, cell types x bin interaction F=16.03, p=1e-28, post-hoc enhanced cells Bin1 vs Bin6 p=0.01, Bonferroni corrected), but a reduction in activity difference for suppressed cells (Fig. 2h, post-hoc Bin1 vs Bin 6 suppressed p=3e-4 Bonferroni corrected). “*
- On line 279 the authors use as a control neurons classified as enhanced/suppressed/non on pre-sound data. When I read this I realized I hadn't properly understood that they had defined the previous groups during the post-sound phase only (I though it was all data together). Unless I missed this, could they specify that when they first introduce the first cell groups (line 199)?

We added a clarification in line 207 when we first divide the cells into sub-groups.

2. Yes, it is all one experiment with N = 6 Mice/ n = 4335 Cells (line 107 at the beginning of the result section). All of the 6 mice did the behavioral task while we imaged the activity in the auditory cortex. The mice did the task for several days (same 6 mice). In some of the analyses, we examine the change in behavior or activity per session, so we added the information to the legend as well.

Let me know if you need more information.

All the best,

Jennifer

Jennifer Resnik, Ph.D.

Senior Lecturer | Department of Life Sciences
Belle and Murray Nathan Career Development Chair in Neurobiology

Zelman Center for Neuroscience | Ben-Gurion University of the Negev

https://ben-gurion.theopenscholar.com/resniklab/

Dr. Jennifer Resnik
Ben-Gurion University
Life Sciences
Ben Gurion blvd 1
84105
Israel

Dear Jennifer,

I am very pleased to accept your manuscript for publication in the next available issue of EMBO reports. Thank you for your contribution to our journal.

Your manuscript will be processed for publication by EMBO Press. It will be copy edited and you will receive page proofs prior to publication. Please note that you will be contacted by Springer Nature Author Services to complete licensing and payment information.

You may qualify for financial assistance for your publication charges - either via a Springer Nature fully open access agreement or an EMBO initiative. Check your eligibility: https://www.embopress.org/page/journal/14693178/authorguide#chargesguide

Should you be planning a Press Release on your article, please get in contact with embo_production@springernature.com as early as possible in order to coordinate publication and release dates.

If you have any questions, please do not hesitate to contact the Editorial Office. Thank you for your contribution to EMBO Reports.

Best,
Esther

Esther Schnapp, PhD
Senior Editor
EMBO reports

>>> Please note that it is EMBO Reports policy for the transcript of the editorial process (containing referee reports and your response letter) to be published as an online supplement to each paper. If you do NOT want this, you will need to inform the Editorial Office via email immediately. More information is available here: https://www.embopress.org/transparent-process#Review_Process

EMBO Press Author Checklist

Corresponding Author Name: Jennifer Resnik	
Journal Submitted to: EMBO reports	
Manuscript Number: EMBOR-2024-59286V1	

USEFUL LINKS FOR COMPLETING THIS FORM
The EMBO Journal - Author Guidelines
EMBO Reports - Author Guidelines
Molecular Systems Biology - Author Guidelines
EMBO Molecular Medicine - Author Guidelines

Reporting Checklist for Life Science Articles (updated January
This checklist is adapted from Materials Design Analysis Reporting (MDAR) Checklist for Authors. MDAR establishes a minimum set of requirements in transparent reporting in the life sciences (see Statement of Task: 10.31222/osf.io/9sm4x). Please follow the journal's guidelines in preparing your
Please note that a copy of this checklist will be published alongside your article.

Abridged guidelines for figures
1. Data
The data shown in figures should satisfy the following conditions:
- the data were obtained and processed according to the field's best practice and are presented to reflect the results of the experiments in an accurate and unbiased manner.
- ideally, figure panels should include only measurements that are directly comparable to each other and obtained with the same assay.
- plots include clearly labeled error bars for independent experiments and sample sizes. Unless justified, error bars should not be shown for technical
- if n<5, the individual data points from each experiment should be plotted. Any statistical test employed should be justified.
- Source Data should be included to report the data underlying figures according to the guidelines set out in the authorship guidelines on Data

2. Captions
Each figure caption should contain the following information, for each panel where they are relevant:
- a specification of the experimental system investigated (eg cell line, species name).
- the assay(s) and method(s) used to carry out the reported observations and measurements.
- an explicit mention of the biological and chemical entity(ies) that are being measured.
- an explicit mention of the biological and chemical entity(ies) that are altered/varied/perturbed in a controlled manner.
- the exact sample size (n) for each experimental group/condition, given as a number, not a range;
- a description of the sample collection allowing the reader to understand whether the samples represent technical or biological replicates (including how many animals, litters, cultures, etc.).
- a statement of how many times the experiment shown was independently replicated in the laboratory.
- definitions of statistical methods and measures:

- common tests, such as t-test (please specify whether paired vs. unpaired), simple χ2 tests, Wilcoxon and Mann-Whitney tests, can be unambiguously identified by name only, but more complex techniques should be described in the methods section;

- are tests one-sided or two-sided?
- are there adjustments for multiple comparisons?
- exact statistical test results, e.g., P values = x but not P values < x;
- definition of 'center values' as median or average;
- definition of error bars as s.d. or s.e.m.

Please complete ALL of the questions below.
Select "Not Applicable" only when the requested information is not relevant for your study.

Materials

Newly Created Materials	Information included in the manuscript?	In which section is the information available? (Reagents and Tools Table, Materials and Methods, Figures, Data Availability Section)
New materials and reagents need to be available; do any restrictions apply?	Not Applicable	

Antibodies	Information included in the manuscript?	In which section is the information available? (Reagents and Tools Table, Materials and Methods, Figures, Data Availability Section)
For **antibodies** provide the following information:<br>- Commercial antibodies: RRID (if possible) or supplier name, catalogue number and or/clone number<br>- Non-commercial: RRID or citation	Not Applicable	

DNA and RNA sequences	Information included in the manuscript?	In which section is the information available? (Reagents and Tools Table, Materials and Methods, Figures, Data Availability Section)
**Short novel DNA or RNA including primers, probes**: provide the sequences.	Not Applicable	

Cell materials	Information included in the manuscript?	In which section is the information available? (Reagents and Tools Table, Materials and Methods, Figures, Data Availability Section)
**Cell lines:** Provide species information, strain. Provide accession number in repository **OR** supplier name, catalog number, clone number, and/**OR** RRID.	Not Applicable	
**Primary cultures:** Provide species, strain, sex of origin, genetic modification status.	Not Applicable	
Report if the cell lines were recently **authenticated** (e.g., by STR profiling) and tested for mycoplasma contamination.	Not Applicable	

Experimental animals	Information included in the manuscript?	In which section is the information available? (Reagents and Tools Table, Materials and Methods, Figures, Data Availability Section)

**Laboratory animals or Model organisms:** Provide species, strain, sex, age, genetic modification status. Provide accession number in repository **OR** supplier name, catalog number, clone number, **OR** RRID.	Yes	In the methods and result sections
**Animal observed in or captured from the field:** Provide species, sex, and age where possible**.**	Not Applicable	
Please detail **housing and husbandry conditions**.	Yes	Methods section

**Plants and microbes**	**Information included in the manuscript?**	**In which section is the information available?** (Reagents and Tools Table, Materials and Methods, Figures, Data Availability Section)
**Plants:** provide species and strain, ecotype and cultivar where relevant, unique accession number if available, and source (including location for collected wild specimens).	Not Applicable	
**Microbes:** provide species and strain, unique accession number if available, and source.	Not Applicable	

**Human research participants**	**Information included in the manuscript?**	**In which section is the information available?** (Reagents and Tools Table, Materials and Methods, Figures, Data Availability Section)
If collected and within the bounds of privacy constraints report on age, sex and gender or ethnicity for all study participants.	Not Applicable	

**Core facilities**	**Information included in the manuscript?**	**In which section is the information available?** (Reagents and Tools Table, Materials and Methods, Figures, Data Availability Section)
If your work benefited from core facilities, was their service mentioned in the acknowledgments section?	Not Applicable	

Design

**Study protocol**	**Information included in the manuscript?**	**In which section is the information available?** (Reagents and Tools Table, Materials and Methods, Figures, Data Availability Section)
If study protocol has been **pre-registered, provide DOI in the manuscript**. For clinical trials, provide the trial registration number **OR** cite DOI.	Not Applicable	
Report the **clinical trial registration number** (at ClinicalTrials.gov or equivalent), where applicable.	Not Applicable	

**Laboratory protocol**	**Information included in the manuscript?**	**In which section is the information available?** (Reagents and Tools Table, Materials and Methods, Figures, Data Availability Section)
Provide DOI OR other citation details if **external detailed step-by-step protocols** are available.	Not Applicable	

**Experimental study design and statistics**	**Information included in the manuscript?**	**In which section is the information available?** (Reagents and Tools Table, Materials and Methods, Figures, Data Availability Section)
Include a statement about **sample size** estimate even if no statistical methods were used.	Yes	Results and methods section
Were any steps taken to minimize the effects of subjective bias when allocating animals/samples to treatment (e.g. **randomization procedure**)? If yes, have they been described?	Not Applicable	
Include a statement about **blinding** even if no blinding was done.	Not Applicable	methods
Describe **inclusion/exclusion criteria** if samples or animals were excluded from the analysis. Were the criteria pre-established?<br><br>If sample or data points were omitted from analysis, report if this was due to attrition or intentional exclusion and provide justification.	Not Applicable	
For every figure, are **statistical tests** justified as appropriate? Do the data meet the assumptions of the tests (e.g., normal distribution)? Describe any methods used to assess it. Is there an estimate of variation within each group of data? Is the variance similar between the groups that are being statistically compared?	Yes	Results and methods section

**Sample definition and in-laboratory replication**	**Information included in the manuscript?**	**In which section is the information available?** (Reagents and Tools Table, Materials and Methods, Figures, Data Availability Section)
In the figure legends: state number of times the experiment was **replicated** in laboratory.	Not Applicable	
In the figure legends: define whether data describe **technical or biological replicates**.	Not Applicable	

Ethics

**Ethics**	**Information included in the manuscript?**	**In which section is the information available?** (Reagents and Tools Table, Materials and Methods, Figures, Data Availability Section)
Studies involving **human participants**: State details of **authority granting ethics approval** (IRB or equivalent committee(s), provide reference number for approval.	Not Applicable	
Studies involving **human participants**: Include a statement confirming that **informed consent** was obtained from all subjects and that the experiments conformed to the principles set out in the WMA Declaration of Helsinki and the Department of Health and Human Services Belmont Report	Not Applicable	

Studies involving **human participants:** For publication of **patient photos**, include a statement confirming that consent to publish was obtained.	Not Applicable	
Studies involving experimental **animals**: State details of **authority granting ethics approval** (IRB or equivalent committee(s), provide reference number for approval. Include a statement of compliance with ethical regulations.	Yes	Methods section
Studies involving **specimen and field samples:** State if relevant **permits** obtained, provide details of authority approving study; if none were required, explain why.	Not Applicable	

Dual Use Research of Concern (DURC)	Information included in the manuscript?	In which section is the information available? (Reagents and Tools Table, Materials and Methods, Figures, Data Availability Section)
Could your study fall under dual use research restrictions? Please check biosecurity documents and list of **select agents and toxins** (CDC): https://www.selectagents.gov/sat/list.htm	Not Applicable	
If you used a select agent, is the security level of the lab appropriate and reported in the manuscript?	Not Applicable	
If a study is subject to dual use research of concern regulations, is the name of the **authority granting approval and reference number** for the regulatory approval provided in the manuscript?	Not Applicable	

Reporting

The MDAR framework recommends adoption of discipline-specific guidelines, established and endorsed through community initiatives. Journals have their own policy about requiring specific guidelines and recommendations to complement MDAR.

Adherence to community standards	Information included in the manuscript?	In which section is the information available? (Reagents and Tools Table, Materials and Methods, Figures, Data Availability Section)
State if relevant guidelines or checklists (e.g., **ICMJE, MIBBI, ARRIVE, PRISMA**) have been followed or provided.	Not Applicable	
For **tumor marker prognostic studies,** we recommend that you follow the **REMARK** reporting guidelines (see link list at top right). See author guidelines, under 'Reporting Guidelines'. Please confirm you have followed these guidelines.	Not Applicable	
For **phase II and III randomized controlled trials**, please refer to the **CONSORT** flow diagram (see link list at top right) and submit the CONSORT checklist (see link list at top right) with your submission. See author guidelines, under 'Reporting Guidelines'. Please confirm you have submitted this list.	Not Applicable	

Data Availability

Data availability	Information included in the manuscript?	In which section is the information available? (Reagents and Tools Table, Materials and Methods, Figures, Data Availability Section)
Have **primary datasets** been deposited according to the journal's guidelines (see 'Data Deposition' section) and the respective accession numbers provided in the Data Availability Section?	Yes	Methods section
Were **human clinical and genomic datasets** deposited in a public access-controlled repository in accordance to ethical obligations to the patients and to the applicable consent agreement?	Not Applicable	
Are **computational models** that are central and integral to a study available without restrictions in a machine-readable form? Were the relevant accession numbers or links provided?	Not Applicable	
If publicly available data were reused, provide the respective **data citations in the reference list**.	Not Applicable	
